# Red novae, their progenitors, and remnants

**DOI:** 10.1007/s00159-026-00173-y

**Published:** 2026-10-03

**Authors:** Tomasz Kamiński, Nadejda Blagorodnova

**Affiliations:** 1 Laboratory for Astrophysics I, Nicolaus Copernicus Astronomical Center, ul. Rabiańska 8, 87-100 Toruń, Kujawsko-Pomorskie Poland; 2 Institut de Ciències del Cosmos (ICCUB), Universitat de Barcelona (UB), c. Martí i Franquès, 1, 08028 Barcelona, Spain; 3 Departament de Física Quàntica i Astrofísica (FQA), Universitat de Barcelona (UB), c. Martí i Franquès, 1, 08028 Barcelona, Spain; 4 Institut d’Estudis Espacials de Catalunya (IEEC), Edifici RDIT, Campus UPC, 08860 Castelldefels, Spain

**Keywords:** Transients, Stellar mergers, Common envelope evolution, Binary evolution, Circumstellar matter

## Abstract

Red novae or luminous red novae are a class of optical transients that have emerged over the past two decades. They occupy an intermediate-luminosity regime between classical novae and supernovae and are characterized by cool, slowly expanding ejecta and a pronounced evolution toward red, dust-enshrouded remnants. These events are now widely interpreted as the outcome of binary coalescence involving non-compact stars, providing a rare opportunity to directly observe the dynamical phases of stellar mergers and their immediate aftermath. Observational studies of red novae provide a glimpse into the still poorly understood physics of unstable mass transfer and common-envelope evolution in binary stars, responsible for the formation of high-energy astrophysical phenomena, compact binary systems, and gravitational-wave sources. In this review, we synthesize current observational knowledge of red novae, including their outburst properties, population characteristics, and long-term remnants. Observations of light curves, spectra, and circumstellar environments reveal a complex interplay between mass ejection, collisions, radiative processes, and dust formation. Archival detections of red novae progenitors show a diversity of systems, ranging from low-mass contact binaries to massive evolved stars, with a notable representation of post-main-sequence stars. We also examine current efforts to predict red nova outbursts and establish robust event rates in the Local Universe, both of which remain challenging. Finally, we outline future prospects, including the impact of large-scale time-domain surveys and the potential connection between stellar mergers and gravitational-wave sources.

## Introduction

Red novae (RNe) and luminous red novae (LRNe) are a class of optical transients recognized over the past 2 decades as a distinct subgroup of intermediate-luminosity events. They typically span peak magnitudes of $-3 \gtrsim M_r \gtrsim -16$ mag and evolve on timescales of weeks to years. Despite this diversity, they share several defining characteristics: low effective temperatures at maximum light, outflow velocities of a few hundred $\text {km s}^{-1}$, and rapid post-peak cooling that produces a red, often dust-enshrouded remnant. These properties distinguish them from other transients in a similar luminosity range.

Red nova eruptions are non-terminal and are widely interpreted as the observable outcome of stellar mergers in binaries composed of non-compact stars, from main-sequence (MS) objects to evolved giants and supergiants. This interpretation was firmly established with V1309 Sco, whose progenitor was observed as a contact binary prior to merger. Red novae therefore provide a direct window into the physics of extreme binary interaction, including common-envelope and grazing-envelope evolution—processes that remain central, yet poorly understood, in stellar astrophysics and population synthesis. Although only ≃ 29 events are currently known, the volumetric rate of the more luminous events in the Local Group is comparable to that of core-collapse supernovae (and even higher for low-mass red nova events), and their detection rate is expected to increase rapidly with ongoing and upcoming time-domain surveys.

In this review, we summarize observational progress in the study of red novae, building on earlier works focused on their phenomenology (e.g., Kamiński et al. 2018a; Pastorello et al. 2019b). We emphasize the connection between observed properties and the underlying physics of stellar mergers, while referring the reader to dedicated reviews for theoretical aspects of common-envelope evolution and merger modeling (e.g., Ivanova et al. 2013a; Röpke and De Marco 2023; Schneider 2025).

This paper is organized as follows. Section 2 introduces the red nova phenomenon and its historical context. Section 3 describes their photometric and spectroscopic evolution during outburst. Section 4 reviews the properties of progenitor systems and methods of their identification. Section 5 discusses population trends and event rates. Section 6 summarizes the properties of red nova remnants. Finally, Sect. 7 outlines future directions, including prospects for detecting gravitational waves from selected systems.

## The red nova phenomenon

### Historical perspective and milestones

While unusually red transients have been sporadically observed in the 20th century, the modern field of red novae began with the enigmatic eruption of V838 Mon in early 2002. Initially considered a classical nova, V838 Mon defied expectations for all known nova-like objects and transients at the time (Munari et al. 2007). Additionally, a spectacular light echo from the eruption was captured by the Hubble Space Telescope (Bond et al. 2003), making V838 Mon an especially attractive target for study. It was noted early on that the unusual properties of V838 Mon’s outburst matched well with two earlier outbursts, that is, of the Milky Way object V4332 Sgr, which erupted in 1994 (Martini et al. 1999), and of the Red Variable, which erupted in the Andromeda Galaxy in 1988 (M31-RV; Mould et al. 1990). The latter was particularly perplexing for the contemporary observers, but was assumed to be an atypical outburst of a red giant star (Rich et al. 1989). Only the renewed interest, triggered by studies of V838 Mon in the early 2000s, changed this view. V838 Mon, M31-RV, and V4332 Sgr were quickly associated with each other, and it was realized that they belong to an entirely new type of astrophysical eruption, most commonly known today as luminous red novae (LRNe). It took another 2 decades to link red novae to an even older eruption, CK Vul, which was observed by European astronomers in 1670–1672 (Hevelius 1671; Shara et al. 1985; Kato 2003; Kamiński et al. 2015b). While the observations of red novae can be dated back to the late 1980s or even the seventeenth century, the current interpretation of the phenomenon—as a manifestation of stellar coalescence observed in real time—dates to the fundamental work of Tylenda and Soker (2006). They demonstrated that collisions between non-compact stars can trigger optical outbursts of relatively high luminosity and that such events would be similar to V838 Mon-like eruptions.

**Fig. 1:**
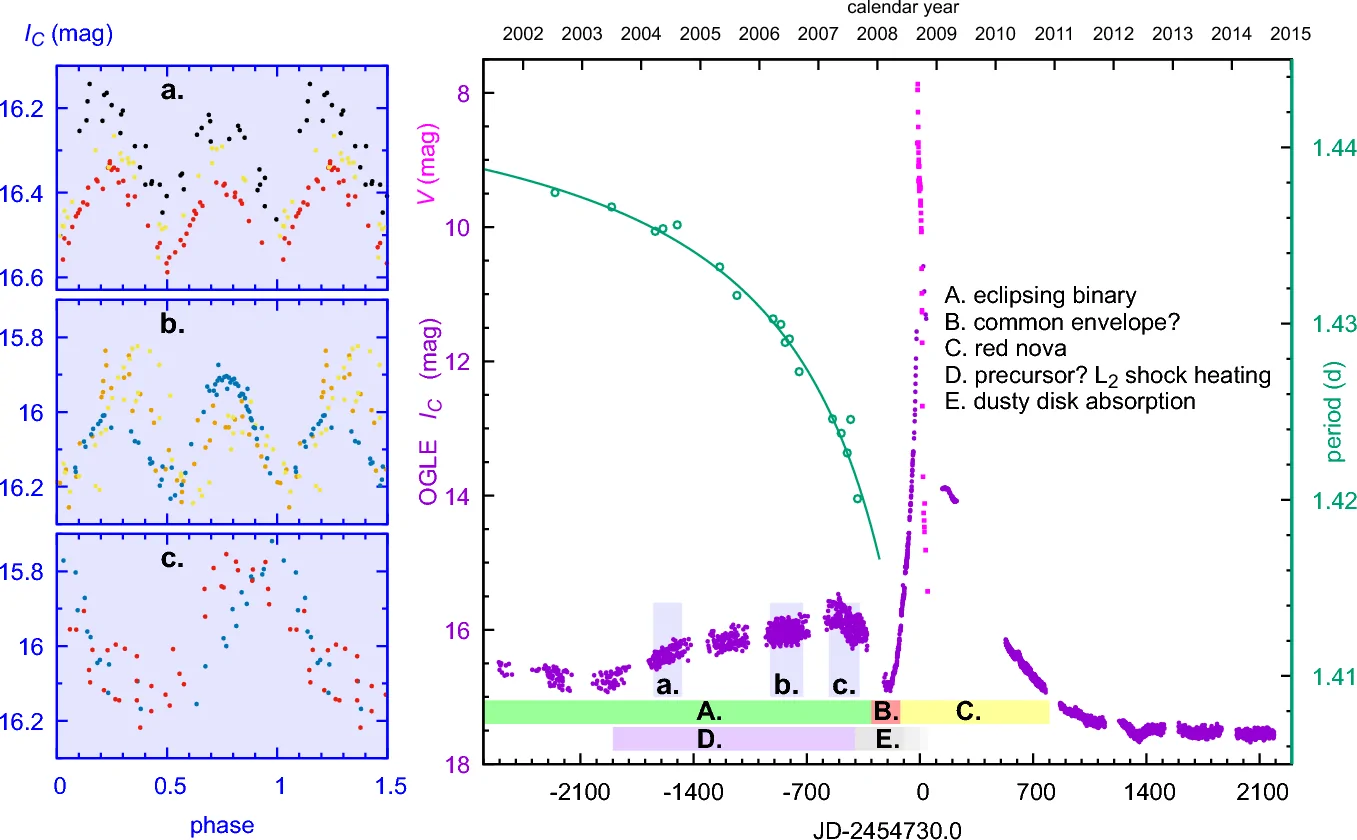
The light curve of V1309 Sco before and during the outburst. The main panel shows photometric measurements (purple and magenta points for $I_C$ and *V*, respectively) and the derived period (green points). The main phases in V1309 Sco photometric evolution are indicated by color bars at the bottom of the plot. Sample phased light curves are shown in the three left panels and correspond to the shaded areas **a**, **b**, and **c** in the main panel. Points of different colors in the smaller panels show individually phased curves for selected time ranges. The reference epoch was arbitrarily selected but is common to all presented curves. Based on Tylenda et al. (2011a) and Pejcha et al. (2017) (and references therein). Data from OGLE (*V* and $I_C$), ASAS (*V*), and AAVSO (*V*)

A major breakthrough in the investigations of red novae was the discovery of Nova Sco 2008, now known as V1309 Sco, which underwent an outburst in late 2008 (Mason et al. 2010). The progenitor system was photometrically monitored by the OGLE project (Udalski et al. 2015), whose observations showed an eclipsing contact binary with a decaying orbital period (Tylenda et al. 2011a, see Fig. 1). This was the first time the spiraling-in of two stars into a common-envelope phase was observed, predating the first detection of gravitational waves from in-spiraling black holes by a few years. V1309 Sco provides the most direct and compelling evidence that collisions between non-compact stars produce moderately energetic eruptions visible at optical and infrared (IR) wavelengths.

Subsequent discoveries of more luminous extragalactic red novae have significantly expanded the known sample. For events within a few Mpc, progenitor systems have been identified in archival data—including M31-LRN-2015, M101-2015-OT, and NGC4490-2011OT1—extending the population to higher-mass progenitors. In addition, several previously classified *supernova impostor*–like events have been reinterpreted as red novae. The current sample discussed in the literature is summarized in Table 1.

**Table 1:** Red novae classified until 2026 and their basic parameters

Name	Distance	Host	Year of	LC	Plateau	$L_\textrm{peak}$	Peak abs. mag	FWHM	$M_\textrm{dust}$	Note	Main
	(kpc,Mpc)	galaxy	main peak	type	(d)	($\textrm{L}_{\odot }$)	Band Mag±Err	($\text {km s}^{-1}$)	($\textrm{M}_{\odot }$)		ref.
(1)	(2)	(3)	(4)	(5)	(6)	(7)	(8)	(9)	(10)	(11)	(12)
CK Vul	2.6−3.5	Milky Way	1671	yes R 3	∼ 600	6.2E6	$M_V\;-12.3 \pm 0.6$	1420	1.0E–2	^1^	[1]
V4332 Sgr	4.2−5.5	Milky Way	1994	yes P 4-5	200	4.5E4	$M_V\;-7.9 \pm 0.6$	240	6.0E–4	^2^	[2]
V838 Mon	5.9	Milky Way	2002	yes R 3-4	76	6.9E5	$M_R\;-10.1 \pm 0.2$	<250	9.4E-4	^3^	[3]
BLG360	4.1−5.6	Milky Way	2004	yes R 3	>1000	7.0E5	$M_I\;-3.3 \pm 0.3$	∼100	1.2E–2	^4^	[4]
V1309 Sco	2−5.6	Milky Way	2008	yes P 2	27	3.2E4	$M_V\;-7.0 \pm 1.4$	300	1.0E–3	^5^	[5]
ZTF SLRN-2020	4	Milky Way	2020	yes? P? 2	0	34	$M_r\;+2.2 \pm 1.0$	120	1.3E–6	^5^	[6]
M31-RV	0.78	M31	1988	? P? 1?	≥60	7.5E5	$M_{R_c}\;-9.9 \pm 0.2$	460		^7^	[7]
M31-LRN-2015	0.78	M31	2015	yes P 1-2	41	8.7E5	$M_r\;-9.8 \pm 0.4$	200	7.7E–4		[8]
AT2019zhd	0.78	M31	2019	yes P 2-3	26	7.0E5	$M_r\;-9.6 \pm 0.1$	280			[9]
AT2025abao	0.78	M31	2025	? R 2-3	100	9.1E5	$M_r\;-9.8 \pm 0.1$	700	4.0E–7	^8^	[10]
AT1997bs	9.2	NGC 3627	1997	? U 2	140	1.6E7	$M_R\;-13.7 \pm 0.2$	585	1.0E–3	^9^	[11]
AT2007sv	18.9	UGC 5979	<2007	? P 2	100	3.0E7	$M_R\;-14.3 \pm 0.4$	800		^10^	[12]
N4490-OT2011	9.6	NGC 4490	2011	yes R 2	210	3.0E7	$M_R\;-14.4 \pm 0.3$	350	1.8E–3	^11^	[13]
N3437-2011OT1	18.6	NGC 3437	2011	no R 2	147	1.0E7	$M_R\;-13.9 \pm 0.4$	350		^12^	[14]
UGC12307-2013-OT1	39.7	UGC12307	2013	no R ≥1	148	3.0E7	$M_R\;-15.0 \pm 0.2$	400		^13^	[15]
SNhunt248	22.5	NGC 5806	2014	yes R >2	154	3.5E7	$M_R\;-15.1 \pm 0.4$	440	(1:10)E–5	^14^	[16]
AT2014ej	20.6	NGC7552	2014	? R 2	83	7.7E7	$M_r\;-15.1 \pm 0.5$	900			[17]
M101-2015OT1	6.4	M101	2015	yes R 2-3	230	6.0E6	$M_R\;-12.4 \pm 0.5$	500		^15^	[18]
AT2015fx	23.8	NGC 2748	2015	? P ≥2	120	3.3E8	$M_r\;-13.6 \pm 0.2$	700		^16^	[19]
AT2017jfs	34.7	NGC 4470	2017	? R 2	176	1.4E8	$M_r\;-15.6 \pm 0.2$	450		^17^	[20]
AT2018bwo	6.6	NGC 45	2018	? P 1?	41	2.6E6	$M_r\;-10.9 \pm 0.1$	580	7.5E–5		[21]
AT2018gzz	66.9	NGC 271	2018	? R 2?	115	4.9E7	$M_r\;-14.5 \pm 0.2$	280			[22]
AT2018hso	20.2	NGC 3729	2018	yes? R 2	210	2.8E7	$M_r\;-13.9 \pm 0.1$	400		^18^	[23]
AT2020hat	5.2	NGC 5068	2020	yes P 1-2	100	2.0E6	$M_r\;-11.0 \pm 0.1$	250		^19^	[24]
AT2020kog	23.8	NGC 6106	2020	? P 2?	120	2.3E7	$M_r\;-13.2 \pm 0.5$	380			[25]
AT2021afy	48.7	UGC 10043	2021	? P >2	120	5.3E7	$M_r\;-14.6 \pm 0.6$	410			[26]
AT2021biy	7.5	NGC 4631	2021	? R 3	210	4.1E6	$M_r\;-13.9 \pm 0.2$	430	3.0E–4	^21^	[27]
AT2021blu	8.6	UGC 5829	2021	? R 2	242	1.8E7	$M_r\;-13.3 \pm 0.2$	470	4.2E–5	^20^	[28]
AT2021aess	34.4	NGC 1359	2021	? R >2	150	3.5E7	$M_r\;-15.1 \pm 0.2$	470		^22^	[29]

Columns: Col.(2) Distance is given in kpc for Milky Way objects and in Mpc otherwise. Col.(4) Year of the main peak, not necessarily of the first peak. Col.(5) The light curve type is coded by three aspects: whether the light curve has the precursor feature, light-curve shape defined in text (P and R for ‘plateau group’ and ‘risers group’, respectively), and the number of distinct peaks in multi-wavelength observations (arbitrary). Col.(6) The time span of the plateau phase in the (visual) light curve. Col.(8) See original papers for error derivations. Col.(9) Line FWHM as a proxy of the main ejecta velocity. When possible, measurements for metal lines were used, avoiding Lorentzian hydrogen emission wings. Col.(10) Highest reported dust mass (may include pre-eruption dust). Col.(12) Main reference, esp. justifying red nova classification

**Comments:** ^1^misclassified as a hibernating classical nova; ^2^misclassified as classical nova; ^3^misclassified as classical novae; ^4^misclassified as a lensing event; no spectrum taken in outburst; ^5^misclassified as classical nova; ^6^YSO?,a.k.a. ZTF 20aazusyv; ^7^no linewidth measurement; remnant not recovered; ^8^still in outburst; a.k.a WNTR23BZDIQ; ^9^misclassified as SN impostor; ^10^misclassified as SN impostor; ^11^a.k.a. AT2011kp; ^12^a.k.a. SNhunt31; ^13^a.k.a. AT2013lw; ^14^probably not a RN; a.k.a. AT2014ib; ^15^a.k.a. AT2015dl; ^16^misclassified as SN impostor; ^17^misclassified as SN IIn; ^18^misclassified as ILRT; ^19^infrared precursor; ^20^misclassified as LBV; ^21^GEE?; ^22^a.k.a. ZTF21acpkzcc;

References: [1] Kato (2003); [2] Martini et al. (1999); [3] Tylenda (2005); [4] Tylenda et al. (2013); [5] Tylenda et al. (2011a); [6] De et al. (2023); [7] Boschi and Munari (2004); [8] Williams et al. (2015); [9] Pastorello et al. (2021a); [10] Karambelkar et al. (2025); [11] Pastorello et al. (2019b); [12] Reguitti et al. (2026a); [13] Smith et al. (2016) [14] Pastorello et al. (2019b); [15] Pastorello et al. (2019b); [16] Kankare et al. (2015); [17] Stritzinger et al. (2014); [18] Blagorodnova et al. (2017); [19] Reguitti et al. (2026a); [20] Pastorello et al. (2019b); [21] Blagorodnova et al. (2021); [22] Karambelkar et al. (2023); [23] Cai et al. (2019); [24] Pastorello et al. (2021b); [25] Pastorello et al. (2021b); [26] Pastorello et al. (2023); [27] Cai et al. (2022b); [28] Pastorello et al. (2023); [29] Karambelkar et al. (2023)

### Naming conventions

The first time the term ‘red nova’ (RN) was used in the literature to describe the phenomenon known today under this name was by Goranskii and Barsukova (2007) and in multiple conference proceedings in Corradi and Munari (2007). These early studies referred to both Galactic (V838 Mon and V4332 Sgr) and extragalactic objects (M31-RV). The extended term ‘luminous red nova’, coined later, was used first to reference a particularly luminous extragalactic red nova in M85 (Kulkarni et al. 2007; Rau et al. 2007). However, the terms RN and LRN later became interchangeable, regardless of the actual luminosity of the transient. Additionally, the recent recognition of planetary-engulfment events as red novae led to the introduction of the term ‘underluminous red nova’ (De et al. 2023). It is important to note that distances and true bolometric luminosities of Galactic objects are often poorly constrained, so the quantification in the naming may be confusing or incorrect.

Several groups have used alternative naming conventions, such as red transients, mergebursts, and tylendars. In the extragalactic transient literature, red novae are commonly included in the broader ‘gap transient’ population introduced by Kasliwal (2012). This group is a mixed bag of diverse astrophysical events in the luminosity gap between novae and supernovae (SNe). For example, it includes intrinsically faint, non-interacting supernovae, such as faint type IIP, type Iax, and Ca-rich events, each having the characteristic spectroscopic signature of their transient family, such as broad hydrogen lines, thermonuclear spectra, or Ca-strong emission lines. In addition, it includes the so-called ‘interacting gap transients’ (Cai et al. 2022a), containing LRNe and other families such as ILRT or SN impostors, which are discussed in more detail below. These events share broadly similar early-time observational characteristics: they display moderate color temperatures at peak (< 15,000 K) and their spectra are initially dominated by narrow absorption and emission features, usually narrower than a few hundred $\text {km s}^{-1}$ and associated primarily with hydrogen and low-ionization elements (Fe ii, Ti ii, Ca ii, Na i, etc.).

**Fig. 2:**
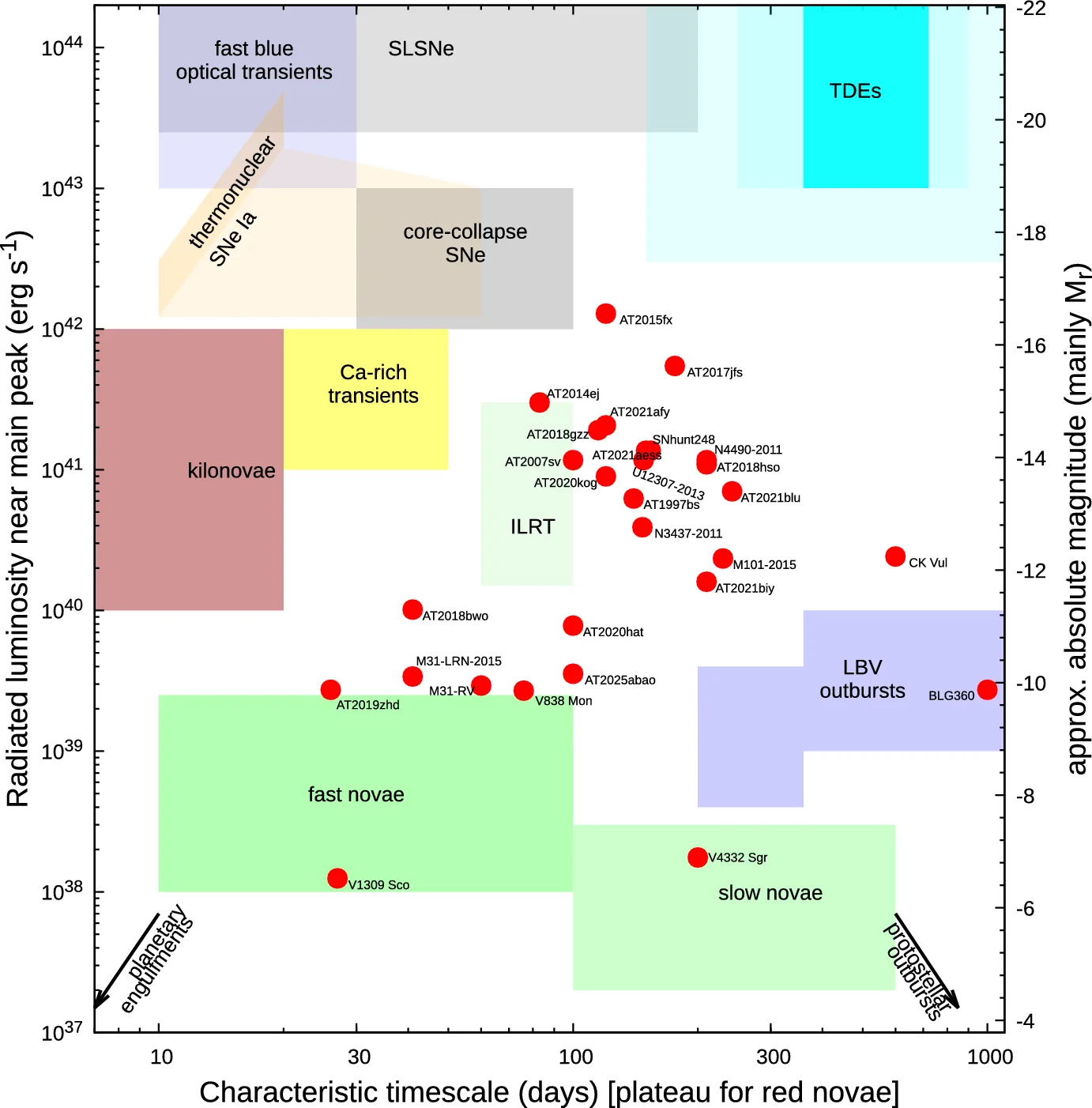
Peak luminosities vs. duration parameter space for most known red novae (red circles) in the context of other astrophysical transients (shaded regions). For red novae, the plotted timescale is the plateau time span from Table 1. For other types of transients, this timescale is obtained rather arbitrarily, usually as the duration of the plateau or the decay time from peak by 2–3 magnitudes. Note that the distances to most Galactic red novae are poorly constrained, making their luminosities especially uncertain. For a variation of the diagram where total energy is considered (i.e., including kinetic energy), see, for instance, Kashi and Soker (2010)

Initially, some authors also termed LRNe and other interacting gap transients as ‘intermediate-luminosity optical transients’ (ILOTs) or ‘intermediate-luminosity *red* transients’ (ILRTs) (Pastorello and Fraser 2019), and in some works this name is still used as a synonym of ‘gap transient’ (e.g., Soker and Kashi 2016). However, recently the term ‘ILRT’ was redefined, and currently ILRTs constitute a narrower class within the ‘interacting gap transient’ population, which is typically represented by NGC 300-OT and SN 2008 S-like events (Thompson et al. 2009; Valerin et al. 2025a, b). Thus, ILOTs include ILRTs, but the two terms are not synonymous. When detected, ILRT progenitors have generally been dust-enshrouded stars, with recent studies suggesting carbon-rich progenitors in at least some cases (Rose et al. 2025). Follow-up studies of these outbursts in the infrared have shown that at late times the sources faded below their progenitor luminosities (Prieto et al. 2008; Berger et al. 2009; Adams et al. 2016; Valerin et al. 2025a), supporting a terminal-explosion interpretation. Ironically, the transient in M85 that originally gave rise to the term *luminous red nova* was later reclassified as an ILOT, based on the identification of an IR-bright progenitor (Ofek et al. 2008). Nowadays, the terms ILOT and ILRT are almost exclusively used to refer to these weak explosions, which are likely from electron-capture SNe (e.g., Botticella et al. 2009; Kochanek 2011; Cai et al. 2021, 2022a).

‘Supernova impostors’ are also commonly placed within the gap transient population (Van Dyk et al. 2000, 2002; Kochanek et al. 2012) and partially overlap with the parameter space of red novae. Observationally, SN impostors are characterized by narrow hydrogen emission lines which are narrower than in genuine SNe, i.e., with full width at half maximum, FWHM, <2000 $\text {km s}^{-1}$. Early phases closely resemble type IIn SNe (Filippenko 1997), which led to initial misclassifications as genuine supernovae. Impostor spectra also resemble the most energetic red novae at early stages, making initial classification challenging and leading to misclassifications and confusion among these three classes. Indeed, some SN impostors (e.g., SN 1997bs, AT 2007sv, AT 2015fx; Van Dyk et al. 2000; Tartaglia et al. 2015, 2016) were reclassified as red novae based on spectroscopic and photometric evolution at optical and IR wavelengths (Pastorello et al. 2019b; Reguitti et al. 2026a). Following Kasliwal (2012), our Fig. 2 illustrates the relation of red novae to other (gap) transients in terms of their radiative luminosity and characteristic duration of the outburst.

Although the class of supernova impostors defines objects with broadly similar observational signatures, it actually represents a heterogeneous group of transients whose outbursts may be powered by distinct physical mechanisms (Smith et al. 2011; Van Dyk and Matheson 2012). In some cases, supernova impostors have been observed to precede a genuine supernova explosion, such as SN 2009ip (Mauerhan et al. 2013; Pastorello et al. 2013; Margutti et al. 2014; Soker and Kashi 2013), leading to their interpretation as supernova precursors. Other supernova impostors were claimed to be giant eruptions of luminous blue variables (LBVs) originating from very massive progenitors (M>40 $\hbox {M}_{\odot }$), such as P Cyg and η Car (Davidson and Humphreys 1997; Weis and Bomans 2020). One observational property that helps set them apart from other classes, such as red novae, is their significantly longer timescale: their outbursts typically persist for several hundred days (e.g., η Car or UGC 2773-OT; Smith et al. 2009) and, in some cases, recur (e.g., P Cyg or SN 2000ch; Wagner et al. 2000).

One final variation of red novae is ‘especially red intermediate-luminosity transient events’ (SPRITEs) (Kasliwal et al. 2017; Jencson et al. 2019b), which are events detected at IR wavelengths, with only weak or non-detected visual counterparts. Unfortunately, the different names and classifications are not used consistently in the field, and the re-classification of historic events is still ongoing.

We use here the simplified but more generic name *red novae* in reference to all transients of this type, regardless of their luminosity, location (Galactic and extragalactic), or whether they were observed solely in the optical or IR (i.e., SPRITEs are included). In this naming scheme, ‘red’ reflects the property that the immediate product of the outburst is an intrinsically cool star, often of spectral type M, which is additionally reddened by circumstellar dust formed from the ejecta. As will be discussed later, dust obscuration is so high that it would be more adequate to call them ‘infrared’ novae. ‘Nova’ may be thought of as having the original ancient meaning, that is, ‘a new star in the sky’, which relates to the transient nature of the objects. ‘Nova’ also seems to be an adequate term since, in the merger interpretation, a rejuvenated star with new properties is formed.

### The merger interpretation of the red novae

Soon after the eruption of V838 Mon, a discussion on the nature of this and similar transients began. Multiple scenarios were proposed, including an atypical nova-like outburst (e.g., Rauch et al. 2002; Shara et al. 2010); helium-shell flashes in an AGB star or post-AGB star (including a born-again AGB star Lawlor 2005); and a consecutive engulfment of several planets by the parent star (Retter et al. 2006). Tylenda and Soker (2006) (see also Soker and Tylenda 2006; Tylenda 2005) critically examined these scenarios and showed that they fail to consistently account for the observed characteristics of V838 Mon and similar objects. Some proposed scenarios fail to reproduce the observed outburst luminosities and progenitor brightnesses, fail to predict chemical abundances consistent with observations, or do not match the inferred timescales, ejecta velocities, and ejecta masses based on the best estimates available at the time. Most characteristically, many of the considered eruption mechanisms are primarily powered by thermonuclear burning, which requires very high temperatures and which consequently results in a very hot remnant. For instance, classical novae reach temperatures above $10^5$ K after the eruption. As discussed later, red novae evolve to very low photospheric temperatures, reaching values as low as ∼ 2000 K, and are embedded in circumstellar material spanning temperatures of ∼ 10–1000 K. These properties are inconsistent with expectations for thermonuclear outburst models.

The merger model proposed by Tylenda and Soker (2006) remains the most successful framework for explaining V838 Mon and other red novae discovered since then. The eruption model developed for V838 Mon envisioned a collision in a binary consisting of a B3 main-sequence star (6–8 $\hbox {M}_{\odot }$) and a protostar of ≈0.4 $\hbox {M}_{\odot }$ (Tylenda et al. 2005, see also Sect. 4.1). Later on, it was also found that the binary had a tertiary B-type companion at a wide orbit. The collision and subsequent coalescence in V838 Mon released the gravitational energy of the inner binary system. Part of the energy was used for heating the gas in shocks and powering the electromagnetic outburst, whereas an approximately similar energy fraction was used for unbinding matter from the system. Their interpretation was that the event was thus powered mainly by accretion and did not require sustained thermonuclear burning at high temperatures.

In the framework proposed by Tylenda et al. (2005), after the secondary had plunged into the primary’s envelope, the energy released beneath the surface of the primary increased the internal pressure driving the rapid expansion of the star and optically thick winds (Lipunov et al. 2017; Quataert et al. 2016). This formed an enormously bloated object with a low surface temperature. The scenario explains very well the observed properties of V838 Mon during its 3-month outburst and the following and ongoing relaxation phase. Later studies of red novae and similar systems added another layer of complexity to the energetics of collisions by considering the contribution of hydrogen and helium recombination energy (related to the internal energy of the gas) to power the observed luminosity (e.g., Ivanova et al. 2013b, 2015; Matsumoto and Metzger 2022; Chen and Ivanova 2024).

Overall, the energetics of common-envelope (CE) remain complex. For example, simulations of low-mass giants, for which OGLE-360 remains the only observational candidate, show that recombination energy could also enhance the fraction of unbound material, leading to more efficient envelope ejection (Reichardt et al. 2020). In addition, studies of high-mass systems suggest that even the orbital and recombination energy released during inspiral may not be sufficient to explain the formation of observed X-ray binaries, pointing to the need for additional energy sources (Li et al. 2026). Suggested mechanisms include brief phases of nuclear energy release (Podsiadlowski et al. 2010; Tylenda et al. 2024) and accretion-driven jets (Soker 2004), among others. However, because these investigations have been restricted to a limited number of case studies, it remains premature to assess the global importance of these processes for the wider binary population. To date, the energy budget of common-envelope evolution is still expected to be dominated by the release of gravitational (orbital) energy during the inspiral of the binary components.

Within this framework, the low-mass companion consumed by the V838 Mon primary had too low a luminosity to significantly contribute to the progenitor photometry, so its mass was calculated to account for the total power of the eruption. This mass had to be accreted on a timescale of at most a few days. If thermonuclear sources are excluded, as advocated above, V838 Mon’s eruption must have been primarily powered by the release of gravitational energy. To account for the short timescale of the event, the infalling mass likely required a relatively compact configuration, such as a star, rather than loosely bound circumstellar matter or a gas cloud. It was thus different from powerful protostellar eruptions, such as FU Ori-type events.

The stellar merger model originally proposed for V838 Mon has since been widely applied to other red novae. Proposed progenitor systems include mergers involving main-sequence stars, red giants, and yellow (super)giants. While V838 Mon represents a merger in a very young triple system, most red novae are thought to arise from mergers in more evolutionarily advanced binaries. This is especially true for post-MS stars that are rapidly expanding and are more prone to mass transfer and orbital instabilities leading to a merger. Yellow supergiants, which are objects in the Hertzsprung–Russell gap, are particularly well represented in the extragalactic sample of known red novae (Tranin et al. 2025) (Sect. 4.2). Mergers involving very compact stars, such as neutron stars, black holes, and their pairs, produce transients of very different characteristics than red novae (e.g., kilonovae) and have been widely discussed in the literature (Metzger 2019). They are omitted in this review.

Observations of red novae have often been linked to the long-standing problems of unstable mass transfer leading to common-envelope evolution (CEE) in binary stars (e.g., Paczynski 1976; Ivanova et al. 2013a, b, 2020). During this stage, orbital tightening combined with the loss of co-rotation between the donor’s envelope and the binary orbit causes the secondary star to move inside the expanded donor’s envelope. During this dynamical phase, the binary’s orbital energy is transferred into a partial or total ejection of the donor’s envelope (Webbink 1984; Ivanova et al. 2013a).

The formation of a common envelope is the culmination of a mass transfer episode, which can be initiated either by the expansion of the donor (primary star in this case) filling its Roche lobe, or the loss of orbital angular momentum from the binary, leading to the shrinkage of the orbit, or a combination of both. This loss of orbital angular momentum can occur via various mechanisms, such as magnetic braking, spin–orbit exchange between the stars (commonly referred to as the Darwin instability; Darwin 1879), mass loss from the binary system (Pols 2011; Henneco et al. 2024b) , or, in cases involving compact systems (not covered in this review), gravitational-wave emission. Finally, in the context of triple systems, there is also a possibility of a secular instability (Eggleton and Kiseleva-Eggleton 2001). None of the observed events so far has been associated with high-velocity collisions in globular clusters (i.e., not strictly in a long-lasting binary), but they too may manifest in the sky as red novae (van der Merwe et al. 2025).

In the context of red novae, the two dominant mechanisms proposed in the literature to trigger the initial orbital decay are Darwin instability and outer Roche lobe overflow. In the first case, in a tidally synchronized binary, the instability is triggered when the spin angular momentum of one star, typically an expanding post-MS component with a large moment of inertia, becomes equal to one-third of the total orbital angular momentum of the binary. Maintaining synchronization then requires angular momentum to be drawn from the orbit, leading to orbital decay and a runaway inspiral. Darwin instability is favored in systems with extreme mass ratios, typically q≲0.1. For example, for the red nova M31-LRN-2015, MacLeod et al. (2017) proposed rapid spin–orbit angular momentum exchange to explain the fast rise to peak luminosity observed in this transient.

In the second case of binaries experiencing increasingly high rates of mass transfer, material is likely to escape through the outer L2/L3 Lagrange points rather than being efficiently transferred (and accreted) between the stars. The outflowing gas carries away a substantial amount of orbital angular momentum, leading to efficient orbital tightening. For V1309 Sco, mass loss through the outer Lagrange points has been proposed to explain both the properties of the progenitor system and the rapid orbital decay observed during the pre-outburst phase (Tylenda et al. 2011a; Zhu et al. 2016; Pejcha 2014; Pejcha et al. 2017; MacLeod and Loeb 2020b). A combination of both scenarios is also plausible. For example, while Darwin instability was pointed out as the leading mechanism for the initial orbit tightening in V1309 Sco, this eventually caused L2/L3 overflow, which quickly accelerated the system to its final coalescence (Nandez et al. 2014).

At the formation of the common envelope, in addition to the possibly existing circumbinary medium, the companion becomes embedded in the gaseous outer layers of the expanded donor. Its orbital motion causes dynamical friction forces to arise from the gravitational interaction of the companion with its self-generated wake (Ostriker 1999; Kim and Kim 2007; Gagnier 2026). In addition to accelerating the orbital decay, the dissipation of orbital energy due to this drag force is expected to become a source of heating inside the envelope, which may cause radial acceleration from thermal or radiation pressure gradients, necessary for the successful acceleration of the envelope gas beyond the escape speed of the binary (Bronner et al. 2024, Karambelkar in prep.).

The dynamical (total or partial) ejection of the envelope causes the red nova outburst, and, depending on the binary’s properties, the final remnant can be a tighter binary composed of the primary’s core and the secondary star, or a stellar merger (Schneider 2025). Although ejecta mass estimates of red novae are usually well below the expected donor’s envelope mass—supporting the merger scenario (Tylenda et al. 2011a; Blagorodnova et al. 2021)—the literature is still inconclusive whether some of these events may instead result in a surviving binary (Karambelkar et al. 2023; Howitt et al. 2020; Twum et al. 2026). Indeed, some studies have attributed red novae to non-terminal common-envelope ejection events (e.g., Ivanova et al. 2013b) and grazing-envelope evolution (Soker 2015, 2016). In both scenarios, a binary avoids a merger either through efficient dispersal of the common envelope or by avoiding entering the CE phase whatsoever, owing to jetted outflows (e.g., Soker 2024).

Red nova outbursts are primarily powered by gravitational energy, with possible additional contributions from recombination energy (Ivanova et al. 2015; MacLeod et al. 2017; Metzger and Pejcha 2017; Reichardt et al. 2020; Henneco et al. 2024b; Chen and Ivanova 2024) and the prevalent role of shock heating (Metzger and Pejcha 2017; Kirilov et al. 2025). As discussed in Tylenda et al. (2024), for the case of the red nova CK Vul, a merger involving an RGB star and a helium white dwarf could also lead to a temporary activation of thermonuclear helium burning, contributing some 20% of the outburst luminosity. In certain cases, accreted mass may reach the primary’s hydrogen-depleted core and ignite nuclear burning, which can potentially contribute extra luminosity to the outburst and even lead to explosive thermonuclear runaway (Podsiadlowski et al. 2010).

## The outburst

### Light curves

**Fig. 3:**
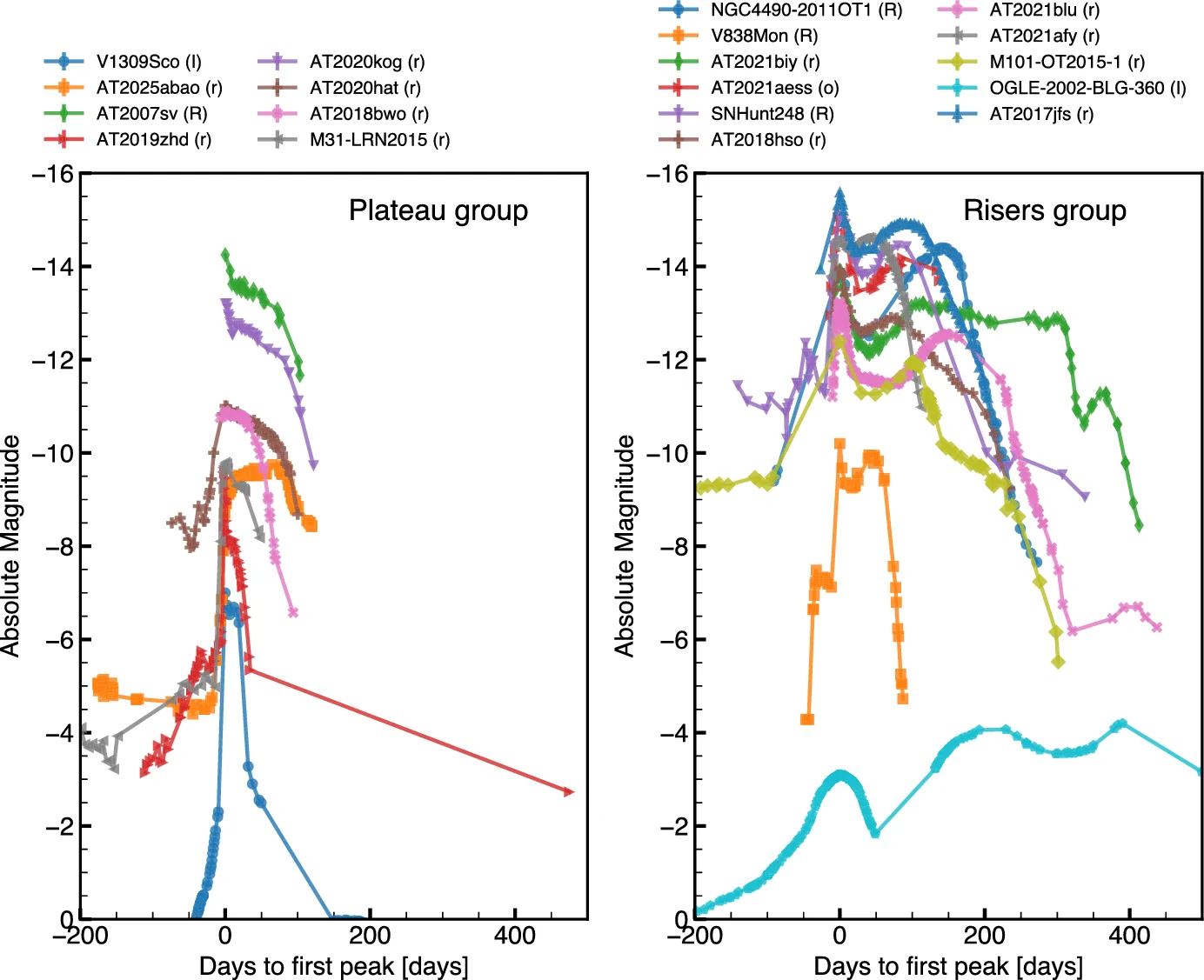
Light curves of red novae. The selected sample is divided into two groups. The ‘plateau group’ (left panel) and the ‘risers group’ (right panel). Each object is shown with a different marker and color, as specified in the legend. The light-curve photometric band is indicated in parentheses

Light curves and evolving SEDs of red novae are unique among astrophysical transients. Despite spanning a wide range of peak luminosities and light-curve shapes, they can be broadly grouped into two main morphological classes illustrated in Fig. 3: the ones showing a slow brightness evolution, which we call the ‘plateau group,’ and the ‘risers group’ showing a re-brightening to a second major peak. Most, but not all, members of the ‘plateau group’ show an initial ∼ 2-week-long peak, followed by a plateau lasting up to 100 days, and then a rapid decline. The members of the ‘risers group’ also show a fast initial peak, but in this case, the decline from the peak is followed by a second re-brightening, which sometimes reaches or even exceeds the brightness of the initial peak. The timescales of this second peak are longer, extending the overall duration of these transients from 80 to over 400 days if we consider a third or even a fourth peak in these light curves.

**Fig. 4:**
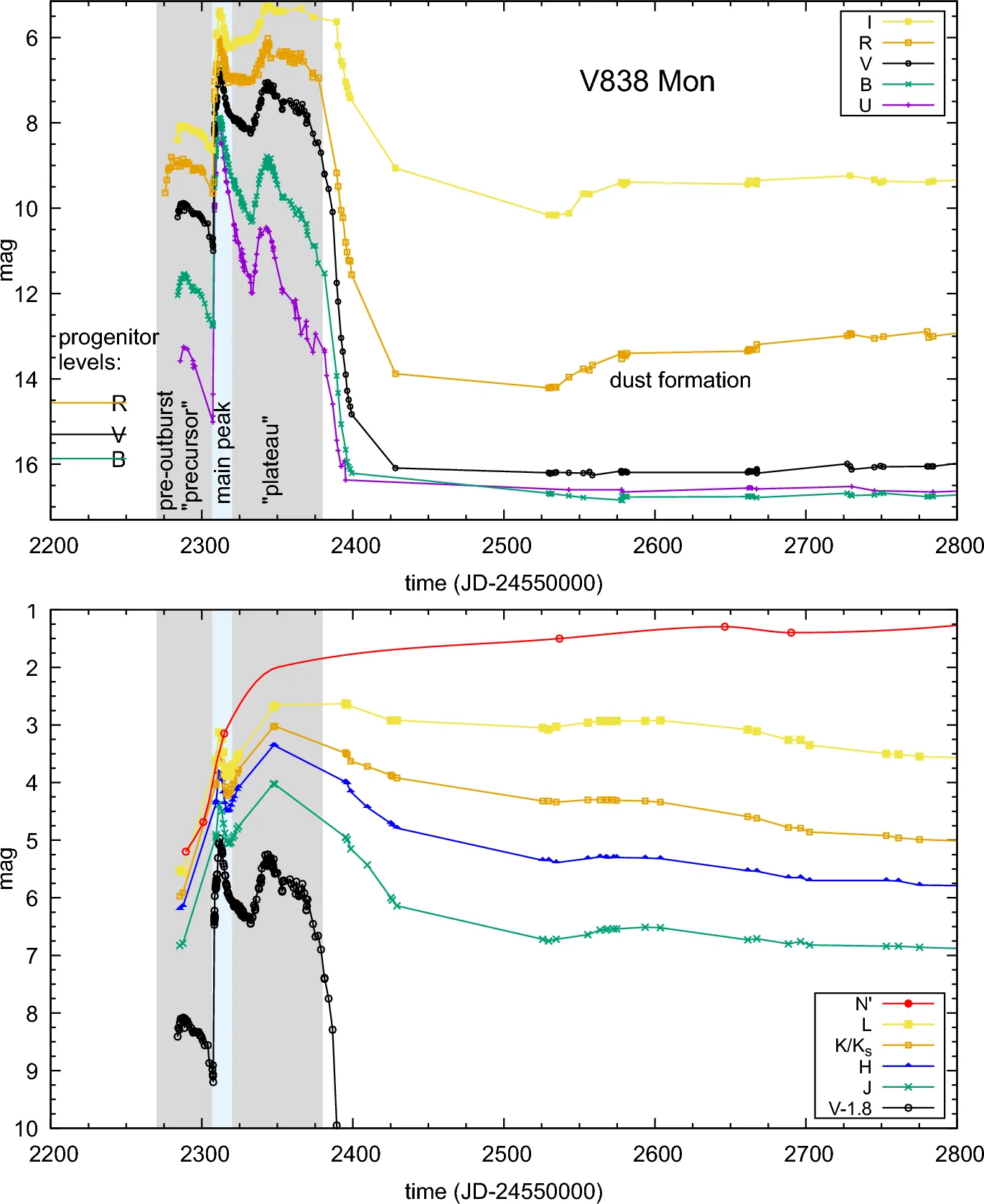
Multi-color light curves of V838 Mon in the optical (upper) and NIR (lower. The main phases of a typical red nova outburst discussed in the text are highlighted in gray and cyan. Observed data points are not corrected for extinction. The progenitor pre-outburst magnitudes for optical bands are shown on the left (the corresponding NIR progenitor magnitudes are well below the scale shown in the bottom panel). There are no measurements directly preceding the phase marked here as pre-outburst. The observations were compiled from Munari et al. (2002b), Goranskij et al. (2020), Banerjee and Ashok (2002), Loebman et al. (2015), Crause et al. (2003, 2005), Watson et al. (2006), Kamiński et al. (2021b), Chesneau et al. (2014), Woodward et al. (2021), Liimets et al. (2023). Solid lines simply connect the observed points except for $N^{\prime }$-band where we applied arbitrary interpolation

Given this morphological diversity, color evolution becomes a critical diagnostic to distinguish red novae from other transients. After the initial rise or peak, red novae quickly evolve to lower temperatures, which can be traced in their progressively reddened SEDs. Compared to other explosive transients like classical novae and SNe, the red nova ejecta cools down rapidly via adiabatic expansion. A well-observed example is V838 Mon, whose light curves across different bands are shown in Fig. 4. While the blue bands quickly fade after the first peak, emission in IR and mid-IR (MIR) bands increases with time. Red nova light curves typically evolve through the following phases:

***(I) Pre-outburst or precursor brightening***

Pre-outburst or precursor brightening can occur months to years before the main outburst and is characterized by a much lower amplitude, often 1–3 mag above the quiescent progenitor level, than the main outburst. Detecting this phase in archival data is challenging. The signal is often too faint or the transient position too uncertain for recovery in existing surveys. As a result, only the nearest red novae and those with brighter progenitors have been observed during this stage. The most notable example showing precursor brightening was V1309 Sco, where OGLE captured the steady increase in the eclipsing binary’s light curve, starting at about 5 years before its optical peak (see highlight D. in Fig. 1).

Among extragalactic systems, the best example of a precursor brightening is AT2025abao in M31 (WNTR23bzdiq; see Karambelkar et al. 2025), with archival photometry spanning 20 years before its outburst. The data reveal that the brightening in this system began about 10 years before the main outburst and the colors during this phase were comparable to those of the progenitor star. Other red novae with detected precursor emission are SNHunt248, M101-OT2015-1, M31-2015-LRN, and AT2019zhd (Kankare et al. 2015; Blagorodnova et al. 2017, 2020; Pastorello et al. 2021a). They show a 1–5 mag brightening starting ≲5 years before the outburst.

Notably, precursors often end with a short-lived dimming episode, hereafter referred to as ‘the dip,’ occurring just before the fast rise. For instance, it was observed in V1309 Sco (see first half of the highlight B. in Fig. 1), where it was assigned to increased obscuration from a dusty disk. Due to the limited availability of follow-up observations before the main peak, color evolution during the dip phase has been constrained for only a small number of objects. For V838 Mon and AT2025abao, the observed colors show only a small reddening compared to the rest of the precursor phase (≤0.3 mag; Reguitti et al. 2026b), arguing against significant dust obscuration.

***(II) The main outburst***

The main outburst begins with a rapid rise in brightness, typically at a rate of 0.4–1 mag $\hbox {day}^{-1}$. For objects without a detected precursor, this stage marks the first identification of the system as a transient in time-domain surveys. During this phase, light curves reach their highest temperatures, typically in the range 4000–9000 K, and it is therefore sometimes referred to as the ‘blue peak.’ A few transients reached even higher temperatures, of ∼11,000 K, resulting in detected ultraviolet (UV) emission (AT2020kog and AT2021biy; Pastorello et al. 2021b; Cai et al. 2022b). However, among the plateau group, some red novae seem to skip the blue peak overall, directly transitioning to the next phase (e.g., AT2020hat and AT2025abao). In many cases, the main peak corresponds to the maximum light of the event, especially at shorter visual wavelengths. The post-peak decline is comparable to, but generally slower than the rise, and is associated with a rapid cooling of the photosphere.

***(III) Plateau or red peak***

**Fig. 5:**
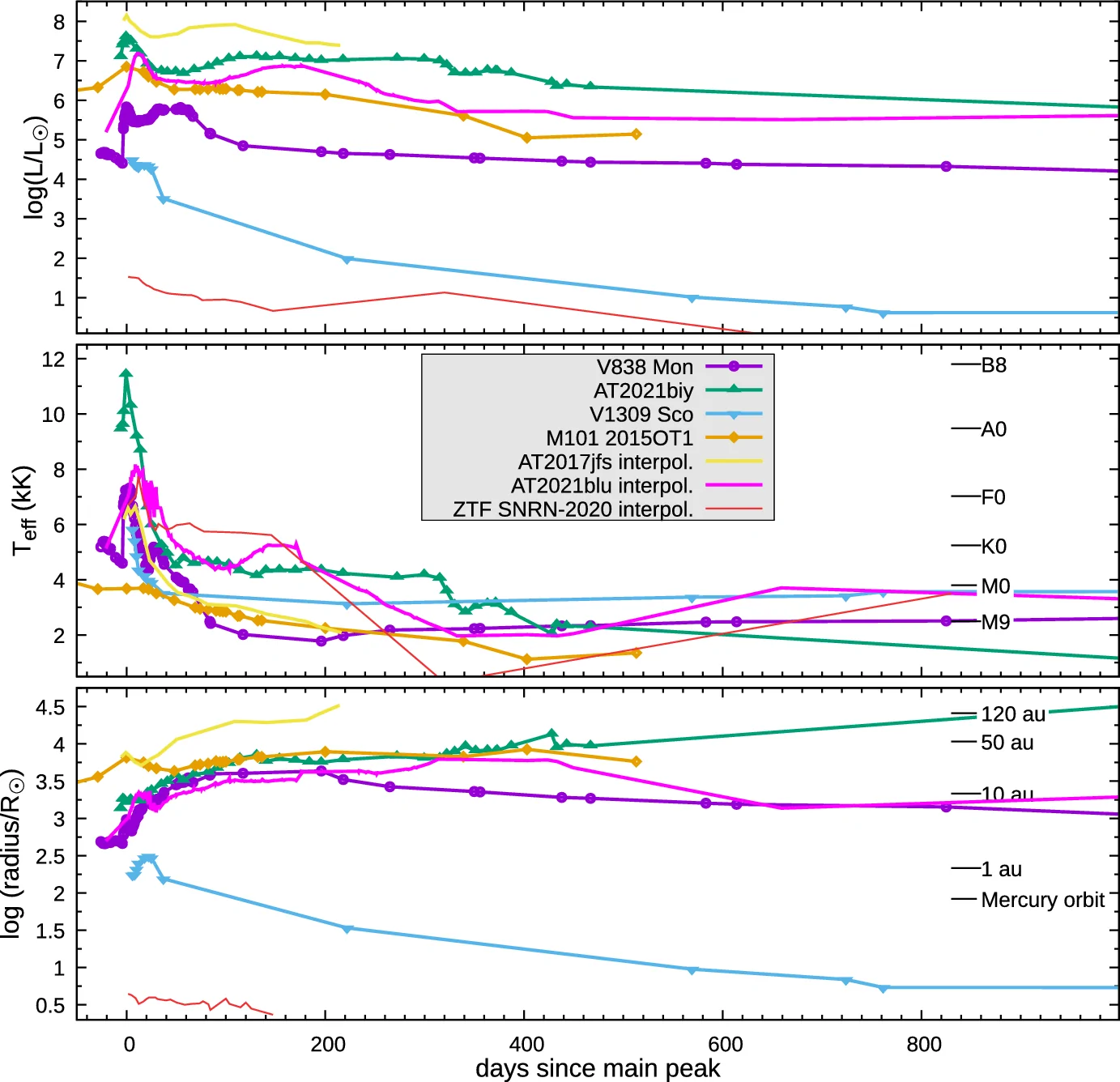
Evolution of basic (pseudo-)photospheric parameters of red novae. The shown parameters were derived mainly from blackbody fits to the observed SEDs. Data are from Tylenda (2005); Cai et al. (2022b); Tylenda et al. (2011a); Tylenda and Kamiński (2016); Blagorodnova et al. (2017); Pastorello et al. (2019a); Karambelkar et al. (2026); Wavasseur et al. (2026), De et al. (2023); Lau et al. (2025); the last 3 curves show interpolation between data points for better clarity. See the original papers for error bars which are not included here. Distance to V1309 Sco is uncertain

Depending on the morphological class, the fast decline from the main peak may either flatten into a slower plateau phase or give way to a secondary re-brightening to a similar or modestly fainter maximum (Δm<1), initiating the ‘red peak’. The plateau phase may comprise a series of small-amplitude peaks and dips rather than a smooth decline. Colors at this stage are redder than at earlier phases (see Fig. 5), but for some objects from the risers group, a clear second increase in temperature is observed (UGC12307-2013OT1 and SNhunt248; Pastorello et al. 2019b). Because of its longer duration, the plateau phase often dominates the total radiated energy, and for some red novae, maximum light in the red optical and the IR bands is reached during this stage (e.g., BLG360 or V838 Mon observed in $I_C$, or NGC4490-2011OT1). Additionally, in several red novae classified as ‘risers’, an additional late-time re-brightening is seen, producing a lower-luminosity third peak (e.g., BLG360, SNHunt248, AT2021blu) or, in extreme cases, even a fourth peak, as in AT2021biy.

***(IV) The decay***

During the decay phase, the plateau (or red peak) ends, and the light curve starts to decline much faster (around 0.1–0.2 mag $\hbox {day}^{-1}$). Optical to MIR photometry taken at this stage shows SEDs with clear IR excesses, which have been associated with signatures of dust at temperatures of 100–1500 K (Tylenda et al. 2011a; Blagorodnova et al. 2020; Wavasseur et al. 2026, Gomez-Muñoz in prep.). Although the post-outburst bolometric luminosity remains above the progenitor level for decades (Kamiński et al. 2021b; Forés-Toribio and Kochanek 2026), the post-plateau evolution at visual wavelengths often falls well below the progenitor level due to extinction from freshly condensed dust. The lower temperatures in the remnant and its surrounding dense gas contribute to the nucleation of dust grains, which reprocess the central hotter emission of the coalesced star into longer wavelengths. Contrary to the fast decay of optical light, the IR flux for some red novae keeps increasing for months to years after the end of the main optical peak (Karambelkar et al. 2026; Reguitti et al. 2026a) (see V838 Mon in Fig. 4) and perhaps some red novae can only be observed through this late IR signature without an optical counterpart (Jencson et al. 2019b). Late post-outburst photometric evolution and relaxation phase of red nova remnants is described in Sect. 6.

The duration of each phase (I–IV) varies widely among red novae (see Fig. 3), possibly due to different progenitor masses, different evolutionary stages of the progenitor stars, different binary configurations at the onset of the event (see Sect. 5.1), and different orbital instability mechanisms at play. For example, in the relatively fast red nova V838 Mon in the ‘risers group’, the precursor phase lasted ≳35 days, the main peak lasted 10 days, and the plateau stretched for over 70 days. For comparison, M31-LRN-2015 in the ‘plateau group’ and with similar peak brightness was even faster, with a plateau length of only ∼ 40 days. For the very slow red nova BLG360, the timescales were 470, 240, and > 660 days for precursor, main peak, and plateau, respectively, illustrating the diversity of red novae light curves.

Despite their heterogeneity, the characteristic multi-peak, quickly reddening light curves remain an important tool for identifying new transients of this type and are often readily distinguishable from other classes of outbursts. For example, in classical novae, the ejecta expansion leads to increasing effective temperature as deeper, hotter layers become visible (a small subsample exhibits secondary variations or ‘jitters’ in their light curves). This produces a systematic evolution toward bluer colors with time, which is the opposite of what happens in red novae. Gap transients such as ILOTs do not have a plateau and show a continuous decline from the peak (Pastorello and Fraser 2019; Jencson et al. 2019a; Valerin et al. 2025a) with little color evolution. The LBV outbursts are characterized by longer timescales than those of red novae and possibly display previous activity (Pastorello et al. 2010; Beasor et al. 2026). It is, however, still often the case that red nova light curves have been confused with other types of transients or even microlensing events (see notes in Table 1 and most recent reclassifications in Reguitti et al. 2026a). Due to their unique circumstances, some historic red novae, such as CK Vul or BLG360, were classified solely based on their optical or IR light curves and SED evolution, as no spectra were taken during their outbursts.

Multi-wavelength light curves of red novae have often been used to compare the colors of the transient with low-gravity stars to estimate their spectral classification. In addition, studies have also built multi-epoch SEDs to trace the changes in the physical parameters of the expanding object, most often under the assumption of a black body surrounded by a spherical dust shell. This approach provides estimates of semi-bolometric luminosity and parameters such as effective temperature, photospheric radius, expansion velocity, and dust properties (e.g., Tylenda 2005; Pastorello et al. 2019b; Blagorodnova et al. 2020; Reguitti et al. 2026a) (see Fig. 5). Such analyses commonly assume spherical symmetry and are based on sparse photometric data, rarely extending beyond the near-IR (NIR). This can lead to an underestimation of dust-related IR emission and makes the results especially incomplete at late times, when the luminosity is increasingly dominated by emission at longer wavelengths.

The growing sample of well-covered light curves has shown a consistent picture of their properties during the outburst. The main peak is characterized by the highest effective temperatures, typically 7000–12 000 K. The corresponding effective radii are in the range of 100–8000 $\textrm{R}_{\odot }$, in extreme cases reaching ≈40 au. During the plateau phase, the radii grow by a factor of a few, sometimes reaching even 12 000 $\textrm{R}_{\odot }$, and the effective temperature drops to 4000–4500 K. After the decline phase (years after the main outburst), in some objects it was observed that the expanding material becomes progressively geometrically thinner, resulting in the shrinkage of the effective radius; the thinning eventually exposes the inner coalesced core, which is a bloated star of spectral type M with effective temperature of 2000–3500 K (e.g., Tylenda et al. 2009). Nevertheless, the majority of central stars remain obscured for much longer due to a thicker dust ejecta (Steinmetz et al. 2024; Karambelkar et al. 2026). Their effective radii grow at a rate consistent with the observed gas expansion velocity known from spectroscopy. So far, optically thick ejecta with effective radii as large as ≈ 200 au have been reported (Karambelkar et al. 2026; Mauerhan et al. 2018). The size evolution in red nova outbursts has been investigated mainly through indirect methods because direct size measurements, e.g., using IR interferometry, would be challenging even for the closest objects identified to date (cf. Mobeen et al. 2024). We also caution here that in late phases of the evolution of some red novae, their photometric fluxes in the optical to NIR are grossly dominated by emission features of atoms and molecules (e.g., Banerjee and Ashok 2004; Kamiński et al. 2015a), not continuum, and the type of analysis we discussed here leads to erroneous results (as discussed in Steinmetz et al. 2024).

Ultraviolet observations of red novae are sparse, during outburst (Rauch et al. 2002; Smith et al. 2016; Pastorello et al. 2023) and in the post-outburst phase (Mauerhan et al. 2018). No red nova in outburst has been detected in X-ray or low-frequency radio emission, not even the nearest bright sources (Antonini et al. 2010). Under normal circumstances and for non-compact star mergers, such emission is not expected (but see Soker and Tylenda 2007), as thermal emission at these energy regimes must be weak. To date, red novae outbursts have not been detected in gravitational waves (but see Sect. 7)

Light echoes are occasionally associated with red nova outbursts, but such cases are rare, as the appearance of an extended light echo requires that the eruption take place near dusty interstellar medium, and so it is more likely for young or very massive progenitors. Up to date, V838 Mon light echo remains the iconic example of this phenomenon observed both in scattered optical light (Bond et al. 2003) and in thermal IR (Banerjee et al. 2006). Another possible light echo was postulated for N4490-OT2011 (Reguitti et al. 2026a), who proposed this scenario to explain the lack of MIR color evolution in the remnant source during the first 6 years after the red nova event.

### Spectral characteristics

**Fig. 6:**
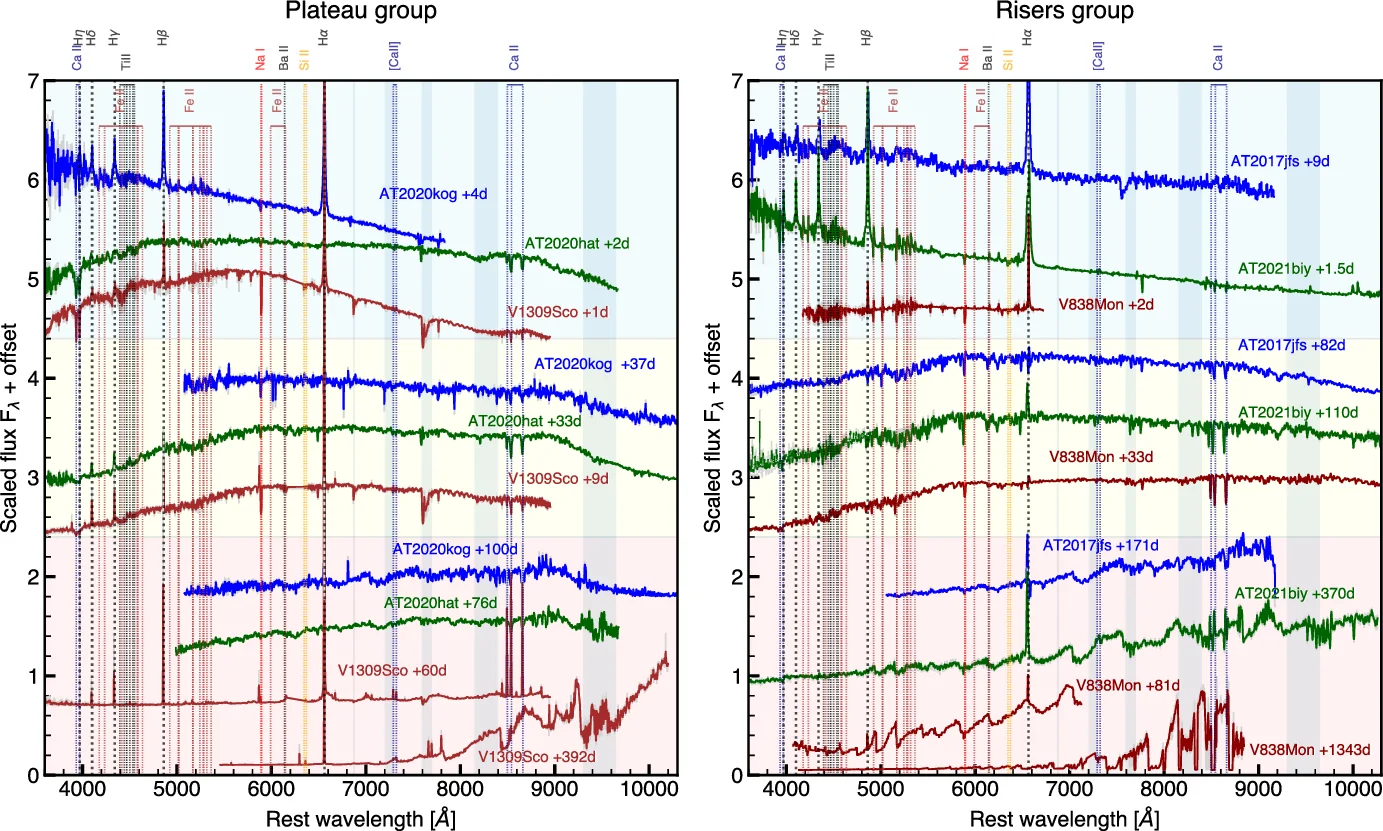
Spectral evolution for the ‘plateau group’ (left) and ‘risers group’ (right) of red novae (cf. Figure 3). The reference epoch is given in reference to the date of the optical peak. Spectra acquired around the main (blue) peak have a blue background; spectra taken during the plateau (red peak) phase have a yellow background; spectra taken during the decay phase have a red background. Each group comprises three objects of different peak brightness. The data have been obtained from Pastorello et al. (2021b, AT2020kog and AT2020hat), Pastorello et al. (2019a, AT2017jfs), Mason et al. (2010, V1309Sco), Cai et al. (2022b, AT2021biy), and Bond et al. (2003); Kamiński et al. (2009); Munari et al. (2002a, V838Mon). The spectra have been corrected for Galactic extinction and redshift

The spectral evolution of red novae discussed in this section mostly focuses on optical wavelengths, which have been used extensively to identify red novae. Grouped by their light-curve morphology, a sample of optical spectra is shown in Fig. 6 and corresponds to high-, medium-, and low-luminosity events shown in Fig. 3. The background color indicates the phases during which the spectra were taken and which are discussed below.

***(I) Pre-outburst***

Because red nova precursors are most often identified retrospectively in archival data, spectroscopic observations during this phase are rare. So far, spectroscopic characterization during precursor brightening only exists for AT2025abao (Karambelkar et al. 2025), which was identified as an object potentially undergoing common-envelope ejection from an AGB star. The earliest spectra were taken at optical and NIR wavelengths ∼ 2 year before the outburst discovery in October 2025. The authors noted a discrepancy in the typing of the stellar type. While the optical emission matched a hotter M1-type star, the identified features of TiO, CO molecular absorption, as well as water vapor, better matched a cooler M8–M9-type star, resulting in a mixed temperature spectrum which persisted during the whole observational period until January 2025. This phenomenon was previously observed in other red novae, such as V838 Mon and the planet-merger ZTF-SLRN-2020. The emission pattern was consistent with a hotter star surrounded by cooler dense shells (Lynch et al. 2004, 2007). While the shorter wavelengths originated deeper in the envelope, longer wavelengths traced the emission from the cooler, outer layers. This is commonly observed in YSOs.

***(II) The main outburst***

**Fig. 7:**
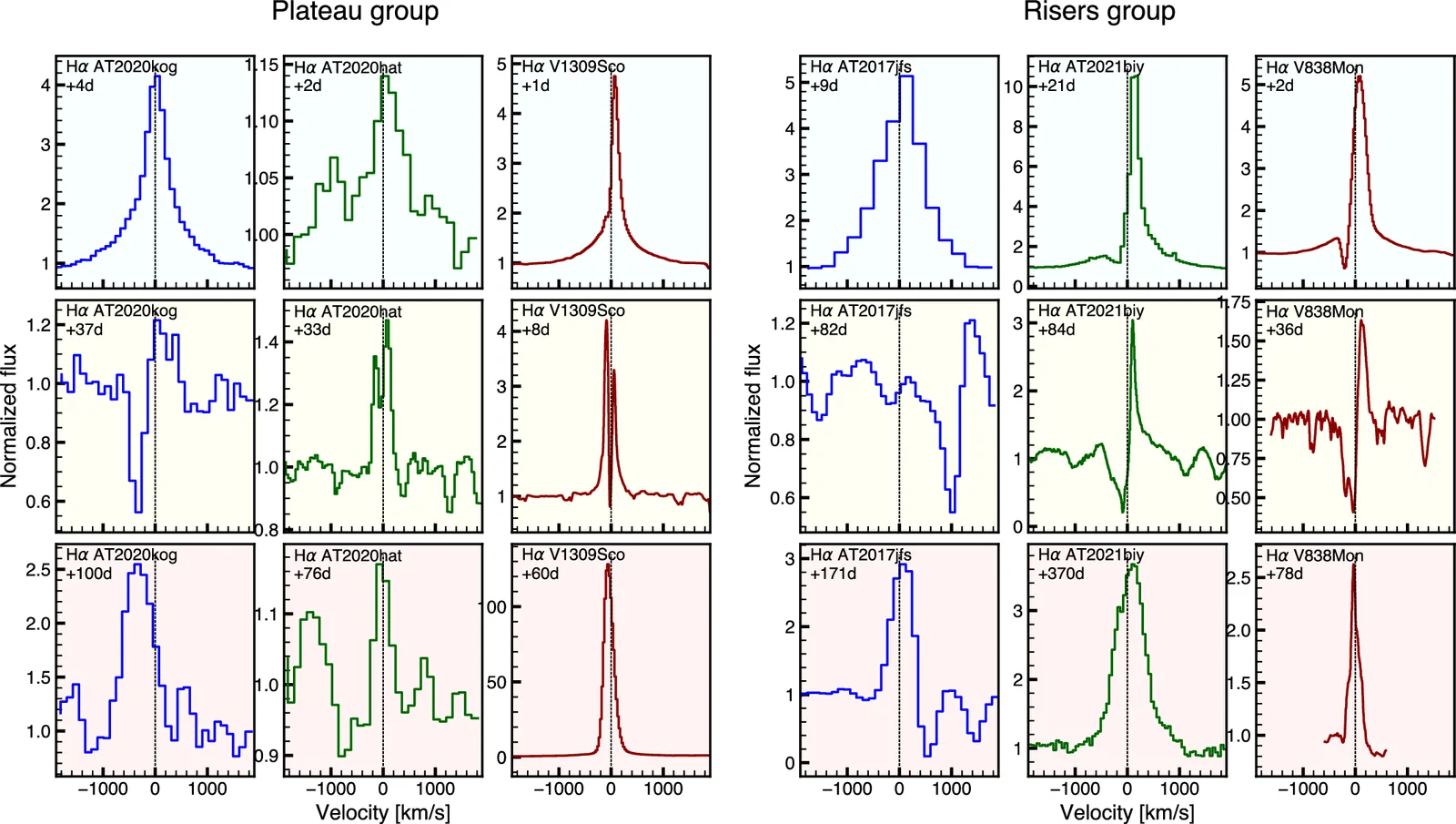
Spectral evolution of Hα for objects in Fig. 6. Blue, yellow, and red backgrounds indicate the respective phases as in Fig. 6 (tom to bottom): blue peak, plateau/red peak, and decay. The names of the objects and their epoch relative to the date of the optical main peak are shown in each panel. The spectra are scaled to the continuum and smoothed with a Gaussian 0.3 Å filter for display purposes. Data obtained from Pastorello et al. (2021b, AT2020kog and AT2020hat), Pastorello et al. (2019a, AT2017jfs), Mason et al. (2010, V1309 Sco), Cai et al. (2022b, AT2021biy) and also Wavasseur et al. in prep., Bond et al. (2003); Munari et al. (2007); Wisniewski et al. (2003, V838 Mon)

During the main peak, red novae show a hot continuum and strong, narrow^1^ (< 2000 $\text {km s}^{-1}$; typically much less) Balmer emission, reminiscent of other eruptive transients such as SNe IIn or LBV eruptions. At this stage, the peak luminosity provides a useful first indicator for classifying an event as a supernova, but it is not sufficient on its own, leading to frequent misclassifications. As shown in the Hα spectra in Fig. 7, during the main peak, the hydrogen lines display Lorentzian profiles with extended (> 1000 $\text {km s}^{-1}$) electron-scattered wings (cf. Wisniewski et al. 2003). For some luminous events (e.g., AT2021blu and AT2021afy, Pastorello et al. 2023), the reported Balmer Hα to Hβ flux ratio was ≈2, somewhat smaller than expected from the nominal value of ≃ 2.85 corresponding to Case B recombination of gas at 10 000 K (Osterbrock 1989). This effect, observed in cataclysmic variables and SNe IIn (Elitzur et al. 1983; Chugai et al. 2004), suggests that either the Balmer lines become optically thick and are reabsorbed, or that internal collisional processes enhance the population of the upper levels of the Balmer transitions, effectively boosting Hβ emission and flattening the Balmer decrement toward smaller ratios, or both. The hydrogen emission indicates that dense ($>10^8$ $\hbox {cm}^{-3}$) matter undergoes ionization and recombination in regions above the expanding photosphere.

The Hα profile often has a narrow absorption component, overlapping with the blue wing of the much stronger emission component (e.g., Martini et al. 1999; Wisniewski et al. 2003; Mason et al. 2010), and it may quickly change location or temporarily disappear (e.g., Cai et al. 2022b; Pastorello et al. 2021b). The absorption may be interpreted as a signature of the circumstellar material that is on the line of sight but located far from the eruption so that it is not significantly affected by the ionization front. At least in some cases, it may be the material lost by the system long before the red nova outburst (Martini et al. 1999; Mason and Shore 2022). Several objects of the ‘risers’ group may also show a broad absorption component, indicative of an ongoing fast outflow, forming a classical P Cygni-type profile. In terms of absolute fluxes and compared to the local continuum, emission in Hα is most intense during the main outburst and fades away during the plateau phase.

Spectra obtained with a sufficient spectral resolution during the outburst display absorption lines for elements other than hydrogen. These are often interpreted as features arising in the photosphere of the optically thick expanding ejecta. The absorption spectrum is thus used for spectral typing, complementary to temperature constraints obtained with SED analysis (cf. Sect. 3.1). Examples of features often observed this way are the H&K doublet and IR triplet of Ca ii, visual lines of Ba ii, Sc ii, Ti ii, and, most commonly, Fe ii (see line identification in Fig. 6). This pseudo-photospheric or indeed photospheric component matches spectral types from A–G in the early phases, and M supergiants (luminosity class I) in the latest phases of the eruption. In some extreme cases, for instance, during the main peak of NGC4490-2011OT1, the effective temperature read from spectra was as high as 18 000 K (corresponding to spectral type B4, see Pastorello et al. 2019b), but such hot photospheres are rare among red novae, especially among those with low-mass progenitors. It should be kept in mind that strict or accurate spectral typing is not possible for these eruptive objects, as the spectrum does not arise in hydrodynamic or thermodynamic equilibrium and line profiles are contaminated by non-photospheric components. Regardless, the evolution through spectral types is often very rapid, especially in the final phases of the optical outburst, when a change of one spectral type over one night is not uncommon.

Effective temperatures of 7000–11,000 K near the bolometric peak (or at the main peak) inferred from spectral typing are relevant for assessing the role of recombination energy in powering the outburst. The role of the associated release of internal energy on the system evolution is hotly debated in the literature (cf. Sect. 3.5) and may depend on the nature of the binary undergoing the red nova event (cf. Reichardt et al. 2020).

Emission in lines other than in the Balmer series is comparatively weak during the main peak, but not unmet (e.g., Na i and Ba ii lines in V838 Mon, Iijima and Della Valle 2002; Fe ii, Ti ii, Ca ii and O i in NGC4490-2011OT1, Pastorello et al. 2019b; Na i, Fe i–ii, Mg i, and [O i] in V4332 Sgr, Martini et al. 1999). The permitted lines generally show a P-Cygni profile, with lower expansion velocities than the ones estimated from the Hα line. In contrast to some other gap transients, the firmly identified red novae do not show [Ca ii] $\lambda \lambda$7291,7323 during the main peak, but the lines have been detected in the plateau phase (e.g., Mason et al. 2010).

***(III) Plateau or red peak***

After the main peak, the continuum reddens considerably. Balmer emission lines also dramatically weaken, with low or undetectable Hβ and only marginal Hα. Moderate-resolution spectra of the Hα region also reveal the presence of low-velocity narrow absorption lines, likely associated with a cooler shell or disk surrounding the recent merger event. For plateau spectra, observers often report ‘a forest’ of metal lines, especially pronounced near 5000 Å. During this phase, metallic lines previously showing P-Cygni profiles now transition to pure absorption, and the Ca ii triplet becomes stronger. In some objects, Hα next regains its flux in the later phases of the plateau (see Fig. 7). Late-time spectra, taken hundreds of days after the main peak, show that the Balmer emission can eventually disappear when the spectrum turns into an M-type star.

***(IV) Decay***

In the latest phases of the fast fading from the plateau, the absorption spectrum shifts from yellow toward progressively more red photospheres, eventually displaying a spectrum mimicking M-type giants and supergiants. Most characteristic for this phase are absorption bands of TiO and VO (the latter only in the red part of the optical spectra). The latest spectral type reported in this phase is later than M10 (Evans et al. 2003), but it should be noted that these dimming photospheres especially are embedded in cool circumstellar molecular gas, which strongly affects the absorption features used for spectral classification. This may lead to the assignment of spectral types later than actually characterizing the stellar photosphere. While the molecular bands at the end of the outburst of a new transient are generally a smoking gun of a red nova, there have been cases when objects miss any detectable features (e.g., BLG360; Steinmetz et al. 2025) or the object was still too hot for the molecular bands to be apparent in the spectra (e.g., NGC4490-2011OT1; Smith et al. 2016). In the plateau and later phases, the photospheric absorption spectrum and that of circumstellar molecules are combined with the emission spectrum of hydrogen and of species Na i, K i, [Ca i], [Ca ii], Ca ii, [O i], and Mn i (e.g., Martini et al. 1999).

As described in more detail in Sect. 6, early post-outburst spectra of red novae indicate an environment of low temperatures. When the stellar-remnant photosphere is observed, which is a rare case even for the Galactic red novae, it shows a giant or supergiant photosphere of spectral type M (e.g., Kamiński and Tylenda 2013). The post-outburst system is best studied through spectral features of the circumstellar medium, often exclusively through emission lines, including gas and dust probes at IR and submillimeter wavelengths. Some spectral appearances of red nova remnants can mimic young stellar objects (YSOs; for instance, those with molecular emission bands, Guo et al. 2024). A surprisingly common spectral feature in the early post-outburst phases of red novae is the presence of molecular bands of AlO (Banerjee et al. 2015). Bands of CrO have been observed exclusively in these remnants (Kamiński et al. 2015a; Steinmetz et al. 2024).

***Kinematics over the entire outburst***

Emission and absorption lines have been used to derive the merger ejecta velocities. However, those measured from the widths of the hydrogen recombination lines in emission are almost always higher (even 2000 $\text {km s}^{-1}$ FWHM) than those measured with other probes. Scattering of electrons in the recombining plasma can often broaden the lines and give them a Lorentzian shape. This is true especially in the early parts of the main peak, when the ionization degree is highest (see top row of Fig. 7). Therefore, outflow velocities derived from other lines, especially from the optical Na i, resonance doublet, are generally a more reliable measure of the ejecta kinematics (if the interstellar component can be accounted for). These lines trace very well the cool matter that is associated with all red novae. The FWHM velocities measured this way in spectra of red novae in outburst are typically 100–500 $\text {km s}^{-1}$ and correlate with other properties of the system (see Sect. 5.1). The continuum definition is often a significant source of uncertainty and can affect the measured velocities.

The peak or central positions of emission and absorption lines can shift over the duration of the outburst, but it is unclear what dominant factor is causing it. Asymmetric and variable line profiles, when unresolved with poor spectral resolution, may be a plausible explanation. Studies of the red nova remnants have demonstrated that the central line velocities observed during the outburst are generally not a good measure of the systemic velocity (Kamiński et al. 2018a). Moreover, the line widths change over the course of the outburst, and those indicate real changes in the ejecta velocities. The highest ejecta velocities are found near maximum brightness and often exceed the escape velocities of the presumed progenitors. Matter ejected in later phases of the outburst is slower. These variable ejecta velocities are also well evidenced in the kinematic structure of the remnants observed years, decades, and centuries after the outburst.

### Non-optical spectra

Red novae in outburst have been mainly studied at optical wavelengths, and little is known about their spectral characteristics at shorter (UV) and longer (MIR–FIR–submm) wavelengths during the eruption. Red and NIR spectra of V1309 Sco acquired during its main peak showed Fe ii emission lines and the Paschen and Brackett series mostly in absorption, except for Hβ to γ Paschen transitions, which showed only emission (Rudy et al. 2025). Later in the main peak, absorption bands of CO and $\hbox {H}_2$O could have been present (Rudy et al. 2008a, b, 2025). The 2.3–2.4 μm bands of CO were observed throughout the entire outburst of V838 Mon (Geballe et al. 2002) as well in late-time spectra for some extragalactic red novae (Blagorodnova et al. 2021; Cai et al. 2022b; Karambelkar et al. 2023). As soon as dust condensation occurs (plateau or later), IR dust features show up. Most characteristically for the galactic and extragalactic red novae, a few years after the main outburst, a broad ‘10 μm’ feature is observed in absorption (Nicholls et al. 2013; Karambelkar et al. 2026). The feature is caused by freshly condensed dust consisting of silicates and, less often, of alumina or iron grains (e.g., Banerjee et al. 2015; Steinmetz et al. 2025).

### Polarimetry and spectropolarimetry

Spectropolarimetry experiments are rare for red novae, but a few observations have been performed during or shortly after the outburst (Desidera et al. 2004; Wisniewski et al. 2003; Cai et al. 2022b) and several years later, during the post-outburst phases (Kamiński and Tylenda 2013; Zain Mobeen et al. 2025). When corrected for interstellar extinction, the continuum polarization of the luminous red novae is very low, at a level of a few %. In the case of V838 Mon, polarimetric observations revealed that the main luminosity peak coincided with the maximum polarization observed of this object (intrinsic polarization of 0.7–1.0%), indicating that the emitting region was not spherical (Wisniewski et al. 2003; Desidera et al. 2004). At shorter wavelengths, the object appeared to be more polarized, which suggested the existence of additional mechanisms besides Thomson scattering. Freshly formed dust, or older dust predating the explosion, could also contribute to scattering at this low level. However, the changes in polarization in V838 Mon were just of the order of days, which were at odds with longer dust formation timescales. In simple axisymmetric scattering models, continuum polarization of order 1% may correspond to a departure from spherical symmetry of order tens of percent (cf. Hoeflich et al. 1996). However, the precise conversion is highly model-dependent (e.g., Dessart et al. 2021) and cannot be uniquely determined for V838 Mon because its inclination, optical depth, scattering mechanism, and dilution by unpolarized light were poorly constrained.

Studies of polarization in spectral features are even more scant. During and shortly after its outburst, V838 Mon showed only small depolarization effects across Hα. Due to the very modest signal-to-noise ratio (S/N), these observations were not very informative. For the low-mass red nova remnant V4332 Sgr, however, continuum polarization as high as 17% was observed and interpreted as scattering off the dusty disk and bipolar outflows surrounding the star (Kamiński and Tylenda 2013, see also Sect. 6.2.1). The presence of pronounced depolarization in atomic lines and molecular bands revealed a stratified circumstellar structure, in which the gas producing fluorescent emission is decoupled from the asymmetric continuum-scattering geometry. Such observations can thus be used to investigate the structure of the objects in outburst or during the remnant phase. A major limitation is that such observations require both high S/N and high spectral resolution; as a result, detailed spectropolarimetric studies are restricted to the brightest events and have proven challenging even for the most luminous red novae in the Milky Way.

### Interpretations of the outbursts

The interpretation of the observed outbursts, especially the multiple peaks in the light curves, is still debated. In the early days of the red nova research, it was proposed that coalescence occurs through a series of grazing collisions, with each periastron passage producing a separate peak in the light curve (cf. Tylenda and Soker 2006; Tylenda et al. 2013). Another hypothesis linked each peak to the engulfment of a planet (Retter et al. 2006). However, these early hypotheses did not receive much support in recent studies. In particular, it would be difficult to explain why we often observe 2–3 peaks in systems of very different progenitor types and orbital parameters. Simulations of common-envelope evolution often relate to the red nova mergers but encompass a much broader range of phenomena than red novae; we therefore refer the reader to dedicated reviews (Ivanova et al. 2013a, 2020; Röpke and De Marco 2023; Schneider 2025), and focus here on current efforts to reproduce the observed light curves of red novae, specifically.

Recent studies encompass 1D (Lipunov et al. 2017; Chen and Ivanova 2024; Metzger and Pejcha 2017; Matsumoto and Metzger 2022; Chen 2026), 2D (Kirilov et al. 2025), and 3D (e.g., Hatfull et al. 2021; Mu et al. 2026) (radiation-) hydrodynamical models, with some success in explaining the observations. A major advance was the inclusion of radiation transport and radiation hydrodynamics (Chen and Ivanova 2024; Hatfull et al. 2021; Kirilov et al. 2025), but fully self-consistent models are still missing. Below, we briefly review a selection of recent ideas proposed to explain the observed characteristics of red nova outbursts. However, it may still be premature to generalize the physical mechanisms, as factors such as viewing angle, orbital configuration, mass ratio, and evolutionary stage can introduce significant diversity, and both observations and models remain limited.

***Precursor brightening***

Only the closest and brightest objects were confidently detected during the gradual precursor rise months to years before the main outburst, with widely varying timescales and amplitudes (see Table 1; Sect. 3.1). For example, in M31-LRN-2015, the precursor began tens of orbits before peak (MacLeod et al. 2017), whereas in V1309 Sco, it started ∼1300 orbits earlier (Tylenda et al. 2011a; Pejcha 2014; Pejcha et al. 2017). For the more extended AGB progenitor of AT2025abao, the precursor emission initiated ∼10 years before its transient event (Karambelkar et al. 2025). Observations of the light curve of V1309 Sco show that at the end of the precursor phase the eclipsing binary modulation disappears (see highlight B. in Fig. 1), i.e., when the binary becomes embedded in the optically thick material. Some works linked it to the onset of the common envelope (e.g., Pastorello et al. 2019b).

While the precursor rise has rarely been modeled in radiation-hydrodynamic studies of the light curves, there is generally a consensus (but see discussion in Nandez et al. 2014) that this phase is related to a runaway buildup of circumbinary matter, mainly via the outer Lagrange L2 point. Runaway mass loss from the system accelerates orbital decay, ultimately driving the binary toward merger and producing the characteristic ‘concave-up’ light-curve evolution observed in precursor emission (Pejcha et al. 2017; MacLeod et al. 2018b; MacLeod and Loeb 2020b). The evolution of binaries experiencing extreme mass-transfer rates is an active area of research, providing new constraints on the pre-merger (or pre-supernova) mass-loss phase through detailed treatment of momentum exchange and the formation and structure of mass outflows (e.g., Lu et al. 2023; Scherbak et al. 2025). Here, we mostly focus on the connection with red nova events.

Pejcha et al. (2017) (see also Pejcha et al. 2016a, b) specifically investigated the precursor evolution of red novae, with a primary focus on reproducing the observed properties of the V1309 Sco progenitor system. They demonstrated that the rise in brightness was due to collisions and shocks within dense material accumulated by the binary in the orbital plane. In later phases of the precursor (e.g., near 2007 in Fig. 1), the shock luminosity approaches the binary’s luminosity itself, fueled by increasing disk mass and higher temperature of the gas feeding the outflow. MacLeod et al. (2017) proposed that the precursor peak is related to tidal heating of the primary, which is conceptually consistent with the ‘velocity shear’ model of Pejcha et al. (2017). The mass accumulated during the precursor phase may be comparable to that ejected during the dynamical interaction, and thus strongly influences later evolution, calling for more observational identifications of precursors. Observationally, this picture is consistent with the bipolar morphologies and disks detected in low-mass mergers like V1309 Sco and V4332 Sgr (Kamiński et al. 2018a; Steinmetz et al. 2024).

Finally, such prolonged brightening may also be qualitatively consistent with heating driven by hydrodynamic drag within the common envelope. As drag extracts orbital energy and angular momentum from the binary orbit and transfers them to the surrounding envelope, part of the released orbital energy is deposited as heat, contributing to an enhanced luminosity (Bronner et al. 2024; Bhattacharyya et al. 2026, Karambelkar in prep.).

***Dip***

The dip may be a consequence of the terminating precursor phase, and it can be related to the viewing angle. It was best studied in V1309 Sco, where the decrease in *I*-band flux (Fig. 1) was interpreted as caused by the buildup of circumstellar material obscuring the central binary at visual wavelengths in our line of sight (Pejcha et al. 2017). This model naturally explains the appearance of the dip at the latest phases of the precursor and the disappearance of the eclipses. Alternatively, Tylenda et al. (2011a) assigned the feature to an enhanced column of dust in the line of sight to the coalescing stars, but the timescales for this dust to be formed appear to be too short to explain the fast light-curve dimming.

***Main (blue) peak***

The rise to the main peak is sometimes interpreted as the first contact between the merging stars (the plunge-in phase), during which dense material is compressed and strong (hot) shocks form in the primary’s envelope (e.g., Soker and Tylenda 2006; Nandez et al. 2014). As the orbit tightens, the donor envelope can no longer remain synchronized with the binary, increasing the relative motion between the companion and the surrounding gas. Hydrodynamic simulations associate the resulting shocks and transfer of orbital energy and angular momentum with a transition from slower, largely bound mass loss to faster, partly unbound ejecta near the plunge-in phase (MacLeod et al. 2018a, b). This initiates the dynamical phase of the merger (Ivanova et al. 2013a). In the classical common-envelope evolution picture, the orbital energy of the binary is used to unbind (part of) the envelope, which then expands almost freely at velocities above the local escape velocity (Chen and Ivanova 2024). Most authors agree that the main peak is due to the release of thermal energy of this unbound, expanding, shock-heated gas. During this stage, the ejecta is mostly ionized and optically thick, forming a likely asymmetric pseudo-photosphere (see Sect. 3.4) that expands at velocities of a few hundred $\text {km s}^{-1}$. This phase resembles other eruptive events associated with strong winds, which may explain the characteristic main peak temperatures near 7500 K (Davidson 1987; Quataert et al. 2016) (cf. Fig. 5) and strong hydrogen emission. The interaction models of Kirilov et al. (2025) show that early shocks between merger ejecta and circumbinary material can also partially contribute to the total luminosity, but this effect does not dominate at peak light and appears sensitive to the initial conditions.

Soker et al. (e.g., Soker 2024, and references therein) advocate an alternative interpretation of the rapid luminosity rise during the main peak. In their *grazing-envelope evolution* model, which avoids a merger, the rapid rise is caused by jets forming near the more compact companion star and slamming into the surrounding material.

***Plateau (red peak)***

Early work by Ivanova et al. (2013b) adapted the framework of hydrogen recombination cooling, originally developed for type IIP SNe by Popov (1993), to account for the observed properties of red nova light curves. Within this scenario, several subsequent studies (e.g., Chen and Ivanova 2024; Matsumoto and Metzger 2022) have emphasized hydrogen recombination as the dominant power source of the extended plateau phases seen in luminous red novae. In these models, recombination energy is required to power the plateau because most of the initial thermal energy of the ejecta is lost to adiabatic expansion rather than being radiated away. Chen and Ivanova (2024) and Chen (2026) also proposed that during the plateau, the ejecta transitions from a radiation-dominated to matter-dominated regime, and that the formation of molecular hydrogen can power an additional, late-time lower-luminosity plateau. So far, recombination models have explained reasonably well red novae signatures for fainter transients such as V1309 Sco or AT2019zhd. However, the most luminous events seem to require ejecta masses significantly larger than those inferred from observations, or they struggle to reproduce the observed temperature evolution (cf. Matsumoto and Metzger 2022; Soker 2024).

The longer, redder peaks observed in the light curves of the ‘risers’ group were explained as a result of the interaction of the merger ejecta with the pre-existing circumbinary material. While the scenario was first introduced by Metzger and Pejcha (2017), Kirilov et al. (2025) investigated the interaction in detail and found that the structure and geometry of the pre-merger circumbinary material influence the amplitude and length of the plateau phase, analogous to circumstellar medium-interacting SNe (cf. Dessart et al. 2016). The ejecta can strongly energize the circumbinary matter so that it can remain bright even after the shock wave is gone. Substructures in the light curves (i.e., extra peaks or dips) can be directly linked to the structure of the circumbinary material (likely a torus) penetrated by the radiative shocks. In the scenario considered by Kirilov et al. (2025) (see also MacLeod et al. 2017), there is no special role of recombination energy in this phase of the outburst: hydrogen recombination does happen but does not increase the luminosity of the plateau and only slightly prolongs its duration. One possibility is that among red novae, different populations may require different mechanisms driving the plateau (red peak) phase. For instance, the ‘plateau’ and ‘risers’ groups (Sect. 3.1) may be physically different in that context.

To date, most simulations of red nova outbursts have assumed intrinsically spherical merger ejecta (but see Nandez et al. 2014). Nevertheless, asymmetries are expected to develop during the plateau phase in many merger models. In more recent hydrodynamical simulations, initially spherical ejecta interact with a dense circumbinary torus or disk, which redirects the flow and suppresses equatorial expansion. As a result, the ejecta expand preferentially along the polar directions, producing a bipolar outflow structure (Pejcha et al. 2017; Metzger and Pejcha 2017). An alternative interpretation is proposed by Soker (2024) within a non-terminal grazing-envelope evolution (GEE) scenario. In this framework, the ejecta consist of multiple components with different opening angles, ranging from highly collimated jets to wide-angle outflows. Their interaction with the surrounding medium generates a sequence of shocks and mass-ejection episodes that combine to form the observed plateau. However, detailed multi-dimensional simulations of the GEE scenario are still lacking.

The role of dust formation in shaping the light curve is an active topic of research, as dust condensation is notoriously difficult to implement in simulations of eruptive events (cf. González-Bolívar et al. 2024; Bermúdez-Bustamante et al. 2024; Mu et al. 2026). While some simulations of red nova outbursts find that dust formation does not strongly affect the light-curve evolution up to the end of the plateau phase (e.g., Chen and Ivanova 2024), dust formation in the remnant is an essential requirement to closely reproduce the observed light-curve shapes (Hatfull et al. 2021; Kirilov et al. 2025), color evolution, and the dust masses measured in young red nova remnants.

***Post-plateau decay***

The immediate post-plateau evolution is related to the dissipation of energy stored in the pre-merger torus and merger ejecta, but at much lower densities and temperatures than in the earlier phases (Kirilov et al. 2025). On longer timescales, the post-plateau relaxation phase is typically interpreted as partial gravitational contraction of the remnant and geometrical dispersal of the ejecta (Tylenda and Soker 2006; Schneider et al. 2016). Further modifications of the transient’s brightness can be due to infall activity (Goranskij et al. 2018). Low-amplitude changes years and decades after the eruption have been observed in many Galactic objects, but their origin is unclear. See Sect. 6 for discussions on the post-outburst relaxation in red nova remnants.

## The progenitors

Pre-outburst photometry of red novae comes from surveys that serendipitously covered the position of the progenitor years before the red nova outburst. The parameters of these systems are listed in Table 2, and mainly correspond to the primary, which in most cases is assumed to dominate the progenitor’s flux. The properties of the secondary components are much less constrained, and only in the case of V1309 Sco was the secondary directly observed before the red nova event. Table 2 covers a wide range of the primary masses, from ≈ 1 to 40 $\textrm{M}_{\odot }$. The progenitors also span a wide range of evolutionary stages, and include pre-MS stars, MS stars, sub-giants and yellow supergiants, RGB stars, AGB stars, and even a white dwarf and a planet. The red nova progenitors as a population are related to the red nova rates discussed in Sect. 5.2. Below, we first discuss the progenitor data for Galactic red novae before introducing the larger sample of known extragalactic progenitors. We next describe efforts to identify objects evolving into a red nova and emphasize the role of triples among red nova progenitors.

**Table 2:** Properties of red nova progenitors

Object	Merged binary	Evolutionary	Primary’s properties				Main
	($M_\odot$) (if known)	stage or type	*M* ($M_\odot$)	*L* ($L_\odot$)	$T_\textrm{eff}$ (K)	*R* ($R_\odot$)	refs
CK Vul	2+0.3	RGB+HeWD					[1]
V4332 Sgr	∼1	sub-giants?	$1^{+1}_{-0.3}$				[2]
V838 Mon	(5-10)+∼0.4	MS+pre-MS	$7.5^{+2.5}_{-2.5}$	900±200	17000±1000		[3]
BLG360		RGB+?	∼1				[4]
V1309 Sco	1.5+0.2	contact sub-giants	$1.5^{+0.5}_{-0.5}$	5.8±2.8	4500±200	∼1.6	[5]
ZTF SLRN-2020	(0.8−1.5)+planet	MS+super-Jupiter	$1^{+0.5}_{-0.2}$	∼0.3		∼1	[6]
M31-LRN-2015	(3-5)+(0.1−0.6)	sub-giant	$4^{+1.5}_{-1}$	600±200	5250±1250	30±10	[7]
AT2019zhd							[8]
AT2025abao		M-type AGB	$7^{+2}_{-2}$	$16\,000 \pm 4100$	3500±200	350±50	[9]
AT1997bs			$40^{+20}_{-20}$	$(1.6 \pm 0.9) \times 10^{5}$	13500±6500		[10]
N4490-OT2011		LBV? YSG?	$30^{+10}_{-10}$	$(1.3 \pm 1.2) \times 10^{5}$	14000±6500		[11]
N3437-2011OT1		YSG?	>10				[12]
UGC12307-2013-OT1		YSG?	>10				[13]
SNhunt248		YHG	$30^{+2}_{-2}$	$(4 \pm 1)\times 10^{5}$	6500±300	∼500	[14]
M101-2015OT1		YSG	$18^{+1}_{-1}$	$88\,000 \pm 8000$	6600±300	220±25	[15]
AT2015fx		YSG	$17.5^{+2.5}_{-2.5}$	$1 \times 10^{5}$	7250		[16]
AT2018bwo		YSG	$13^{+3}_{-2}$	$18\,000 \pm 1300$	6280±340	114±9	[17]
AT2018hso				$(4.7 \pm 3.0) \times 10^{5}$	13500±4000	125±50	[18]
AT2020hat		YSG (mid-K)	≲8	3700±600	4800±500		[19]
AT2021blu	5<q≲15	YSG	14±0.5	$(4.1^{+0.3}_{-0.3}) \times 10^{4}$	$6800^{+800}_{-600}$	$150^{+30}_{-30}$	[20]
AT2021biy	$3 \lesssim q \lesssim 10$	YSG	20.5±2.5	$(1.3\pm 0.1) \times 10^{5}$	7620±140	210±6	[20]

Notes: In the second column, *q* denotes the mass ratio of the more massive primary star to the secondary. References: [1] Tylenda et al. (2024); [2] Kamiński et al. (2018a); [3] Tylenda et al. (2005); [4] Tylenda et al. (2013); [6] Stȩpień (2011); [7] De et al. (2023); [8] Pastorello et al. (2021a); [9] Karambelkar et al. (2025); [10] Van Dyk et al. (1999, 2000); Adams et al. (2016); [11] Smith et al. (2016); [12] Pastorello et al. (2019b); [13] Pastorello et al. (2019b); [14] Mauerhan et al. (2015); [15] Blagorodnova et al. (2017); [16] Tartaglia et al. (2016); [17] Blagorodnova et al. (2021); [18] Cai et al. (2019); [19] Pastorello et al. (2021b); [20] Wavasseur et al. (2026)

### Galactic red nova progenitors

Characterizing the progenitors is challenging. The available archival pre-outburst photometry is often non-simultaneous and limited to a few bands, rarely reaching the IR regime. Additional problems are related to extinction correction (i.e., reddening) and field contamination. The extinction problem is especially complex because, in addition to the interstellar component, many of the progenitor stars have circumstellar dust, which may add an extinction component of a different characteristic than the canonical interstellar dust. Another challenge is the potential presence of emission lines in systems evolving toward red nova. V1309 Sco remains the only object whose progenitor was observed with a few-day cadence before the red nova outburst (Tylenda et al. 2011a) (Fig. 1). Even in this case, though, the observations were made quasi-simultaneously in only two photometric bands, at best. None of the Galactic red nova progenitors has been observed spectroscopically.

An additional complication, more relevant for Galactic objects than for the extragalactic ones, is the problem of their distances. Distances are required to constrain the source luminosity and, consequently, the evolutionary state. The only Galactic red nova with a well-known distance is V838 Mon. Its distance of 5.9±0.4 kpc has been constrained with several methods, including polarimetric light echo observations (Sparks et al. 2008) and interferometric maser parallax (Ortiz-León et al. 2020). Distances to other objects are often based on their reddening, ISM features, galactic radial velocity, or kinematical expansion of the ejecta. These methods carry substantial uncertainties, leading to luminosity and mass estimates that can vary by factors of a few.

To illustrate the challenges and the impact of uncertainties in characterizing progenitors, we revisit the case of V838 Mon, which erupted in 2002. Tylenda et al. (2005) summarized the available progenitor data, including photometric measurements in six bands spanning from *B* to $K_s$. In addition, Goranskij et al. (2004) examined a large collection of photographic plates from 1928 to 1994 and found no variability in the *B* and *V* bands, justifying the comparison of data obtained at widely separated epochs. This archival effort, later updated in Goranskij et al. (2007, 2020), provides one of the longest—albeit irregular—historical records for a red nova progenitor.

Early post-outburst spectra revealed that V838 Mon has a distant companion whose spectral features correspond to a B3 or B4 MS star (luminosity class V). Tylenda et al. compared the observed fluxes with standard stellar colors compiled from the literature (largely based on now outdated calibrations from the 1980 s). Because the interstellar extinction toward the object was poorly constrained, they considered two values of E(B-V), 0.9 and 0.71 mag. In their analysis, they assumed that the low-mass secondary consumed in the merger did not contribute significantly to the spectral energy distribution (SED), and modeled the system as the combined light of the merger primary and the distant blue companion. They found that the combinations B3V+B1.5V and B4V+A0.5-protostar best reproduced the observations for the two assumed reddening values, respectively.

For comparison, using a more modern calibration of stellar colors and spectral types from Pecaut and Mamajek (2013) and adopting E(B-V)=0.9 mag, one obtains a B3V+B3V configuration (cf. Tylenda and Kamiński 2012). The inferred stellar masses remain highly uncertain: masses for B3V stars span ∼ 5–10 $\textrm{M}_{\odot }$ in the literature, and the range becomes even broader when other spectral types are considered (down to ∼2 $\textrm{M}_{\odot }$ in the A0.5 protostar scenario). The V838 Mon system is a member of an open cluster of B-type stars (Afşar and Bond 2007), yet the progenitor and its companion are ∼ 1.3 mag fainter than other cluster members. This discrepancy has been investigated (Tylenda and Kamiński 2012; Goranskij et al. 2020), including the possibility of patchy extinction across the cluster, but no satisfactory explanation has been found. Possible interpretations include circumstellar or circumbinary extinction, rotational deformation, or a higher luminosity class (IV) for the components. Interestingly, a somewhat more evolved (and dimmer) evolutionary state for the progenitor has also been proposed on theoretical grounds, where most of the pre-outburst contribution would be attributed to the distant tertiary star (Temmink et al. 2025).

The case of V838 Mon highlights how difficult it is to reliably identify progenitor properties, even when relatively extensive historical data and a well-constrained distance are available. The mass of the engulfed companion was estimated to be 0.1–0.5 $\textrm{M}_{\odot }$ (Tylenda and Soker 2006), representing the minimum accreted mass required to power the observed radiative and kinetic output of the outburst. These estimates assume a primary mass of 8 $\textrm{M}_{\odot }$ and a radius of 7.5 $\textrm{R}_{\odot }$. A rigorous propagation of uncertainties shows that most parameters describing the progenitor are uncertain to within at least an order of magnitude. Consequently, stellar mass estimates for the objects listed in Table 2 should be treated with caution: at best, they allow a classification into broad categories such as low-, intermediate-, or high-mass stars, rather than precise determinations. The most robust parameter that can currently be inferred is the evolutionary stage of the progenitor system.

Tracing back the evolution of the identified progenitor binaries is not an easy task either. As mentioned, V1309 Sco’s progenitor was the only system evolving into a merger with both components observed in the photometric data. The observations showed a low-mass binary in deep contact, similar to W UMa systems but with a slightly longer orbital period of 1.43 days and in a more advanced evolutionary stage. Several studies attempted to model the evolution of V1309 Sco leading to the state in which it was observed in the 2001–2008 period, just before the red nova event. That of Stȩpień (2011) was most comprehensive because it attempted to reproduce the evolution starting at zero-age main sequence (ZAMS). In his best model, the object starts as a 1.2+0.5 $\textrm{M}_{\odot }$ binary with an orbital period of 2.8 days. Magnetic braking causes loss of angular momentum; the orbit shrinks, and the radius of the primary increases as it evolves to the sub-giant branch. After 5.4 Gyr, the system undergoes the first Roche lobe overflow (RLOF) mass transfer phase and a successive common-envelope phase, which produces an Algol-like system with inverted masses of 0.51+1.22 $\textrm{M}_{\odot }$ and a significantly shorter orbital period of 1.2 days. The mass transfer continues but at a much lower rate, and the now more massive star evolves toward the RGB, building up a helium core. This continues until Darwin instability triggers the merger. At the age of 7.85 Gyr, the system has the properties of the observed progenitor of V1309 Sco: a contact binary with a mass of 0.16+1.52 $\textrm{M}_{\odot }$, orbital period of 1.44 days, and a surface temperature of ∼ 5000 K. Both components have helium cores of ≈0.15 $\textrm{M}_{\odot }$ at the moment of the red nova event. This detailed model is very fine-tuned to V1309 Sco and should not be generalized, especially to W UMa systems. Despite the many uncertainties—including mass loss, strength of magnetic fields, assumed internal structure of the stars—the model of Stȩpień (2011) remains the most sophisticated attempt to model a red nova progenitor.

### Extragalactic red nova progenitors

Detections of extragalactic red nova progenitor systems were usually possible using archival images from space-based (Hubble Space Telescope, Spitzer Space Telescope; see Smith et al. 2016; Blagorodnova et al. 2021) and deep ground-based images (e.g., Blagorodnova et al. 2017; Pastorello et al. 2021b). Because of the limiting magnitudes of these datasets, usually only the brighter stellar progenitors (or the closest ones) were detected. Currently, as shown in Table 2, there are 15 extragalactic systems (out of 23 listed in Table 1) with a direct archival detection of a progenitor.

**Fig. 8:**
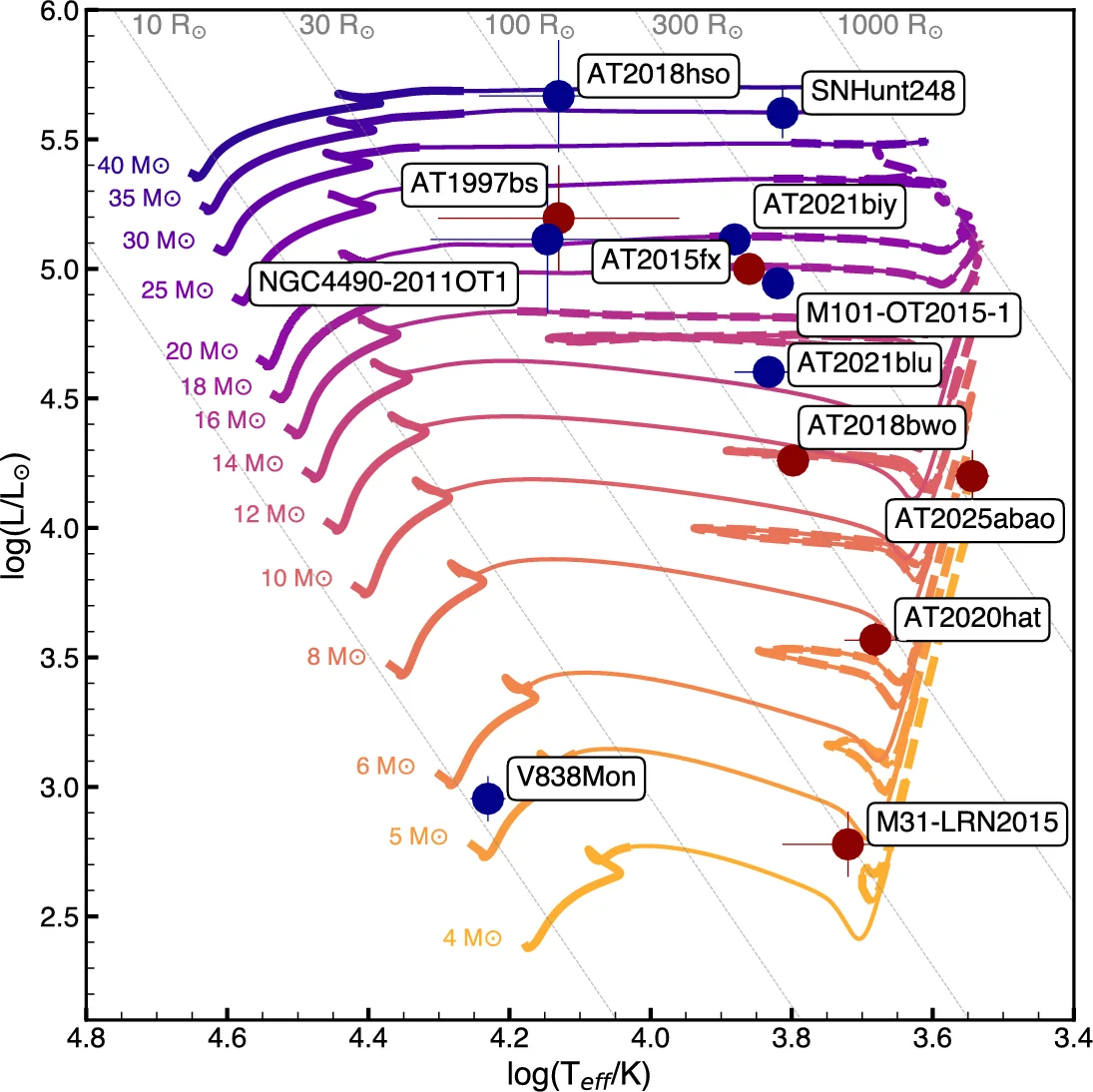
Luminosities and effective temperatures for selected red nova progenitors. Drawn lines show MESA MIST models (Dotter 2016; Choi et al. 2016) for initial masses between 4 and 40 $\textrm{M}_{\odot }$ at solar metallicity and 0.4 critical rotation. The main sequence is marked with thick solid lines. The transition to core He burning is represented by thin lines. The He core burning and later phases are represented by dashed lines

The majority of archival images were obtained in at least two photometric bands. It was possible to derive the color and intrinsic luminosity of the more evolved and massive primary star, which was assumed to dominate the light of the whole system. To determine the mass range and evolutionary state of the primary star, some studies compared the observed magnitudes in different filters with synthetic photometry provided by grids of stellar evolution tracks (Cai et al. 2022b). Other studies derived the stellar SEDs using blackbody, modified blackbody, or model-atmosphere synthetic spectra, and then compared them with model grids of single and binary evolutionary tracks (Blagorodnova et al. 2017; MacLeod et al. 2017; Pastorello et al. 2021b). Figure 8 places these sources in the Hertzsprung–Russell (HR) diagram.

The dominant spectral types for red nova progenitors appear to be yellow supergiants, which are stars in fast expansion after the end of their hydrogen core burning (i.e., in the Hertzsprung gap). This dominance of a single type of star in the observed population is striking and contrasts with the broader range of progenitor types of the less-luminous red novae known from the Milky Way. Some less massive progenitors that might break this extragalactic monopoly are AT2020hat (a K-type supergiant) and AT2025abao (an AGB star), and possibly a few luminous stars that share similarities with LBVs. The apparent lack of evolved donors, especially in systems with <5 $\textrm{M}_{\odot }$, has been attributed to dust obscuration: prolonged, non-conservative mass transfer can cause gas to accumulate, cool, and form dust around the binary, hiding the final ejection event (MacLeod et al. 2022).

A few studies developed detailed binary evolution models, often using the stellar evolution code MESA (Modules for Experiments in Stellar Astrophysics; Paxton et al. 2011, 2013, 2015, 2018, 2019; Jermyn et al. 2023), to characterize the pre-merger systems during quiescence. These included more comprehensive descriptions of physical properties of the binary (donor’s mass and evolutionary stage, mass ratio, and period), mass-transfer rates (Blagorodnova et al. 2021), and different orbital instability criteria (Wavasseur et al. 2026). These model-supported studies converge on the conclusion that the extragalactic progenitors observed as yellow supergiants likely underwent a delayed dynamical instability (DDI; Hjellming and Webbink 1987). In this evolutionary channel, the Roche lobe overflow begins as stable mass transfer but progressively accelerates as the donor’s structure evolves, ultimately triggering dynamical instability and leading to a merger on timescales of a few hundred years. During this phase, the donor star experiences a substantial decrease in luminosity by up to an order of magnitude (cf. Temmink et al. 2025), as energy from the inner regions is diverted into stellar expansion rather than transported outward as radiative luminosity. Observationally, this implies that donors undergoing high mass-transfer rates may appear anomalously faint and can therefore be misidentified as lower-mass stars when interpreted using single-star evolutionary models.

### Searches for systems directly evolving into red novae

The identification of a change in period in the eclipsing binary progenitor of V1309 Sco has fueled multiple attempts to predict the next red nova. For example, Soker and Tylenda (2006) proposed searching for changes in orbital periods of W UMa contact binary systems with low mass ratios. Indeed, W UMa binaries with critically low mass ratios, q<0.09, have long been believed to be tidally unstable and particularly prone to Darwin instability (Rasio 1995; Pešta and Pejcha 2023). Recent studies present a more complex view on which binaries merge and on the necessary time scales (e.g., Wadhwa et al. 2024; Jiang et al. 2026, and references therein). Current W UMa catalogs seem to include a few candidates within the instability parameter space (e.g., Wadhwa et al. 2025; Jiang et al. 2026; Poro et al. 2026), but problems with deriving precise stellar parameters and excluding the ‘third light’ (or other tertiary companion effects) have hampered definitive identifications (Kurtenkov 2017; Wadhwa et al. 2024). The search continues with multiple observing campaigns (e.g., Gazeas et al. 2021; Kobulnicky et al. 2022). Since W UMa stars are relatively frequent (0.2% in the solar neighborhood, Rucinski 2006), the scarcity of the likely merger candidates suggests that not many red nova progenitors can be identified this way without considerable improvement in the sensitivity of our observations, which may soon become feasible with Rubin-LSST (Hambleton et al. 2023). We also note that the progenitor of V1309 Sco was already in an advanced evolutionary stage when first observed, as it was a sub-giant or a giant (Stȩpień 2011), while most W UMa stars are considered to be on the main sequence. Kobulnicky et al. (2022) found that only W UMa systems evolving off the main sequence have a chance to develop the Darwin instability and merge.

Stimulated by the V1309 Sco’s progenitor properties, there have also been more general searches of binary systems with rapidly decreasing orbital periods. Pietrukowicz et al. (2017) browsed the vast OGLE repository, and among over 22,000 systems with short periods, they found 56 binaries with some period decrease. The maximal decay rate found is of −1.94× $10^{-4}$ day $\hbox {year}^{-1}$. In the same sample, however, Pietrukowicz et al. found 56 systems with increasing periods, which led them to conclude that all the observed period changes, positive and negative, are most likely due to the presence of a third body (or due to stellar spots). One system with a decreasing period caught special attention, when Molnar et al. (2017) identified a W UMa system, KIC 9832227, in the *Kepler* field. Multiepoch observations acquired with different telescopes suggested that the object had a decaying orbit, somewhat similar to V1309 Sco. In 2017, the authors predicted that the system would merge in 2022. However, a few years before the awaited merger, an error in the analysis was found (Socia et al. 2018), and the system’s variable period was explained by a third body (Kovacs et al. 2019; but see Salinas and Howell 2023). KIC 9832227 did not become a red nova. It seems, therefore, that finding red nova progenitors based solely on orbital changes is very difficult and often misleading. In fact, based on theoretical considerations linking orbital decay to unstable mass loss, MacLeod and Loeb (2020b) showed that the first time derivative of the orbital period alone is insufficient to identify systems approaching merger, and that the second derivative provides a more robust diagnostic (see also Pejcha 2014; Pejcha et al. 2017).

Yet, another potential method of identifying red nova progenitors relies on a slow and steady increase in brightness of the progenitor system, i.e., during the precursor phase (see Sect. 3.1), which may be associated with the pre-merger inspiral (Sect. 3.5; e.g., MacLeod and Loeb 2020b; Pejcha et al. 2017), or heating caused by a drag force (Bronner et al. 2024, Karambelkar in prep.). The slow rise has a much lower amplitude than the outburst, typically ≲ 3 mag. Based on the notion that many of the extragalactic luminous red novae had precursors with properties consistent with yellow supergiants, Addison et al. (2022) and Tranin et al. (2025) searched photometric archives of the Milky Way and the Local Group galaxies for yellow supergiants that mildly increased their brightness. Additionally, Garcia-Moreno et al. (2026) attempted to identify emission line stars in the Hertzsprung gap that showed variability. Since yellow supergiants are rapidly evolving, stringent selection criteria could be applied to find them in monitored galaxies. It was found that M31, M33, and M101 are the galaxies where spotting a red nova precursor is most likely and that these precursors come from primaries with masses close to either 5 or 10 $\hbox {M}_{\odot }$. Several Galactic and twelve extragalactic candidates for luminous red nova precursors were proposed and await verification. The searches of Tranin et al. (2025) and Addison et al. (2022) for yellow supergiant precursors are probably the most comprehensive (volume-wise, considering candidates in hundreds of galaxies) studies to date. They are, however, somewhat biased toward a certain subtype of red novae progenitor and source selection was based on sometimes uncertain extinction corrections for the Milky Way and nearby galaxies. This approach will turn out to be especially potent for finding red nova progenitors in the era of the Rubin-LSST survey (Hambleton et al. 2023).

### Mergers in triple systems

Although this topic remains relatively unexplored in the literature, there is growing evidence to suspect that a significant fraction of red nova progenitor systems are triples or higher-order multiples. V838 Mon remnant certainly has a surviving companion on a wide orbit of a few 100 au, so, within the merger hypothesis, the collision took place in a hierarchical triple (Desidera and Munari 2002; Kamiński et al. 2021b). Additionally, the structure and kinematics of the molecular outflows of CK Vul observed some 300 years after its eruption suggest that they arise from ejecta interaction with a high-gravity (e.g., a dwarf) companion (Kamiński et al. 2021a). This implies a triple system as the progenitor. Moreover, the majority of progenitors of the extragalactic red novae belong to the 10–30 $\textrm{M}_{\odot }$ population (Table 2) for which the fraction of triples is 60–70% (Moe and Di Stefano 2017; Offner et al. 2023).

Triggering an instability that could lead to a merger is dynamically easier in hierarchical triples than in binaries (e.g., through the Kozai–Lidov cycles or as in Glanz and Perets 2021), and therefore, searching for triple progenitors among red novae is fully justified (e.g., Toonen and Boekholt 2022; Shariat et al. 2025; Kummer et al. 2025; Perets and Fabrycky 2009; Perets 2026), as much as it is hard in practice. The potential presence of a surviving companion in red nova remnants, however, puts extra uncertainties on our characterization of the remnants, as the companion would be an extra heat source that needs to be accounted for in SED analysis and may trigger shocks through interactions of the passing ejecta (cf. Kamiński et al. 2021b, Sect. 6).

## Red novae as a population

### Scaling relations

**Fig. 9:**
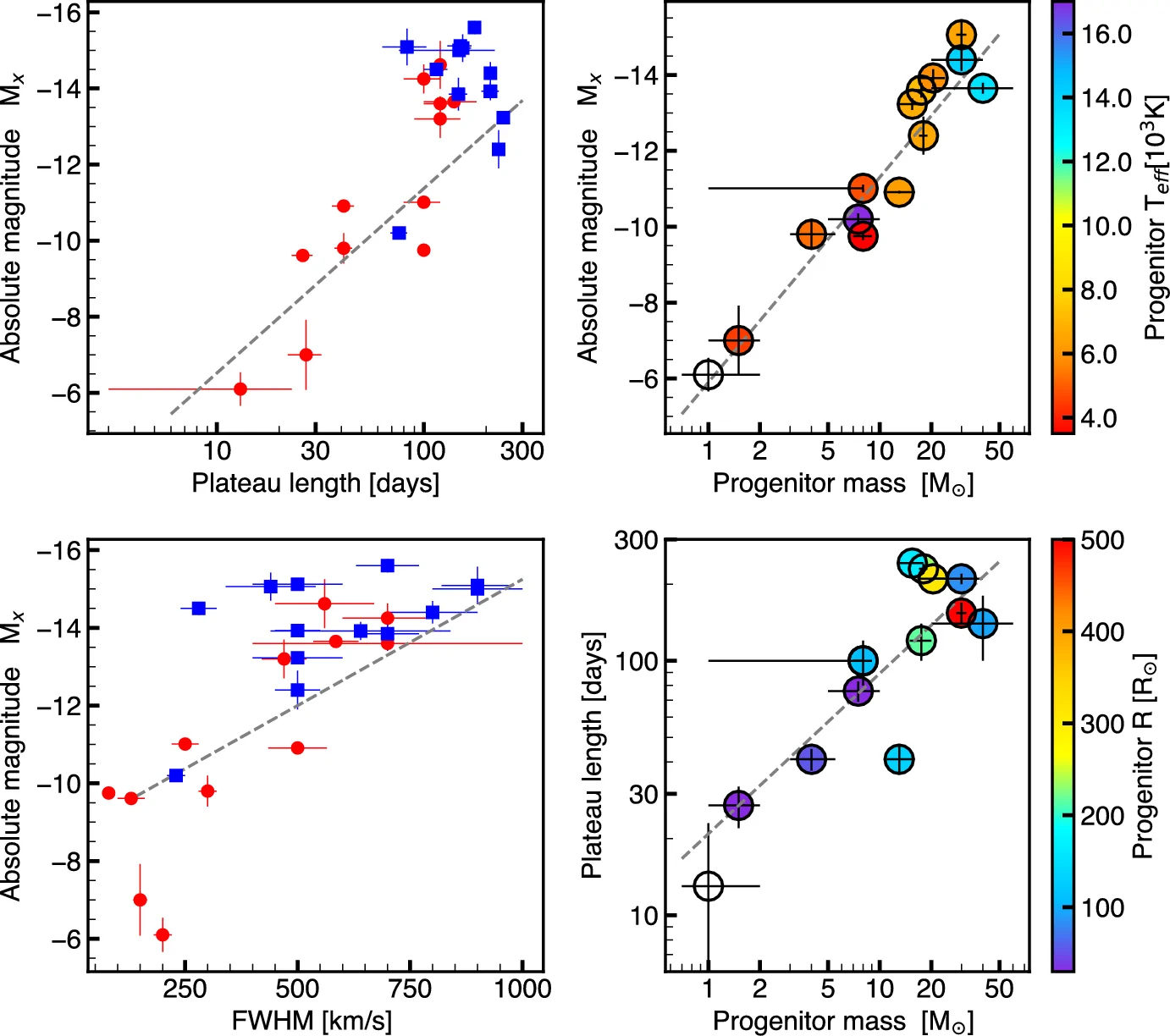
Scaling relations for red novae in Table 1. Left panels: comparison of observable properties relative to the outburst. Blue squares show the population of the ‘risers’ group, while the red circles represent the ‘plateau’ group (cf. Sect. 3.1). Absolute magnitudes refer to bands as in Table 1, where *x* refers to *V/R/r* band. The FWHM represents typical linewidths during the eruption, as in Table 1. Right panels: comparison between the properties of the primary progenitor star and the outburst properties of red novae. Points are color-coded by the progenitor’s temperature and radius (cf. Sect. 4 and Table 2; a white color indicates a lack of information)

Red novae represent a highly heterogeneous group. Although they are considered ‘intermediate-luminosity transients’, the faintest objects, such as V1309 Sco or V4332 Sgr, clearly overlap with the average luminosity of classical novae and the brightest red nova events are comparable to faint SNe (cf. Fig. 2). The spread in visual absolute magnitudes of nearly 10 mag suggests a range of physical mechanisms driving the red nova outburst. The red nova diversity is also emphasized by the wide variety of light curves, including their time spans and shapes (see Sect. 3.1). Among this apparent variety, recent studies explored possible correlations between the properties of red novae and of their progenitor systems that could be generalized for this entire transient family. Some of these correlations are shown in Fig. 9, and our best-fitting relations applied to data in Tables 1 and 2 are listed in Table 3.

**Table 3:** Best-fitting linear relations

Relations from Kochanek et al. (2014)	
$$M_V =-7.2(\pm 1.5) \times \log _{10}(M_\textrm{prog} [M_{\odot }])-3.6(\pm 0.8)$$	
$$M_I =-5.1(\pm 0.7) \times \log _{10}(M_\textrm{prog} [M_{\odot }])-5.6(\pm 0.4)$$	
Relations from Cai et al. (2022b)	
$$M_V =-5.56(\pm 0.69) \times \log _{10}(M_\textrm{prog} [M_{\odot }])-4.91(\pm 0.26)$$	
Relations* for data in Tables 1 and 2	
$M_X =-4.85(\pm 2.06) \times \log _{10} (t_\textrm{plateau}[d]) - 1.67(\pm 4.22)$	$\chi ^2_r$=0.20
$M_X =-5.4(\pm 0.6) \times \log _{10}(M_\textrm{prog} [M_{\odot }])-5.9(\pm 0.7)$	$\chi ^2_r$=0.23
$M_X =-0.0065(\pm 0.0040) \times \textrm{FWHM}[\mathrm {km\,s}^{-1}] - 8.75(\pm 1.98)$	$\chi ^2_r$=10.0
$\log _{10} (t_\textrm{plateau}[d])=0.63(\pm 0.16) \times \log _{10}(M_\textrm{prog} ([M_{\odot }]) +1.32(\pm 0.18)$	$\chi ^2_r$=0.04

*Error-weighted fits to data using the nonlinear least-squares Marquardt–Levenberg algorithm and the corresponding reduced Chi-square values ($\chi ^2_r$). $M_X$=$M_r$, $M_R$, or $M_V$ as in Table 1

Early studies proposed different parameterizations to uncover scaling relations in red novae. Based on a handful of events known at that time, Kulkarni et al. (2007) proposed a relation between the peak magnitude and the duration of the outburst on a log scale. Figure 9 shows that for the updated sample of red novae, these two properties are somewhat related (although with some scatter), especially for the members of the ‘plateau’ group; the ‘risers’ generally occupy the high-luminosity and high-duration region of the diagram. This correlation was also shown in the theoretical work of Ivanova et al. (2013b), which interpreted red novae as recombination-powered transients. In their model, the plateau luminosities and durations of the light curves are linked to the amount of unbound mass ejected during the merger, its kinetic energy, and the initial launch radius of the outflow. This framework naturally accounts for the observed scatter: for progenitor stars of equal mass, the plateau luminosity is expected to be broadly similar, while the plateau duration can increase up to an order of magnitude for more evolved systems.

In a complementary approach, Kashi and Soker (2010) and Soker and Kashi (2011) considered that all intermediate-luminosity transients are powered by mass accretion from a companion onto an MS star. These authors introduced an Energy Time Diagram, which displays the total energy of the transients (radiated plus kinetic) as a function of the duration of the eruption, defined as a 3-mag decline in the *V*-band. In this diagram, intermediate-luminosity transients occupy a diagonal band, revealing a nearly linear correlation between total energy and outburst duration. The lowest energy events in this sample correspond to the planet–brown dwarf merger scenario (Bear et al. 2011; Metzger et al. 2012), with characteristic timescales of only a few days. The central part of the diagram is dominated by red novae, with durations between a month and a year, and the highest energy events correspond to the long-lasting LBV eruptions analogous to η Car’s Great Eruption. This representation links red novae to the broader population of intermediate-luminosity transients.

The detection and characterization of the progenitor stars has also made it possible to relate the properties of the quiescent system with the resulting outburst. The early work of Kochanek et al. (2014), based on four Galactic red novae with progenitors known at that time, proposed the existence of a correlation between the progenitor’s mass and peak luminosity of the transient as $L \propto M^{2.9\pm 0.6}$ (see Table 3). More massive progenitors, such as that of V838 Mon, generated brighter events than low-mass progenitor systems like V4332 Sgr or V1309 Sco. This correlation, depicted in Fig. 9, has now been expanded to the more luminous and massive extragalactic red novae (e.g., Blagorodnova et al. 2021; Cai et al. 2022b) and the mass–luminosity relation was further revised to $L \propto M^{2.22 \pm 0.28}$ (see also Table 3). Pastorello et al. (2023) has also shown that the progenitor magnitude (proxy for mass) not only correlates with the luminosity of the main blue peak, but also the magnitude of the second red peak and the magnitude of the precursor. Notably, their sample shows that among events with durations shorter than 100 days (mostly members of the ‘plateau group’ defined above), the duration is shorter when the first blue peak is substantially more luminous than the red peak. In contrast, for events lasting longer than 100 days (the ‘risers group’), no clear correlation is observed between the blue-to-red peak luminosity ratio and the event duration.

The theoretical model of Pejcha et al. (2016b) suggested that the luminosity of red transients is proportional to the asymptotic outflow velocity measured for these events, as L2 mass loss and inter-shocks would be the main power mechanism for red nova emission. Although their study included objects since then removed from the red nova category (e.g., SN2008S or NGC 300-OT), Fig. 9 shows that this correlation holds, albeit with a large scatter, which increases for events brighter than − 10 in absolute magnitude. It is important to note, however, that velocities are hard to measure, and a significant fraction of the observed scatter likely reflects methodological differences in how and at what phase the velocities are measured.

While velocity measurements inferred from narrow absorption lines may be a more precise way to derive the ejecta speed, the lack of medium- and high-resolution data for the observational sample complicates this approach. Consequently, the width of the usually broader and stronger Hα line has been commonly used as a proxy for the bulk ejecta velocity. The drawback is that this line has been shown to exhibit electron scattering wings around the main peak, and often displays multiple and evolving absorption components that are challenging to model. The overall FWHM has also been shown to vary with time (see Fig. 13 from Pastorello et al. 2023), with the highest values reported during the first optical peak, so the lack of a standardized reference epoch to report FWHM measurements further complicates comparisons across different studies. In some cases, spectra could only be obtained after peak luminosity, or were taken at insufficient spectral resolution, introducing additional systematic uncertainty into the inferred velocities.

Interestingly, Pastorello et al. (2023) showed that the Hα line luminosity measured at +7 days post-peak exhibits significantly less scatter than the corresponding Hα FWHM values reported at the same epoch, and correlates more tightly with the peak bolometric luminosity. This suggests that while the total energy radiated in the line is relatively stable, variations in ejecta geometry and line-forming conditions can introduce substantial additional scatter in FWHM-based velocity estimates.

### Event statistics

Constraining the total number of red nova events is a very difficult task. Estimates based on the observed rates in the Milky Way and nearby galaxies face the problem of completeness and selection bias, whereas population-synthesis models must include the largely unconstrained physics of mass and angular momentum transfer at different evolutionary phases of the binary, including the still poorly understood common-envelope evolution. Nevertheless, notable attempts to calculate red nova rates have been made. A summary and a comparison of the red nova rates to other transients (classical novae, supernovae, and kilonovae) is shown in Table 4.

**Table 4:** Representative rates of red novae and other transient classes

Transient class	Representative rate	Normalization and selection	Reference
Classical novae—MW	$26\pm 5~\textrm{year}^{-1}$	Intrinsic Galactic rate completeness-corrected	Kawash et al. (2022)
Classical novae—M31	$40^{+5}_{-4}~\textrm{year}^{-1}$ $65^{+16}_{-15}~\textrm{year}^{-1}$	Intrinsic galaxy-wide rates corrected for survey coverage, cadence, and detection efficiency	Rector et al. (2022)Darnley et al. (2006)
Classical novae—local volumetric rate	$6\times 10^{-2}~\textrm{Mpc}^{-3}\,\textrm{year}^{-1}$	Inferred from the luminosity-specific nova rate and the *K*-band luminosity density; not a direct volumetric measurement	Shara (2006)
Red novae—MW, observational estimates	$>0.019~\textrm{year}^{-1} L_\textrm{MW} \textrm{year}^{{-1}}$ at 95% confidence level ($L_\textrm{MW}$: galaxy luminosity) $\sim 0.5~\textrm{year}^{-1}$ for $M_\textrm{peak}\lesssim -3$; $\sim 0.03~\textrm{year}^{-1}$ for $M_\textrm{peak}\lesssim -10$;	The luminosity-function estimates are based on a few events and depend strongly on completeness and luminosity threshold	Ofek et al. (2008)Kochanek et al. (2014)Kochanek et al. (2014)
Red novae—MW, population synthesis	$\sim 0.1~\textrm{year}^{-1}$	Intrinsic COMPAS prediction over a broad luminosity range; dependent on binary evolution and transient-energy prescriptions	Howitt et al. (2020)
Luminous red novae—local volumetric rate	observed: $7.8^{+6.5}_{-3.7}\times 10^{-5}\,\textrm{Mpc}^{-3}\,\textrm{year}^{-1}$ population synthesis: $\sim 8\times 10^{-4}\,\textrm{Mpc}^{-3}\,\textrm{year}^{-1}$	ZTF–CLU value for $-16\le M_r\le -11$ magIncludes common-envelope population	Karambelkar et al. (2023)Howitt et al. (2020)
Type Ia SNe	PTF:$2.43\pm 0.29^{+0.33}_{-0.19}\,10^{-5}\textrm{Mpc}^{-3}\,\textrm{year}^{-1}$ZTF–BTS:$(2.35\pm 0.24)\,10^{-5}\textrm{Mpc}^{-3}\,\textrm{year}^{-1}$	Local volumetric rates. The ZTF–BTS value is integrated approximately to M<-16.5 mag in *g* or *r* and is not corrected for host extinction	Frohmaier et al. (2019); Perley et al. (2020)
Core-collapse SNe	PTF: $9.10^{+1.56}_{-1.27}\times 10^{-5}~ \textrm{Mpc}^{-3}\,\textrm{year}^{-1}$; ZTF–BTS: $1.01^{+0.50}_{-0.35}\times 10^{-4}\,\textrm{Mpc}^{-3}\,\textrm{year}^{-1}$	Local volumetric rates. The ZTF–BTS estimate includes events brighter than approximately M=-14 mag and is not corrected for host extinction	Frohmaier et al. (2019); Perley et al. (2020)
Kilonovae(binary neutron star mergers. BNS)	7.6–$250\,\textrm{Gpc}^{-3}\,\textrm{year}^{-1}$;ZTF–BTS optical limit: $<5\times 10^{3}\,\textrm{Gpc}^{-3}\,\textrm{year}^{-1}$ for	The BNS merger rate is used as an intrinsic-rate proxy.Luminosity-limited, M<-16, upper limit from no BTS detections	Abac et al. (2026); Perley et al. (2020)

Rates quoted per galaxy and volumetric rates are distinguished explicitly. The estimates are not fully homogeneous: some are intrinsic galaxy-wide rates, whereas others are luminosity-limited volumetric measurements or population-synthesis predictions. MW denotes Milky Way. BTS is the ZTF Bright Transient Survey (Fremling et al. 2020)

From purely observational perspective, Soker and Tylenda (2006) estimated that bright transient merger events (‘mergebursts’) occur in a Milky Way-type galaxy once every 10–-50 years. This estimate was derived by scaling the formation rate of blue stragglers inferred by Ciardullo et al. (2005) for old stellar populations, and then adjusting this rate for a much narrower and lower-mass range of secondaries expected for stellar mergers. The prediction applies to the most violent and luminous merger events, such as V838 Mon-like eruptions, and does not address the occurrence rate of fainter, lower-energy red nova events. Their rate is in agreement with the observational lower limit of 0.019 $\hbox {year}^{-1}$ for a Milky Way-type galaxy (Ofek et al. 2008)

Kochanek et al. (2014) performed a more systematic study of red nova rates. In their Bayesian analysis anchored in the detection of three Galactic events (V4332 Sgr, V838 Mon, and V1309 Sco), they found that the number of red novae scales with luminosity as $\textrm{d}N/\textrm{d}L \propto L^{-1.4\pm 0.3}$. They expect 1.3 events per 10 years for the transients of the luminosity of V1309 Sco or brighter, and expect only one event of the magnitude of V838 Mon every 30 year in a galaxy like ours. These estimates were in good agreement with rates in the population-synthesis results of Belczynski et al. (2022). Kochanek et al. noted that the red nova population should be dominated by MS stars merging with evolved stars, with only a small fraction (20%) being MS-MS pairs. The study also pointed to a strong relation between the peak luminosity and the mass of the progenitor for the known events of $L\propto M^{2.5\pm 0.5}$ (see Sect. 5.1 and Table 3).

Utilizing the binary population synthesis, Howitt et al. (2020) and Twum et al. (2026) simulated a large population of binaries and estimated the light-curve properties for those deemed to be unstable with recombination emission models (Ivanova et al. 2013b; MacLeod et al. 2017). As a novelty, Howitt et al. (2020) parametrized different assumptions about the kinetic energy of the unbound mass released during the merger, which represented an additional energy sink to fully unbind the envelope. Their approach predicted an intrinsic Galactic event rate of 0.1 $\hbox {year}^{-1}$, and a volumetric rate of 8$\times 10^{-4}$ $\hbox {Mpc}^{-3}$ $\hbox {year}^{-1}$. Twum et al. (2026) replaced the commonly used tabulated core–envelope binding energy parameter λ with detailed stellar structure profiles computed using MESA for single-star donors, enabling a more consistent estimate of the ejecta mass across a range of binary configurations. Although no specific rate was provided by this latter study, both works predicted a bi-modal distribution of plateau luminosities in their populations, corresponding to fainter merger events and brighter full common-envelope ejections (leaving a compact surviving binary). In practice, detections from magnitude-limited surveys such as Zwicky Transient Facility (ZTF) and LSST are expected to be dominated by the brighter class.

Whereas most earlier studies derived red nova rates primarily from Milky Way events, Karambelkar et al. (2023) instead based their estimates on transient detections in nearby galaxies (<150 Mpc) from the Census of the Local Universe (CLU) survey conducted with the ZTF. After applying completeness corrections to their identification of six (plus five tentative) red novae, they derived a volumetric rate of red novae of $7.8^{+6.5}_{-3.7} \times 10^{-5}$ $\hbox {Mpc}^{-3}$ $\hbox {year}^{-1}$ for absolute *r*-band magnitudes between -16 and -11 (i.e., comparable in brightness to V838 Mon or brighter). This rate is comparable to core-collapse SN rate of $10.1^{+5}_{-3.5} \times 10^{-5}$ $\hbox {Mpc}^{-3}$ $\hbox {year}^{-1}$ measured by the ZTF surveys (Perley et al. 2020). Since their experiment was sensitive to brighter events than those known from the Milky Way, they were able to demonstrate that the rate is a steep function of luminosity, $\propto L^{-2.5}$, for absolute magnitudes ≲ – 10. It is therefore steeper than proposed by Kochanek et al. (2014) for the Milky Way events (broadly reproducing the initial mass function), but is shallower than the binary population synthesis of Howitt et al. (2020). The broken power law for the rates as a function of luminosity is interesting as it may manifest different outburst mechanisms. In line with previous theoretical work, Karambelkar et al. (2023) speculated that the lower-luminosity events are caused by coalescing stars, while their high-luminosity analogs are a result of non-terminal common-envelope ejections in massive binaries.

The estimates of Howitt et al. (2020) and Karambelkar et al. (2023) of the observational rates of the most luminous (thus most massive) red novae seem to be comparable to compact mergers that are potentially detectable through contemporary gravitational-wave observatories. This notion may initially imply that some of the most luminous transients are indeed related to progenitors of the merging black hole and neutron star systems (see also Twum et al. 2026). Their common-envelope phase is hypothesized to be necessary to shrink the orbits of these systems before they spiral into a merger. Nevertheless, the binary population synthesis-based analysis of Jain et al. (2025) found that the fraction of red novae associated with an observed gravitational-wave signal (from a binary neutron star or a neutron star-black hole) would be as small as $\sim 10^{-3}$, implying that the vast majority of transients will lead to mergers, and not to compact systems.

As of 2026, the predicted red nova rates are difficult to reconcile with the absence of any newly identified bright Galactic red nova since V1309 Sco in 2008, excluding the much fainter planetary-engulfment event ZTF SLRN-2020. By contrast, three red novae were identified in neighboring M31 between 2015 and 2026. Based on the published rate estimates, at least one additional bright Galactic event would have been expected since the eruption of V1309 Sco. Perhaps our estimates were overly optimistic. This discrepancy may reflect statistical fluctuations, poor knowledge about the patchy Galactic extinction, or some sort of selection bias. For example, the mentioned studies of red nova rates predict a higher frequency for low-luminosity events that are supposedly missed in the contemporary data. Note that most of the studies were performed before the detection of the planet-induced (subluminous) red nova of De et al. (2023).

Because red nova rates are so strongly coupled to the crucial aspects of binary star evolution, there is a hope that future sensitive sky surveys will be able to provide more robust rates in the Milky Way and the Local Group. For instance, it is expected that the optical photometric survey of LSST-Rubin (Hambleton et al. 2023) will be able to observe 20–1500 red novae per year (Howitt et al. 2020; Karambelkar et al. 2023). Assuming the duration of the LSST-Rubin project of 10 years, one could expect a significant increase in the number of red novae in the Milky Way and nearby galaxies, but it remains uncertain how many will be robustly identified and followed up spectroscopically. There are also expectations that, in addition to merger and common-envelope transients, LSST-Rubin can provide another example of a close binary spiraling into a merger like V1309 Sco (Hambleton et al. 2023).

Most of the rate estimates performed so far have focused on optical detections of these transients. Since some red novae are expected to be dust-obscured even before the main outburst, some events will be intrinsically infrared (Jencson et al. 2019b; MacLeod et al. 2022). Comprehensive IR sky surveys are highly desired to establish the census of merging non-compact stars, a quest that should be feasible for the Milky Way population. Such surveys covering a substantial part of the sky in the IR have already started to operate (e.g., De et al. 2020; Frostig et al. 2022; Sumi et al. 2025) or are expected to be carried out by upcoming space observatories (see Sect. 7).

## Remnants

### The star

**Fig. 10:**
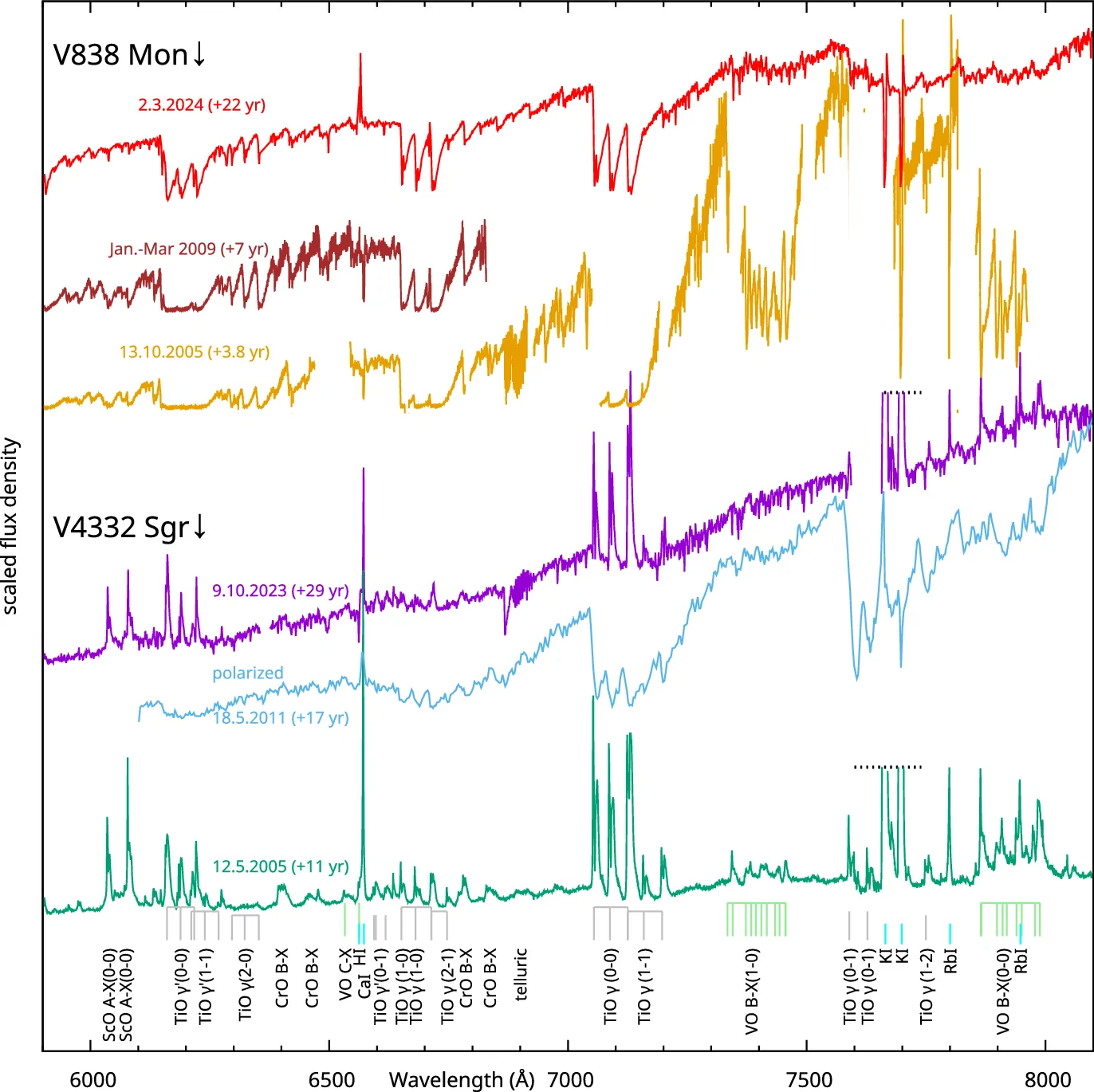
Late evolution of red nova spectra. The top three spectra are of V838 Mon which erupted in 2002 and represents a high-/intermediate-mass red nova remnant (adapted from Kamiński et al. 2009; Tylenda et al. 2015, Kamiński, in prep.). Spectra a few years after the eruption show very deep molecular absorption bands, often saturated, which become shallower with time (e.g., TiO $\gamma \prime$ 1–0 near 6700 Å). Emission lines have been relatively sparse in V838 Mon. Uncontaminated photospheric lines appeared only recently. The bottom three spectra are of V4332 Sgr, which erupted in 1994 and is used here as a template for spectral evolution of low-mass remnants (spectra from Tylenda et al. 2015; Kamiński and Tylenda 2013, Kamiński,inprep.). The 2005 and 2023 spectra are dominated by circumstellar emission in neutral atoms and molecules, and only recently have a weak photospheric component. Strong emission in the K I doublet and severe telluric features were blanked for clarity. The 2011 spectrum shows polarized flux, representing the much purer photospheric spectrum of spectral type ∼ M5, similar to that of V838 Mon. Main spectral features are labeled at the bottom of the plot (cyan markers for atomic features, green for VO, and grey for TiO)

While the circumstellar remnants have been observed extensively, photospheric spectra of only two central stars—V838 Mon and V4332 Sgr—have been obtained so far. Their most recent evolution is shown in Fig. 10. The photospheric spectrum of V838 Mon, even though it was significantly contaminated by circumstellar features, became recognizable shortly after the eruption and has been studied extensively at optical and NIR wavelengths. In the case of V4332 Sgr, whose central star is strongly blocked by a dusty disk, the spectrum is visible due to scattering on dust above and below the plane of the disk, and is most clearly seen in polarized light (Fig. 10; Kamiński and Tylenda 2013). The Ca ii triplet in absorption is likely the first strong photospheric feature to reappear after the eruption. At later times, the spectra of the stellar remnants show cool, $T_\textrm{eff}$=3200–3700 K, photospheres (spectral types M3–M5) of low gravity. These objects are therefore close to the Hayashi limit. While temperatures are relatively easy to determine, owing to the presence of molecular bands of TiO and VO, the spectra acquired so far have been of insufficient quality for a direct measurement of logg. With well-constrained temperature and distance to V838 Mon, the radius of the star is estimated at 464 $\hbox {R}_{\odot }$ (Kamiński et al. 2021b). Recent observations show that, at least in the case of V838 Mon and V4332 Sgr, the obscuring material clears out from the line of sight, and deep high-resolution observations should be possible in the near future, offering the prospect of improved characterization of both photospheres.

SED modeling of the remnants also provides constraints on the effective temperatures of the stars, but is generally much less accurate than direct spectral typing (as it is degenerate with respect to reddening and assumed dust properties). A few years after the outburst, central remnants are found at temperatures from 2200 K (e.g., AT2021blu and AT2021biy; Karambelkar et al. 2026) to 4000 K (Steinmetz et al. 2025; Reguitti et al. 2026a). Merger remnants are typically M-type stars.

Because of their very extended envelopes, freshly coalesced stars are expected to have convective atmospheres typical of ordinary red (super-)giants. Convective cells on the surface can explain their erratic post-outburst photometric variability (e.g., Liimets et al. 2023) and relatively large macroturbulence velocities (priv. comm.). Kamiński et al. (2023) advocated that a strong signature of lithium in the spectrum of V838 Mon is a consequence of strong convective mixing in the envelope of this star, but it remains unclear whether this is a common feature among the red nova remnants.

It is worth noting that the products of mergers in the extremely massive red novae, with progenitor masses above, say, 50 $\textrm{M}_{\odot }$, may be much hotter stars. In the case of SN Hunt 248 with a yellow hypergiant progenitor of 60 $\textrm{M}_{\odot }$, the remnant is thought to be of spectral type B (Mauerhan et al. 2018). The identification of the object as a red nova is, however, still questionable.

***Winds***

Having gigantic sizes and cool photospheres, the stellar remnants of red novae should be prone to developing dust-driven winds similar to those of red supergiants and of AGB stars. Because the higher-mass remnants (like V838 Mon and many extragalactic red novae) remain very luminous even after the eruption has ended, there is a possibility that additional momentum is absorbed by gas through broad molecular absorption bands. A long-lasting wind is certainly observed in V838 Mon through optical absorption features (Kamiński et al. 2023) and was spatially resolved at millimeter wavelengths (Kamiński et al. 2021b). The wind is clumpy and has the mass-loss rate of roughly $10^{-4}$ $\hbox {M}_{\odot }$ $\hbox {year}^{-1}$ (Tylenda et al. 2009), rivaling the most extreme cases of mass loss in red supergiants. The similarity of the V838 Mon wind to that of extreme red supergiants is striking, particularly in that the wind powers asymmetric SiO masers at 43 and 86 GHz (Deguchi et al. 2005; Ortiz-León et al. 2020). Stellar winds may be one way the coalesced star gets rid of excess angular momentum and may significantly shorten the relaxation phase.

***Spin and magnetic fields***

Unfortunately, no magnetic field or rotation measurements are available for red novae or their remnants. The obscuration by dust and veiling of the spectra by circumstellar gas makes it difficult to measure these properties using well-known techniques, which are difficult to apply even to standard field giants (cf. Ma et al. 2024). Note that rotation and surface B fields are challenging to measure once the star is expanded to hundreds of solar radii and has strong convective motions at the surface. A general expectation and theoretical models seem to agree that young merger products should be rapid rotators and have enhanced magnetic fields for a wide range of masses of the progenitor components and a wide range of collision parameters (Wickramasinghe et al. 2014; Frost et al. 2024; Schneider et al. 2019, see also Sect. 6.4). Rotation and magnetic fields are also expected to strongly influence the evolution of the remnant after the thermal relaxation phase (Sills et al. 2001).

***Pulsations***

Immediate merger products are expected to be prone to pulsation instabilities. V838 Mon, the brightest of the known remnants, has recently displayed variability and hydrogen emission interpreted as driven by pulsation shocks (Kamiński et al. 2026). Presently, no solid evidence of pulsations in other red nova remnants exists. Some theoretical work has been done to analyze pulsations in fully relaxed merger products (Rui and Fuller 2021; Henneco et al. 2024a, 2025), but not during the relaxation phase. There is a common belief that the merger event should trigger non-radial pulsations in the coalesced remnant that can distinguish a merger product from stars after mass transfer or accretion (Welty and Ramsey 1994).

***Post-outburst relaxation phase of the star***

In the merger interpretation of red novae, the extended cool remnants are a natural consequence of the fast accretion and energy deposition within the primary (cf. Tylenda 2005; Hoadley et al. 2020; Lipunov et al. 2017). The accumulated gravitational energy is an extra luminosity source that makes the stars brighter than their progenitors. The relaxation to a stable configuration is expected to proceed on the thermal timescale, but detailed calculations for individual cases are missing. However, the post-outburst evolution of red nova stellar products on the currently accessible timescales of decades to centuries is expected to be slow. Indeed, while dynamical changes are observed in the circumstellar environments of red nova remnants, no drastic changes are observed in the stars (e.g., Mobeen et al. 2024; Geballe et al. 2025). Only slow, gradual increases in flux are observed in V4332 Sgr and V838 Mon (Tylenda et al. 2015; Liimets et al. 2023, Kamiński in prep.). Occasional sudden dips in the light curve have been reported and are thought to arise from circumstellar material moving across the line of sight, infalling gas, or possibly shock interactions with the surrounding medium (Tylenda et al. 2015; Lau et al. 2025; Cai et al. 2022b; Goranskij et al. 2018, 2020, see also Sect. 6.2).

While the stellar photosphere appears to be changing slowly, IR observations of the continuum source often show a steady expansion at velocities of 100–300 $\text {km s}^{-1}$. This is most likely due to the expanding dusty ejecta, which remains optically thick at IR wavelengths even over a decade past the eruption (Steinmetz et al. 2025; Karambelkar et al. 2026), reaching radii of the order of $10^{16}$ cm (1000 au). With time, however, this dusty photosphere also becomes transparent.

Eventually (e.g., after the thermal timescale of ∼ 500 years in the case of V838 Mon, assuming its current parameters in Kamiński et al. 2021b), the coalesced star should achieve a new stable configuration that appears younger than the progenitor primary. For instance, in the context of the cluster surrounding V838 Mon and given its most likely mass of 8 $\textrm{M}_{\odot }$, the object should eventually become a blue straggler. V1309 Sco, in turn, is expected to evolve into a red giant (Stȩpień 2011), rather than a blue straggler. Earlier suggestions of an evolution toward a blue straggler were based on a misinterpretation of the available data [see the discussion in Steinmetz et al. (2024)]. An interesting analog of red nova descendants on a time scale of thousands of years may be the suspected merger products, the Blue Ring Nebula (Hoadley et al. 2020) and HP Tau (Reipurth et al. 2026). This topic is discussed further in Sect. 6.4.

### Geometry of the circumstellar medium

The circumstellar structure of red nova remnants is highly complex. Observations and merger simulations show material that was dispersed before the merger, during the merger burst, and, in some cases, after the collision during the ‘remnant’ phase (e.g., winds from the coalesced star or fallback material). These components are thought to interact, often giving rise to shocks. The circumstellar components can take the form of disks (tori or spiral arms), structured bipolar outflows (including jets), and spherical or irregular ejecta. For most known red nova remnants, these components remain spatially unresolved. Imaging observations in which these structures are partially resolved are limited to 2–5 nearby red novae. Most of what we know about the remnants’ architecture thus comes from unresolved observations. These studies demonstrate that there is no single blueprint that explains observations of all known remnants, but some commonalities can be identified. Below, we present two predominant types of architectures discussed in the literature and based on observations of the remnants decades after the red nova event. Merger simulations, which we do not cover here, explore an even wider range of cases but are reviewed elsewhere (e.g., Schneider et al. 2020).

#### Low-mass remnants

**Fig. 11:**
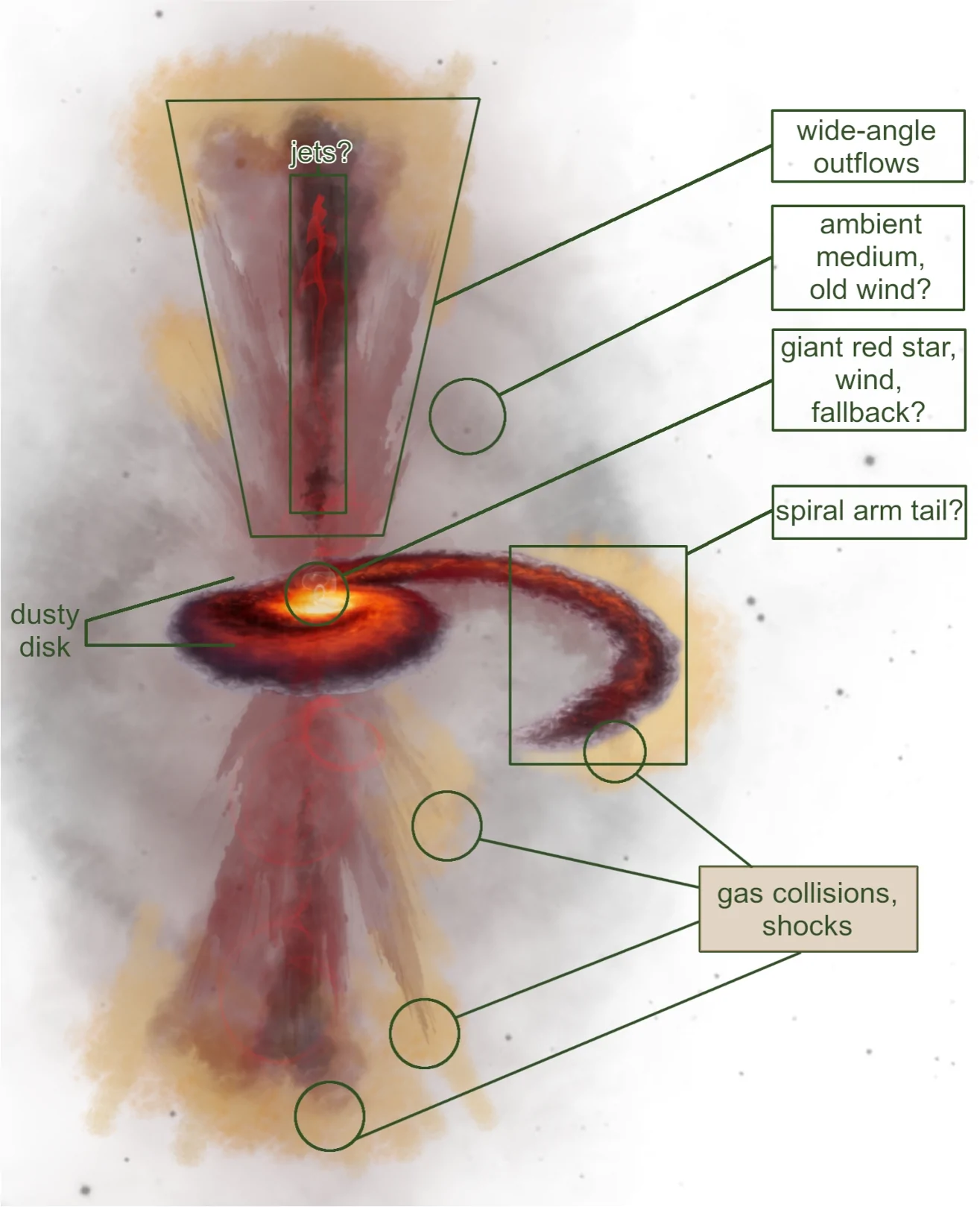
Schematic representation of a low-mass red nova remnant with a disk. The depicted features are not to scale. The scheme reproduces observations of V1309 Sco and V4332 Sgr

The first type of remnant structure we describe is based on observations of the Galactic red nova remnants of V1309 Sco and V4332 Sgr (and to some degree on the CK Vul case). They are both thought to be products of mergers of solar mass stars; V1309 Sco was certainly a contact binary of two sub-giants whose orbit was orthogonal to the sky plane (Stȩpień 2011; Pejcha et al. 2017). These properties are likely to determine the final remnant structure. Our schematic representation of this type of remnant is shown in Fig. 11. Since distances to these objects are poorly constrained, the spatial scales (and masses) we provide below should be regarded as very rough first estimates.

***Disk***

The bloated, coalesced star (Sect. 6.1) is surrounded by a dusty disk with dust temperatures of 200–950 K (Kamiński et al. 2010); the temperatures might reach the dust sublimation temperature (≈ 1700 K) at the inner rim (Banerjee et al. 2015). This range of temperatures is typical for protostellar disks with radial extents of roughly 7–300 au, assuming a central source temperature of 2000 K and stellar radius of 300 $\textrm{R}_{\odot }$. The presence of the dusty disk in red nova remnants is evidenced by detailed analysis of the SEDs of the three mentioned remnants and by other more subtle arguments (e.g., Kamiński et al. 2010; Kamiński and Tylenda 2013). In all three objects, the disk is observed nearly edge-on. Presently, it is uncertain if these are the same disks that were formed during the precursor phase—i.e., a few months to years before the mergeburst, as observed in the light curve V1309 Sco (Tylenda et al. 2011a; Pejcha et al. 2017, see E in Fig. 1)—or whether they formed even earlier.

The dusty disks in red novae must be relatively dense, as that of V4332 Sgr shows deep silicate absorption features and a water-ice band near 3 μm (e.g., Nicholls et al. 2013; Banerjee et al. 2004b). The icy mantles normally form only in dense environments on timescales of Myr. The disk (or parts of it) may therefore largely predate the merger and inspiral phases. Pejcha et al. (2017) calculated that the total mass (dust and gas) of the disk formed just before the mergeburst of V1309 Sco, a feature dubbed ‘the death spiral outflow’, was 0.05 $\textrm{M}_{\odot }$ (see also Zhu et al. 2013). Based on data presented for V4332 Sgr in (Kamiński et al. 2010), our estimates suggest a similar disk total mass of 0.06 $\textrm{M}_{\odot }$. For the nearly edge-on viewing angles, the disks contribute strongly to polarized flux in the optical continuum (Sect. 3.4).

While theoretical studies suggest the formation of spiral-like extensions or disk tails during the merger process (cf. Fig. 11; Nandez et al. 2014; MacLeod et al. 2018a, b), there is currently limited observational evidence for such structures persisting in merger remnants decades after the event (but see Mason and Shore 2022). Numerous merger or common-envelope simulations predict the formation of a stable (i.e., bound) disk where most of the angular momentum of the former binary system is stored (e.g., Nandez et al. 2014; MacLeod and Loeb 2020a). Unfortunately, no measurements of angular momentum have been possible in the observed disks. Some authors also suggested that a small portion of the dispersed but bound matter may be falling back on the central star long after the merger (e.g., Lau et al. 2025), but solid observational support of this notion is still missing for stellar merger remnants. On a longer timescale, it was proposed that post-merger disks can eventually form Jupiter-mass planets (Martin et al. 2011).

***Bipolar outflows***

The disk is associated with a pair of conical outflows expanding at speeds of 200–300 $\text {km s}^{-1}$. A spectropolarimetry experiment demonstrated that these lobes are composed of both dusty and cool, mostly neutral, gas (Kamiński and Tylenda 2013). The dust grains scatter the stellar light from high above the disk plane at scattering angles close to $90^{\circ }$, introducing strong polarization. The gas component is also seen in fluorescent emission from these regions owing to efficient scattering and includes strong optical resonance doublets of K i and Na i, and molecular bands such as TiO and VO (Kamiński and Tylenda 2013; Tylenda et al. 2015). The molecular bands suggest gas temperatures from 100 K to a few hundred K. Even cooler regions of the bipolar lobes (at 30–100 K) are seen in molecular emission at (sub-)millimeter wavelengths and have been partially resolved in V4332 Sgr by ALMA (Kamiński et al. 2021b). The ALMA interferometric observations revealed a bipolar outflow with maximal deprojected velocities of about 300 $\text {km s}^{-1}$. The velocity field was well reproduced, with gas speeds being proportional to the radial distance from the star. The radial extent of the lobes was about 600 au in 2016, consistent with the material being ejected in the 1994 eruption of V4332 Sgr. A similar circumstellar component, albeit spatially unresolved, is observed in V1309 Sco at optical-IR to mm wavelengths (Steinmetz et al. 2024).

Most of the observed characteristics of the outflows are consistent with the scenario proposed by Pejcha et al. (2017) and MacLeod et al. (2018a), whereby the expanding merger ejecta, originally in the form of a spherical or equatorial outflow, is deflected by a pre-existing spiral disk (or torus) into two opposing conical structures. On the one hand, future observations of the bipolar lobes should be easier as the material expands, but on the other hand, it will be more challenging to detect the outflows because the cooling material gets dimmer in emission lines.

No highly collimated jets have been reported for the discussed remnant type (except for CK Vul, possibly due to a companion, see Kamiński et al. 2021a), likely due to the limited spatial resolution of current observations. In spatially unresolved observations of V1309 Sco, shock tracers such as $\hbox {H}_2$ NIR emission or mm-wave emission of $\hbox {HCO}^+$ occur only in the blueshifted and redshifted components, respectively, suggesting that the bipolar lobes are not identical. This is consistent with many merger and common-envelope simulations that highlight the rise of inhomogeneities in the dispersed matter (e.g., Nandez et al. 2014), including asymmetries in the bipolar component (e.g., Frank et al. 2018).

***Ambient medium***

The mentioned shocks can also be interpreted as a signpost of an ambient medium into which the deflected merger ejecta expands. It is very likely that the medium was formed by relic mass loss from the progenitor occurring hundreds of years earlier, possibly by tenuous winds or some sort of precursor mass loss preceding the ‘death spiral outflow’. Some post-outburst observations of forbidden lines arising from these late interface shocks indicate densities of $10^{4\pm 1}$ $\hbox {cm}^{-3}$ (Steinmetz et al. 2024), but much higher values have also been reported ($10^8$ $\hbox {cm}^{-3}$; Mason and Shore 2022).

The ambient circumbinary medium may also be apparent during the outburst. Narrow absorption lines seen in Balmer Hα and the resonance Na i doublet at fixed velocities may be a signature of this cool gas along the line of sight and expanding at terminal velocities that are lower than those characterizing the mergeburst ejecta (e.g., 60 $\text {km s}^{-1}$ in V1309 Sco, Mason and Shore 2022; Mccollum et al. 2014, see also Sect. 3.2). This ambient medium is first affected only by the red nova radiation, triggering feedback fluorescent emission. It is later excited in the red nova remnant by collisions with the ejecta. In V1309 Sco this ambient medium extends out to < 3000 au and may have an ellipsoidal distribution (Mason and Shore 2022).

Observations indicate that there is an even more extended and thus older circumbinary component in the low-mass remnants. In studies which consider photometric fluxes extending to FIR and submm wavelengths, a cool (≈ 30 K) dust component is identified at radii of a few thousand au (Tylenda and Kamiński 2016; Steinmetz et al. 2025). These spatial scales are comparable to the Oort cloud. It remains unexplored how red nova outbursts affect these systems’ frontiers, but dust heating and even photo-desorption of ices should be considered (Kaminski 2010).

#### High-mass remnants

**Fig. 12:**
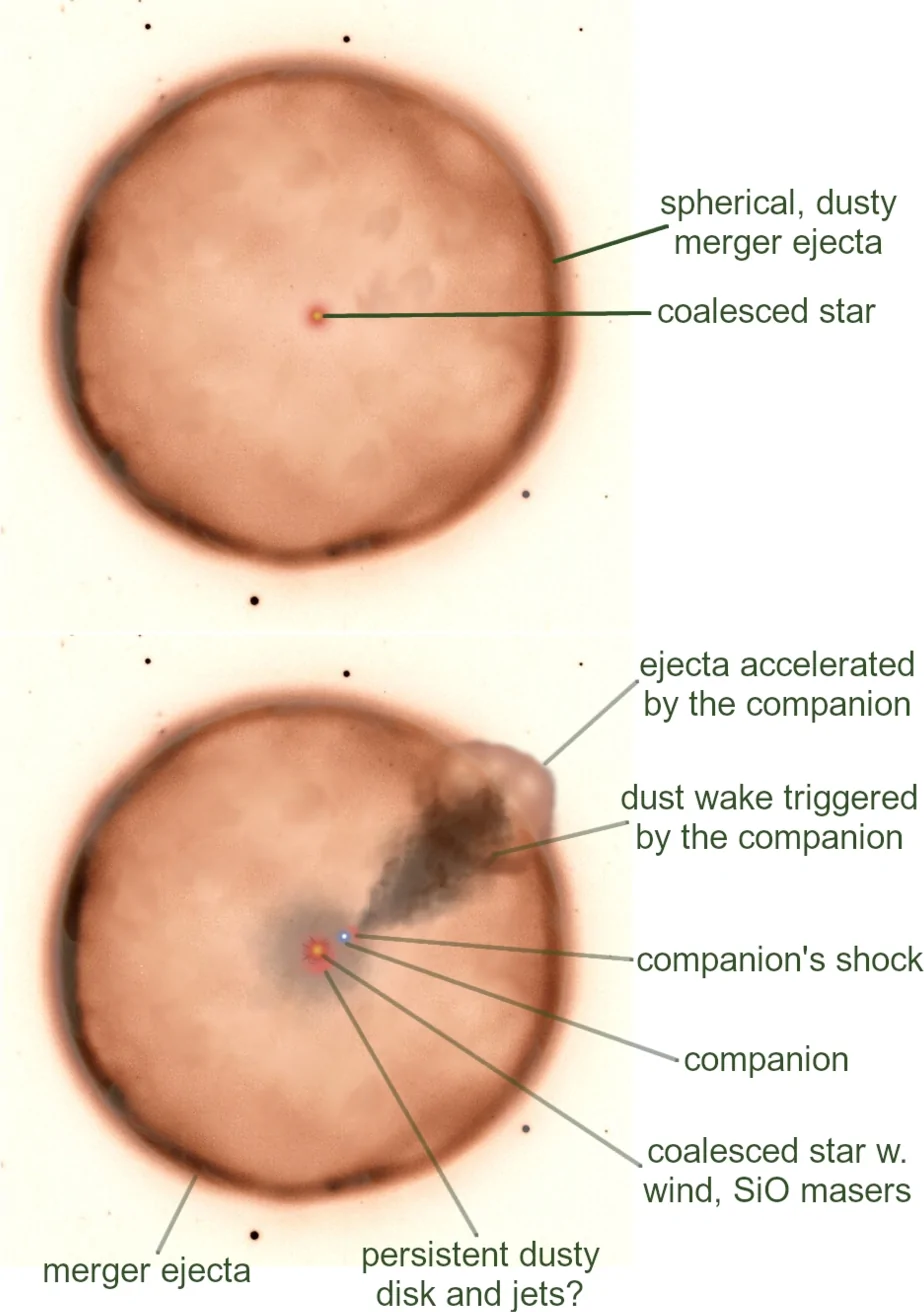
Schematic representations of ‘high-mass red nova remnants‘. The top panel shows a remnant with no companion, while the bottom presents a case with a companion, as in V838 Mon. The depicted features are not to scale

The extragalactic high-mass red novae, with predominantly YSG progenitors, are difficult to study after the outburst, and only a few objects have been observed years after the end of the eruption. The limited data, usually in the form of SEDs, are interpreted within the assumption that the remnant is dominated by spherically symmetric dusty ejecta (Karambelkar et al. 2026; Reguitti et al. 2026a). Our picture of these remnants is thus basic, as depicted in the top panel of Fig. 12. The ejecta is assumed to be freely expanding with its terminal velocity, *v*, and with radius increasing as *vt*; it is thus reaching radii of a few hundred au in the first few years of the post-merger evolution and often remains optically thick at visual and IR wavelengths. Although late re-brightening of a remnant is interpreted as arising from circumstellar shocks (Cai et al. 2022b), it is unclear which components are involved. So far, no observations support the presence of disks or collimated outflows, such as jets, among the red novae with massive primaries.

However, since the Galactic red nova V838 Mon has a progenitor with a mass of ≈ 8 $\textrm{M}_{\odot }$ and its luminosity ($10^6$ $\textrm{L}_{\odot }$) approached those of the extragalactic events, it may be treated as a nearby analog of these high-mass cases. Still, having a young (MS or pre-MS) progenitor, V838 Mon may be a special case among the analyzed red novae, but for illustrative purposes, we will treat this well-studied object as a possible template for the extragalactic population. The remnant structure is schematically shown in the lower panel of Fig. 12.

The central object is a cool, low-gravity star whose luminosity exceeds that of the progenitor. This may result in the star developing a wind similar to that of red supergiants (cf. Sect. 6.1). The high luminosity and dusty wind are also prerequisites for activation of SiO masers. As the remnant evolves, the wind occupies an increasingly large volume, but its terminal velocity and mass-loss rates are much slower than the ejecta. For V838 Mon, the wind speed is about 50 $\text {km s}^{-1}$ while the ejecta velocities were reaching 300 $\text {km s}^{-1}$ (Kamiński et al. 2023); the current mass-loss rate is at least three orders of magnitude lower than during the mergeburst (Tylenda et al. 2011b).

In addition to the dusty wind, the coalesced star in V838 Mon is surrounded by a flattened, dusty structure, which is apparent in NIR-MIR interferometric data at radii of ≲ 10 au (Mobeen et al. 2021, 2024). While this structure is persistent, its exact form and origin remain uncertain (Zain Mobeen et al. 2025). It is therefore unclear if we should expect its analogs in extragalactic remnants.

Interferometric imaging of the ejecta in V838 Mon by ALMA showed that the bulk of the gas lost during the merger has the form of a spherical shell (Kamiński et al. 2021b). It is only distorted by the presence of a companion, which interacted with the passing ejecta and the post-outburst wind. As argued in Sect. 4.4, mergers in triples should not be uncommon among the extragalactic population of red novae. The influence of the companion is twofold: on the shape of the ejecta and its composition. Ejecta passing the companion can be accelerated within a narrow cone. Radiation from the companion can photoionize the passing matter. Additionally, a shock may form upstream of the companion, changing the molecular composition of the matter and triggering more efficient dust formation, similarly to the case in binary AGB stars (Danilovich et al. 2025). This mechanism can significantly increase the dust production of red novae. All the effects are illustrated in Fig. 12 and described in more detail in Kamiński et al. (2021b).

Since extragalactic objects dominate the known sample of red novae, it is important to verify if any of the features known from V838 Mon are common and confront them with theoretical models of these more massive red novae.

### Composition of the circumstellar matter

Most red nova remnants with non-compact progenitors do not show any compositional anomalies that would be common for the entire class, except, perhaps, for enhanced lithium abundances in many of them (see below). In the merger interpretation of red novae, the composition of the remnant and dispersed matter is directly linked to the composition of the progenitor binary components. Some simulations of off-axis (parabolic) collisions show that the material from the secondary is well mixed with that of the primary in the outer parts of the coalesced object (Tylenda and Soker 2006; Sills et al. 2001). The remnant’s surface composition may thus be a mixture of both stars.

***Elemental composition***

Except for CK Vul, which is a special case discussed below, no systematic anomalies in the elemental composition of red novae have been identified. Early claims of overabundances of *s*-process elements in the outburst spectra of V838 Mon should be taken with caution, as they were based on methods tailored to chemical analysis of quiescent stars (Lynch et al. 2004; Kipper and Škoda 2007). Most of the remnants seem to have slightly subsolar metallicity, consistent with an old age (e.g., 8 Gyr for V1309 Sco) or a location in the outer Galaxy (V838 Mon is in the Outer Arm; Kipper and Škoda 2007). A persistent presence of the λ6707 optical resonance line of lithium in the spectra of three red novae led Kamiński et al. (2023) to speculate that the remnants are Li enhanced due to the activation of the Cameron-Fowler mechanisms in the convective exteriors or due to other mixing mechanisms. It is uncertain if the same would happen for mergers involving stars more massive than those of the Galactic red nova. While an intrinsic origin of Li in red nova remnants is not the only possible explanation of the observed lines (see Kamiński et al. 2023, for further discussion), if the Li enhancement is confirmed to be merger-produced, red novae could represent an additional source of lithium in the Galaxy, alongside classical novae (Della Valle and Izzo 2020; Molaro 2023).

***Molecules***

Although molecules play an important role in identifying and characterizing red novae shortly after outburst and over the subsequent decades, only a few studies have attempted to derive molecular abundances in the atmospheres (Lynch et al. 2004, 2007) and circumstellar media (Kamiński et al. 2018a, 2021b; Karambelkar et al. 2026) of these objects. Molecules observed at optical, IR, and submillimeter wavelengths include primarily^2^: oxides CO, $\hbox {CO}_2$, AlO, CrO, VO, TiO, SO, $\hbox {SO}_2$, NO, SiO, ScO, YO, OH, $\hbox {H}_2$O, and AlOH; and sulfur-bearing species—SH, SiS, and $\hbox {H}_2$S—all commonly observed in oxygen-rich stars, old and young. While TiO and VO are the most recognizable features of late red nova spectra in the optical, CO and $\hbox {H}_2$O dominate at longer wavelengths and can be identified even in low-resolution, low-S/N spectra (Banerjee and Ashok 2002). Reliable estimates of circumstellar gas column densities remain very sparse. While local thermodynamic equilibrium is assumed to calculate partition functions, the molecules observed at IR wavelengths are always excited by the star’s radiation and by dust layers at different radiation temperatures, making such estimates unreliable. Absorption features and submillimeter-wave spectroscopy yield dependable results.

Currently, there is no quantitative evidence for anomalous molecular abundances, but researchers have noticed the prevalence of optical and IR bands of AlO, CrO, or $\hbox {PH}_3$, which are rarely observed (AlO) or have not been observed elsewhere (CrO; Banerjee et al. 2003; Kamiński et al. 2015a; Steinmetz et al. 2024; Lau et al. 2025). These rare spectral features may be caused by special temporary excitation conditions, and not necessarily by the overabundance of the species or their parent metals. However, this notion requires a quantitative verification. Finally, it should be noted that some molecular species observed near V838 Mon, including AlOH, $\hbox {SO}_2$, and $\hbox {H}_2$S, were formed in shocks due to the interaction of the merger ejecta with the companion (Kamiński et al. 2021b). Thus, the observed molecular inventories likely reflect a combination of environmental factors, including interactions with the outer tertiary star.

Spectra of extragalactic red novae usually have modest S/N, allowing only the most prominent absorption bands to be studied: TiO and VO in the optical (e.g., Pastorello et al. 2021a), CO in the NIR, and $\hbox {H}_2$O in the NIR and MIR (Blagorodnova et al. 2021; Cai et al. 2022b; Karambelkar et al. 2023, 2025, Gomez-Muñoz in prep.). Water bands are often so pronounced that they need to be considered in SED modeling of late remnants (Karambelkar et al. 2026; Reguitti et al. 2026b).

***Isotopes***

The presence of molecular features in red nova spectra has made it possible to place the first observational constraints on the isotopic composition of the material in the circumstellar environment of the remnant. The best studied in this context is V838 Mon, whose IR bands of CO imply a ^12^C/^13^C ratio of 10–100 (Geballe et al. 2002) and submillimeter-wave emission yields ^12^C/^13^C>80, all consistent, within uncertainties, with the interstellar medium value. Furthermore, Kamiński et al. (2009) analyzed the main TiO isotopologues in the optical spectrum of V838 Mon, that is ^46^$\hbox {Ti}^{16}$O, ^47^$\hbox {Ti}^{16}$O, and ^48^$\hbox {Ti}^{16}$O, but found no deviation from the solar titanium isotopic ratios. The only other red nova remnant where isotopic measurement was attempted is V4332 Sgr. Banerjee et al. (2026) found ^12^CO/^13^CO≈3 which may imply merger-triggered mixing. Banerjee et al. (2004a) put an upper limit of 10 on the ^27^Al/^26^Al ratio from optical electronic bands of AlO, consistent with no significant enhancement with the radioactive isotope of ^26^Al. The case of CK Vul is discussed in a separate section below.

***Solids***

Dust spectral features have been studied mainly in the remnants, although there are a few observations of IR dust excess in progenitors (Tylenda et al. 2013) and in outbursting red novae (Mccollum et al. 2014; Tylenda et al. 2011a, 2013; Blagorodnova et al. 2020). The dominant presence of oxides in the majority of the remnants is associated with the predominance of inorganic (noncarbonaceous) dust in their circumstellar media.

Most red novae were observed to start forming dust days to months after the outburst (e.g., Tylenda 2005; Tylenda and Kamiński 2016; Mccollum et al. 2014; Wisniewski et al. 2008; Blagorodnova et al. 2020; Wavasseur et al. 2026), giving rise to a broad and often structured 10 μm feature. It is most often observed in absorption, but in some cases, the emission component may dominate even a decade after the outburst (e.g., in V838 Mon; Lynch et al. 2004; AT2018bwo; Karambelkar et al. 2026). The 10 μm is identified as arising from a mixture of silicates (e.g., pyroxene and olivine, or the generic ‘astronomical silicates’^3^) (Nicholls et al. 2013; Woodward et al. 2021; Steinmetz et al. 2025) but some studies emphasize a significant contribution from amorphous alumina dust, especially early (years) after the eruption (Banerjee et al. 2015; Woodward et al. 2021; Steinmetz et al. 2025). In a few cases, admixture of glassy olivines was necessary to reproduce SEDs of the remnants (Steinmetz et al. 2025; Karambelkar et al. 2026). FeO and MgO dust and other solid oxides or pure iron grains have also been suggested (Banerjee et al. 2007, 2015; Zhu et al. 2013).

The dust in red novae is thought to evolve on the timescales of decades, with signs of progressing annealing and an increasing level of oxidation (Banerjee et al. 2015). The diminishing contribution of the more refractory alumina being associated with the increase of silicates (Banerjee et al. 2015; Woodward et al. 2021) seems to be in line with the classical scenario of inhomogeneous dust nucleation in oxygen-rich circumstellar media, where silicate dust grows on alumina condensation cores (Gail and Sedlmayr 1998).

It is generally predicted that the mass of dust increases with the age of the red nova remnant, but there are so far only sparse observational constraints supporting this hypothesis (Blagorodnova et al. 2020; Karambelkar et al. 2026; Wavasseur et al. 2026, Gomez-Muñoz in prep.). Solid water ice was found only in V4332 Sgr (Banerjee et al. 2004b), but its origin is unknown, as there is no direct indication that it formed after the eruption, and it may be a trace of pre-existing material (cf. Mccollum et al. 2014; Tylenda and Kamiński 2016). Since solids play a great role in shaping the spectral energy distribution of red novae in their post-outburst evolution (and perhaps shortly before it), dust generally plays a major role in understanding red novae and mergers of non-compact stars. A vast amount of theoretical work has been invested in understanding dust formation in the context of non-compact mergers (e.g., MacLeod et al. 2022) and in common-envelope ejection events (e.g., Iaconi et al. 2020; Bermúdez-Bustamante et al. 2024; Mu et al. 2026), but the dust detailed composition, depending on the binary stars and the condensation sequence preceding the outburst remains largely unknown.

The terminal dust mass yields were derived for a handful of remnants that are bright enough in the IR to be studied years to decades after the eruption. They range from $10^{-5}$ to $10^{-2}$ $\textrm{M}_{\odot }$ (see Col. 10 in Table 1) (Karambelkar et al. 2026; Reguitti et al. 2026a). The one well-known planet engulfment event resulted in $10^{-11}$ $\textrm{M}_{\odot }$ of dust (De et al. 2023), the lowest value ever reported for a red nova. The observations confirm the prediction that slow mergers, involving stars with extended atmospheres, produce much dustier remnants (MacLeod et al. 2022; Steinmetz et al. 2025). Still, there are also hints that the red novae with more massive progenitors produce more net dust mass than the low-mass counterparts.

Karambelkar et al. (2026) analyzed the dust production budget of red nova remnants and proposed that, eventually, they contribute at least 25% of dust mass estimated for core-collapse supernovae, which makes them important dust polluters on Galactic scales. This result accounts for the more frequent low-mass progenitors, which were assumed to be less efficient at dust production, and the more massive analogs, in which late interactions between ejecta and the circumstellar medium can considerably enhance the dust mass. Since our inventory of red novae likely misses the most dusty—and thus IR-only—transients, the role of red novae on dust production is probably still underestimated. We also note that the mass dust estimates should distinguish the ejecta component that would eventually disperse into the ISM from a gravitationally bound component that may be present around the remnant in a disk or torus and thus never reach the ISM.

***Exceptional CK Vul***

Most of the above characteristics are not valid for the case of the CK Vul remnant. Compared with the solar elemental composition, it is significantly enriched in He, Li, and N, but is depleted in H and O (Kamiński et al. 2017; Tylenda et al. 2019; Hajduk et al. 2013). Moreover, the ratio of O/C may change across the remnant, with the closest observable vicinity of the stellar remnant being oxygen-rich (O>C) (Kamiński et al. 2020). The molecular composition, explored mainly through (sub-)millimeter transitions, is also very extraordinary, as it is a combination of species typically only observed in oxygen-rich (e.g., SO and $\hbox {SO}_2$) and carbon-rich environments (e.g., CCH and CS) (Kamiński et al. 2015b, 2017, 2020). In addition, many of the observed species are surprisingly complex, including $\textrm{CH}_{3}$OH and $\textrm{CH}_{3}$ $\hbox {NH}_2$ (Kamiński et al. 2017). Shock-induced infrared emission of the simplest molecule, $\hbox {H}_2$, is also observed (Tylenda et al. 2019; Kamiński et al. 2020).

The current inventory of CK Vul’s molecules includes 28 items, making it the richest red nova remnant in that respect. Many of these species appear in their isotopic variations, indicating highly non-solar isotopic ratios, including ^12^C/^13^C≈4, ^14^N/^15^N≈20, ^16^O/^18^O≈ 36, and ^27^Al/^26^Al≈7. The ratio of the aluminum isotopes was derived from the unprecedented observations of the rotational transition of the AlF molecule containing the radioactive isotope of ^26^Al (Kamiński et al. 2018b). Mid-IR observations of the remnant revealed many spectral features usually assigned to polycyclic aromatic hydrocarbons (PAHs; Evans et al. 2016), which are signatures of UV-irradiated carbonaceous solids, making CK Vul the only remnant with such a clear display of organic dust. The overall chemical composition of CK Vul is explained by the hypothesis that this red nova (Nova 1670) was caused by a merger between a ∼ 2 $\hbox {M}_{\odot }$ red giant branch star and a He white dwarf (WD). The compact WD was able to plunge deep into the interior of the giant, disturbing its own He core surrounded by a layer rich in ^26^Al (Kamiński et al. 2018b). The merger also ignited partial He burning (Kamiński et al. 2017). The observed remnant is a mixture of the nucleosynthesis products of CNO hydrogen burning from the progenitor giant, the partial He burning, and core material dispersed by the violent collision (Tylenda et al. 2024). The major difference between CK Vul and most other red novae is related to the nature of the progenitor binary and the short ignition of helium burning caused directly by the merger. This may be a common characteristic of mergers involving white dwarfs (van der Merwe et al. 2024, and references therein).

### Related objects and post-relaxation analogs of remnants

The red nova remnants will evolve into classes of objects long suspected of being merger products. The expectation is that V838 Mon will become a blue straggler; V1309 Sco, V4332 Sgr, and BLG360 are likely to evolve into red giants, or perhaps FK Com stars, possibly with extraordinarily high rotation and magnetic fields (Stȩpień 2011; Tylenda et al. 2013). Finally, CK Vul is believed to become an early-R type carbon giant (Tylenda et al. 2024). The immediate future of extragalactic red novae that have YSG progenitors remains largely unexplored, but after some thousands of years, some may become blue supergiant progenitors of core-collapse SNe, like SN 1987A (Podsiadlowski et al. 1992), or progenitors of circumstellar interaction SNe (Chevalier 2012).

Understanding what kinds of stars are produced in red nova bursts is one of the main drivers of the field. Detailed predictions of the long-term evolution of the well-characterized red novae are still missing. Much more theoretical work has focused on the evolution after thermal relaxation, i.e., after the coalesced star returns to hydrostatic configuration, as already discussed in Sect. 6.1. Observationally, we are limited to a small sample of Galactic remnants, most of which are only decades old. There is therefore a strong need to identify more, possibly older, remnants to expand the sample and extend the accessible timescales.

Identifying red novae through their outburst requires photometric and spectroscopic observations. As pointed out in Sect. 2.1, several historic objects were identified as red novae even though their eruptions took place long before the group of transients was defined (i.e., shortly after 2002). These include M31 RV (eruption 1998), V4332 Sgr (Nova 1994), and CK Vul (Nova 1670). With these successful cases, it was believed that many other overlooked historic red novae could be found, even in cases when the exact coordinates of the transient were not well known (Kimeswenger 2007). One expectation based on these characteristics of the historic transients is that they leave red, dusty, or otherwise peculiar remnants that can be distinguished from field stars. One interesting example of a search for an overlooked red nova is Nova 1943 (Mayall 1949), a.k.a. V1148 Sgr. It was reported to exhibit a late-type spectrum with strong Ca ii absorption lines and possibly absorption bands of TiO, unusual for classical novae. Despite meticulous searching, its remnant has not been unambiguously identified (Bond et al. 2022).

**Fig. 13:**
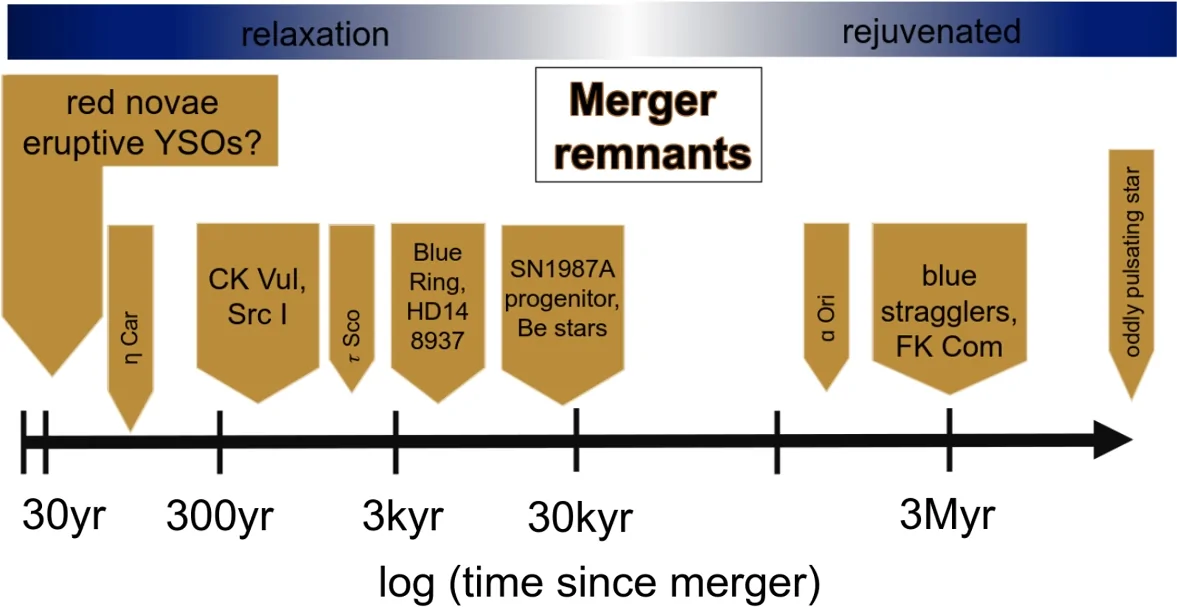
Examples of postulated merger remnants on the time axis since the presumed merger. It is unclear into which objects red nova remnants will evolve on the presented timescales. The objects shown represent an illustrative selection of proposed merger remnants whose merger eruptions, had they been observed, would not necessarily have appeared as red novae

The search for remnants of historic outbursts continues (Kamiński et al. 2022; Bond et al. 2022; Bond 2018), but is realistically limited by observational records that extend back a few hundred years, at best (e.g., Yang et al. 2025). For much older red novae, we may not have a direct record of the outburst, which makes it difficult to formally classify them as red novae. In the astronomical literature, there are, however, hundreds of claims of objects being merger products from thousands to millions of years ago. Some examples are compared on the time axis in Fig. 13. Below, we mention only a few cases that seem most relevant to the red nova research.

One possible class of older merger remnants (relative to the currently observed sample of red novae) is represented by blue stragglers. V838 Mon, which is a product of a merger involving a B-type MS star, is predicted to contract into a rejuvenated B dwarf in the Kelvin–Helmholtz time scale of ∼500 year (using parameters from Kamiński et al. 2021b). As a member of a stellar cluster, V838 Mon is expected to evolve into a blue straggler. Most known blue straggler stars are likely to be significantly older. Only a fraction of blue stragglers are merger products of MS stars, and these are often difficult to distinguish from post-mass transfer systems (for a recent review, see Wang and Ryu 2026). Ongoing studies of blue stragglers’ rotation, surface magnetic fields, and elemental composition are a promising way to understand red novae and their post-outburst evolution to a hydrostatic configuration. Conversely, tracking the evolution of V838 Mon may be extremely informative for understanding the formation and evolution of blue stragglers.

Merger products can also be identified as binary components that appear much younger than their companions. These are often cases of mergers in hierarchical triples (see Sect. 4.4). The claimed merger products are likely to be puffed-up and highly magnetic stars with rapid rotation. However, angular momentum loss from winds and magnetic braking is expected to slow down the remnant on timescales of tens of Myr, making it a slow rotator compared to other cluster members (see Wang et al. 2022). In some cases, this is associated with anomalies in the surface elemental composition. Examples include HD 148937 (Frost et al. 2024), θ Sco (Lewis et al. 2022), τ Sco (Schneider et al. 2020; Keszthelyi et al. 2021), and some Cepheids (e.g., Espinoza-Arancibia and Pilecki 2025). On similar grounds, a merger scenario has been proposed to explain properties of B and B[e] supergiants (Kraus 2019; Menon et al. 2024), including FS CMa stars (Dvořáková et al. 2024). Perhaps a variation of these types of remnants is the future of V838 Mon remnant or some of the extragalactic red novae.

Much younger are suspected merger remnants in star-forming regions. Here we provide two examples. On the high-mass end, *Source I* (or *SrcI*) in the Orion’s Kleinmann–Low Nebula (Ori KL) is thought to be a possible merger product from ≈ 550 years ago (Bally et al. 2020). A dynamical interaction in a multiple system of several low- and high-mass protostars created the famous outflow of OMC1, and *Source I* is one of the walkaway stars in the complex. The candidate merger remnant is of the mass of 5–25 $\textrm{M}_{\odot }$ and possesses a large disk whose many properties are atypical for normal young stellar objects (Testi et al. 2010; Wright et al. 2024).

Another merger candidate known from protostellar studies is HP Tau/G2 (V1025 Tau) (Reipurth et al. 2026, and references therein). This object, of a mass of about 0.7 $\textrm{M}_{\odot }$, is also a walkaway star moving away from a site where multiple protostars interacted dynamically 3000–5600 years ago. Its current properties include high rotational speed, magnetic activity (at optical, radio, and X-ray regimes), and a rejuvenated appearance compared to other cluster members. These properties are remarkably reminiscent of longer-known suspected merger remnants, FK Com stars. This group of stars has been proposed to be products of mergers of contact W UMa stars (Stȩpień 2025), that is, similar to the progenitor of the red nova V1309 Sco (Stȩpień 2011) (We note, however, that W UMa stars and V1309 Sco were much older than HP Tau/G2 progenitor). Interestingly, objects like HP Tau/G2 do not seem to have dusty disks. If they are direct descendants of systems like the V1309 Sco remnant, the disks must be eroded or dispersed on a timescale of thousands of years.

There is also a group of star-forming regions that have been proposed to be more embedded merger sites than those discussed above, but still closely related to Ori KL. This peculiar group shares certain characteristics with circumstellar media of evolved stars and with star-forming regions. The class includes Ori KL, Sgr B2, W51N, G0.38+0.04, G19.61–0.23, G75.78+0.34, and IRAS 19312+1950 (a.k.a. Deguchi-Nakashima object) (Deguchi et al. 2004; Cordiner et al. 2016; Gong et al. 2026; Zapata 2026, and references therein). All these regions contain components with active SiO masers, which otherwise are never observed in protostellar environments. Note that the red nova remnant V838 Mon is hosting an SiO maser, too (Ortiz-León et al. 2020). Kinematics of these regions indicate some past eruption, perhaps analogous to that in OMC1. Whether it was caused by a red nova-like merger is still being investigated. There are also unique objects seen toward the Galactic center that were proposed to be merger products similar to red nova remnants (Ginsburg et al. 2024; Witzel et al. 2014).

The link of red novae to protostars may be even stronger. Accretion events, similar to FU Ori outbursts, share some characteristics with red novae: the raising slope toward the main peak, a kink in the rising part of the light-curve reminiscent of the pre-eruption in red novae, the main peak and plateau phase in the light curve, spectral appearance during outburst (including spectral type, Balmer lines, the Ca ii triplet, neutral and singly-ionized metals) and after (e.g., TiO and VO absorption), and linewidths (e.g., Hillenbrand et al. 2025; Fischer et al. 2023).

One difference is that classical FU Ori stars have lower outburst amplitudes (2–5 mag) than stellar red novae; they are typically also more embedded and remain longer in the active state, even decades to hundreds of years. The outburst mechanism is also, in principle, different since FU Ori eruptions are triggered by a disk instability, and accretion is fed from a large envelope. Nevertheless, both groups of objects are powered by accretion and shocks. The appearance of optical–IR molecular bands during or after the eruption, including rarely observed species like AlO, is especially intriguing. The commonalities between these two groups of eruptive variables are worth exploring (Guo et al. 2025). There are also related eruptions of young stars of shorter time span than classical FU Ori, which are often compared to red novae (e.g., Lucas et al. 2020) and related to accretion of compact objects, such as protoplanets (Wolf et al. 2024; Guo et al. 2025). These objects may be good analogs of planet-engulfing red novae (De et al. 2023; Kashi and Soker 2017; Kashi et al. 2019).

The famous ‘Great Eruption’ of the luminous blue variable (LBV) η Car is also often compared to red novae. This very massive (≈ 120 $\textrm{M}_{\odot }$) and young (≲3 Myr) binary star dramatically increased its brightness by nearly 9 mag in 1843. This was followed by a prolonged 20-year plateau and 1–2 other peaks of increased brightness. This multi-peak character of the light curve, apart from its timescale, is similar to red novae. The main eruption was also as cool as those of red novae, as the spectrum of the eruption’s light echo indicated a spectral type close to G2–G5 (Rest et al. 2012). Matter was lost at velocities of 100–600 $\text {km s}^{-1}$ (Smith et al. 2018), consistent with what is typically observed in red novae. The remnant of the eruption is the bipolar Homunculus Nebula, whose inner regions are abundant in molecules and dust (Gull et al. 2020), just as in many bipolar red nova remnants. The interpretation of the eruption often leads to the suggestion it was triggered by a massive merger in a triple or higher-order system (e.g., Portegies Zwart and van den Heuvel 2016; Smith et al. 2018). Despite these multiple similarities to red novae, η Car is more classically included in the LBV family of variable stars and was omitted in our Table 1. The red novae with the most massive progenitors, such as SN Hunt 248 or AT1997bs (a.k.a. SN 1994bs), may have even more in common with η Car and P Cyg-like eruptions than with the low-mass red novae (Mauerhan et al. 2018).

While the above examples are all relatively young remnants of presumed mergers at the main sequence or before it, there are also more evolved suspected merger remnants relevant for red novae. Melis (2020) reviewed the relation of dusty RGB stars, Phoenix Giants, to red novae and stellar mergers. These evolutionary-advanced Li-rich stars host dusty disks, which are speculated to be remains of planet engulfment from times when the star entered the first-ascent giant branch and significantly increased its size (Jura 2003; Melis et al. 2009). One such object, TYC 2597-735-1 associated with the Blue Ring Nebula, was proposed to be only 2000–5000 years old (Hoadley et al. 2020). Some of these merger candidates are puffed-up, active, magnetic, and rapidly rotating stars (Günther et al. 2022), and are also hypothesized to be evolving to the FK Com class. The relation of these Phoenix Giants to the red novae with red giant progenitors is still being explored (Steinmetz et al. 2025).

Assuming that the mentioned objects are true remnants of a merger, it is interesting to ask how long one can observe the matter dispersed in a mergeburst or briefly before it. The nebula and disk in TYC 2597-735-1 and the bipolar outflow surrounding HP Tau suggest that the matter can be traced even after ∼5000 years (Hoadley et al. 2020; Reipurth et al. 2026). In HD 148937, a bipolar cocoon is seen 75 000 years after the hypothesized merger (Frost et al. 2024). In SN 1987A, the hourglass nebula with the famous equatorial waist is seen 6650 years after the proposed merger of the progenitor (Brandner et al. 1997). No extended circumstellar matter has been reported for merger-born blue stragglers or FK Com stars, which may be even Myr after the coalescence. Although the survival of merger ejecta depends on its mass, velocity, and surrounding environment, it may remain detectable for up to ∼ 10 000 years after the event. This longevity is encouraging for the future identification of merger products and remnants of red novae.

## Future observational prospects

Progress in the observational study of red novae is likely to be driven along four main directions: the expansion of discovery samples through synoptic optical and infrared surveys, the availability of wide and deep high-quality imaging surveys for the identification of progenitor systems, the development of more realistic multi-dimensional light-curve models of the outbursts, and a shift from treating red novae as rare curiosities to using them as probes of binary evolution. Below, we outline several anticipated advances.

### Red novae in the 2030s

Future advances in the field of observational study of red novae in the next decade will likely be driven by the release of large, multi-wavelength, multi-epoch catalogs from space missions and ground-based surveys and improvements in astronomical instrumentation. Aided by the results of currently operating and future multiplexed spectroscopic surveys—which will provide accurate redshifts for nearby galaxies and detailed characterization of millions of Milky Way stars—deep, wide-field photometric time-domain surveys will have a paved path for the discovery of large numbers of more distant extragalactic red novae and, for the closest events, also characterize their progenitor systems and past variability.

For the nearest Milky Way or Local Group transients, *Gaia* will have its final results released as DR5 in 2030, containing a multi-epoch catalog for the whole duration of the mission. This will enable tracing back the precursor evolution of future LRNe in high-precision photometry and low-resolution spectroscopy. For more distant extragalactic progenitor and precursor studies, the final *Euclid* data release, DR3, in the early 2030s will also provide a deep optical and IR baseline to retrospectively identify the progenitors of upcoming red nova outbursts. Combined multi-epoch, multi-wavelength data on red nova progenitors and precursors offer great potential to shed light on how mass-transfer instability can be triggered in different binary systems, and create an observational benchmark for detailed theoretical studies and simulations.

Discoveries will come in different flavors: the early-time UV will be detected by ULTRASAT (Sagiv et al. 2014), optical photometric data will be delivered by the ongoing Vera Rubin’s LSST (Ivezić et al. 2019; Hambleton et al. 2023), and the cooler, highly dust-obscured transients will be detectable in the IR by the Galactic and High-latitude Wide Field surveys with the Roman Space Telescope (Spergel et al. 2015). This multi-wavelength coverage will be ideal to obtain a complete, long-term characterization of the evolution of thousands of red nova events. For example, LSST is expected to detect 20−1500 new LRNe per year (Howitt et al. 2020; Karambelkar et al. 2023), greatly enhancing the current yearly rate by one to three orders of magnitude.

Despite this promise, the scientific value from these large samples may be limited by the sparse observational cadence in some survey fields and the faintness of the bulk of the discoveries, many of which will remain beyond the reach of operating spectroscopic follow-up facilities. As mentioned in Sect. 2.2, the peak brightness of LRNe is not unique. Other ‘gap transients’ can initially mimic their outbursts, making their identification challenging from photometry alone. Probabilistic classification efforts based on multi-wavelength light curves are ongoing, and they are still the most promising way to photometrically identify different transient types (Boone 2019; Chaini et al. 2026), but these approaches usually require large, simulated or observational light-curve training sets, which are still lacking for LRNe.

In the 2030s, while the increase in the number of discoveries will be meaningful, the most comprehensive physical insights will still likely come from events occurring in the local Universe, for which substantially richer and more detailed datasets will be obtained. Large aperture telescopes such as the ESO Extremely Large Telescope (ELT; Gilmozzi and Spyromilio 2007), Giant Magellan Telescope (GMT; Johns et al. 2012), and, hopefully, Thirty Meter Telescope (TMT; Skidmore and TMT International Science Development Teams, Science Advisory Committee 2015) will start operations in the early 2030 s. The unmatched collecting power of such colossal apertures will enable scientists to obtain high-resolution spectroscopic and spectropolarimetric data on distant extragalactic red novae, allowing detailed comparisons with their Galactic counterparts and studies of the polarization signatures of individual lines. Diffraction-limited, milliarcsecond imaging for a 30 m class optical-IR telescope will also resolve the closest environment to these objects, whether they are clusters of young stars or low-surface brightness nebulae. For red nova remnants, JWST and ALMA will continue to provide high-quality data on the molecular gas and dust that formed in recent and decades-old red-nova remnants. Multiepoch observations of the evolving dusty content of red novae remnants will become a valuable resource to test rapid dust formation models and simulations, and the peculiar chemistry arising in these systems.

Similar to the progress observed for other, more common, transients, the increase in well-characterized events has the potential to reveal different red nova families, associated with different progenitor populations, physical ejection mechanisms, and emission mechanisms. This will offer a unique opportunity to statistically assess the connection of red novae outbursts to common-envelope and merger physics, and observationally constrain the evolutionary pathways predicted by binary population models, and then compare them with the observationally derived rates (Twum et al. 2026).

Looking further ahead to the 2040 s, proposed high-throughput (sub)millimeter facilities such as AtLAST (Orlowski-Scherer et al. 2025) and ALMA2040^4^ could bring red novae into the era of systematic time-domain millimeter astronomy. Their increased sensitivity, broad spectral coverage, and flexible monitoring capabilities would allow dust continuum and molecular-line emission to be followed from the eruption to the cold remnant, constraining dust formation, molecular chemistry, shock excitation, mass-loss kinematics, and the total mass and energy of the ejecta. AtLAST would provide wide-field discovery, long-term monitoring, and sensitivity to extended emission, while ALMA2040 would resolve the ejecta and their compact shock and outflow structures, providing even more details on the geometry of the stellar collisions. Together, these facilities could link the short-lived optical transient to the decades-long chemical and dynamical evolution of stellar merger remnants.

### Gravitational-wave studies of red novae

Optimistically, the red nova research may go beyond the electromagnetic spectrum in the near future. At the moment, gravitational-wave (GW) signals from red nova systems are not detectable with currently operating detectors LIGO-Virgo-Kagra, with peak sensitivity at ∼200 Hz. This is because the radii of the non-degenerate stellar cores are much larger than those of neutron stars or black holes, and the signal would stop at much lower frequencies. Upcoming international space-based gravitational-wave observatories like LISA (Laser Interferometer Space Antenna; Danzmann and LISA Study Team 1997; Amaro-Seoane et al. 2017), with peak sensitivities in the 0.1–100 mHz range, could identify a limited number of these events under strict conditions: the sources must be closer than 1 kpc, and the signal should correspond to the merging compact cores after a failed CEE (rather than a tidal disruption of the companion at a larger radius) (Morán-Fraile et al. 2023; Renzo et al. 2021).

Currently, the most promising landscape for the study of red novae through GWs comes from missions operating in the frequency range between LIGO and LISA, i.e., at 0.1–10 Hz. One example is the Japanese DECIGO (DECi-hertz Interferometer Gravitational wave Observatory; Seto et al. 2001; Kawamura et al. 2011), with its pathfinder mission B-DECIGO planned to launch in the 2030 s, or the Advanced Laser Interferometer Antenna (ALIA) and the Big Bang Observer (BBO) (Crowder and Cornish 2005). Detections of stellar mergers and common-envelope events through gravitational waves will help to uncover the fraction of unseen, likely obscured population of interacting binaries, providing new constraints on current theoretical models. Looking ahead to the post-2030 era, multi-messenger observations of red novae are expected to play a key role in advancing our understanding of binary evolution.

## Data Availability

No datasets were generated or analyzed during the current study.

## Abbreviations

AGB: Asymptotic giant branch
ALMA: Atacama Large Millimeter Array
CE, CEE: Common envelope, common-envelope evolution
CSE: Circumstellar envelope
DDI: Delayed dynamical instability
FIR: Far-infrared
FWHM: Full width at half-maximum
GEE: Grazing envelope evolution
HR: Hertzsprung–Russell (diagram)
ILOT: Intermediate-luminosity optical transients
ILRT: Intermediate-luminosity red transients
IR: Infrared
ISM: Interstellar medium
JWST: James Webb Space Telescope
L2, L3: The second and third Lagrange points, respectively
LBV: Luminous blue variable
LC: Light curve
LRN: Luminous red nova (= red nova)
LTE: Local thermodynamic equilibrium
LSST: Large Synoptic Survey Telescope
MIR: Mid-infrared
MS: Main sequence
MW: Milky Way
NIR: Near-infrared
RLOF: Roche lobe overflow
RGB: Red giant branch
RN: Red nova
RSG: Red supergiant
SED: Spectral energy distribution
SN: Supernova
S/N: Signal-to-noise ratio
SPRITE: ESPecially Red Intermediate-luminosity Transient Event
UV: Ultraviolet
WD: White dwarf
YHG: Yellow hypergiant
YSG: Yellow supergiant
YSO: Young stellar object
ZAMS: Zero-age main sequence
ZTF BTS: Zwicky Transient Facility Bright Transient Survey

## Footnotes

1 Narrow, as compared to other transients, especially SNe.

2 Some identifications made in the literature, e.g., CaH in V838 Mon’s spectrum (Loebman et al. 2015) were incorrect; suggestions of NIR features of CN in extragalactic red novae are ungrounded.

3 Although astronomical silicates have an admixture of carbonaceous dust and some red nova studies use soot or graphite opacities in SED modeling (e.g., Blagorodnova et al. 2021; Reguitti et al. 2026a), there is no compelling evidence for organic dust in the red novae environments except for CK Vul.

4 https://www.euroalma2040.com/
